# Pilot Studies Testing Novel Minimized Pan-Coronavirus (CoV) Vaccines in Feline Immunodeficiency Virus-Infected Cats With or Without Feline CoV Serotype-1 (FCoV1) Coinfection and in Specific-Pathogen-Free Cats Against Pathogenic FCoV2

**DOI:** 10.3390/vaccines13111172

**Published:** 2025-11-18

**Authors:** Pranaw Sinha, Marco B. Prevedello, Ananta P. Arukha, Valentina Stevenson, Karen F. Keisling, Taylor G. Nycum, Nina M. Beam, Elise D. Barras, Bikash Sahay, Janet K. Yamamoto

**Affiliations:** 1 Department of Comparative, Diagnostic, and Population Medicine (CDPM), College of Veterinary Medicine, University of Florida, Gainesville, FL 32610, USA; sinhapranaw@ufl.edu (P.S.); marco.prevedello@ufl.edu (M.B.P.); stevensovalentin@ufl.edu (V.S.); keisling@ufl.edu (K.F.K.); nycumtaylor@ufl.edu (T.G.N.); nina.beam@ufl.edu (N.M.B.); 2 Laboratories of Comparative Immunology & Virology of Companion Animals, CDPM, College of Veterinary Medicine, University of Florida, Gainesville, FL 32610, USA; ananta.arukha@ufl.edu (A.P.A.); sahayb@ufl.edu (B.S.); 3 Department of Infectious Diseases and Immunology, College of Veterinary Medicine, University of Florida, Gainesville, FL 32610, USA; 4 Animal Care Services, University of Florida, Gainesville, FL 32610, USA; e.barras@ufl.edu

**Keywords:** pan-coronavirus vaccine, feline coronavirus serotype-1, SARS-CoV-2, feline coronavirus serotype-2

## Abstract

**Background**: The minimized pan-coronavirus (CoV) vaccine-1 developed by our laboratory contained pDNA sequences of feline coronavirus serotype-1 (FCoV1) and SARS-CoV2 (SCoV2) spike B-cell epitopes plus FCoV/SCoV2-conserved, CoV-specific polymerase cytotoxic T-lymphocyte (CTL) epitopes formulated in lipid nanoparticle (LNP). Only FCoV2 infects feline cell lines needed for developing native challenge inoculum that causes feline infectious peritonitis (FIP). Hence, Pilot Study 1 evaluated the therapeutic efficacy and safety of the pan-CoV vaccine-1 in feline immunodeficiency virus (FIV)-infected cats, with or without FCoV1 coinfection. Pilot Study 2 evaluated the cross-protective effect of pan-CoV vaccines in specific-pathogen-free (SPF) cats against intranasal challenge with FIP virus serotype 2 (FIPV2). **Methods**: In Study 1, we vaccinated two FIV-infected cats (one negative and another positive for FCoV1 coinfection) intramuscularly twice with CTL epitopes-LNP vaccine and later twice with pan-CoV vaccine-1. Controls included two unvaccinated FIV-infected cats with or without FCoV1 coinfection. Study 2 assessed the sequential vaccinations of three pan-CoV vaccines in four SPF cats. The first two vaccinations were with pan-CoV vaccine-2, followed by pan-CoV vaccine-3 (twice), and lastly with pan-CoV vaccine-1 (once). Three SPF controls included two cats immunized with LNP and one lacking any immunization. Pan-CoV vaccine-2 contained pDNAs with modified FCoV1/SCoV2 B-cell epitopes plus CTL epitopes in LNP. Pan-CoV vaccine-3 contained only pDNAs with FCoV1 B-cell epitopes plus CTL epitopes in LNP. **Results**: Study 1 demonstrated no adverse effect with 25 μg and 50 μg CTL epitopes-LNP vaccine and 50 μg pan-CoV vaccine-1. However, 100 μg pan-CoV vaccine-1 caused fever 24 h later, which was resolved by a single Meloxicam treatment. Both vaccinees developed cross-FCoV2 neutralizing antibodies (XNAbs), immunoblot binding antibodies (bAbs) to FCoV1 receptor-binding domain (RBD), and T-cell responses to FCoV1 RBD, whereas one vaccinee also developed bAbs to SCoV2 RBD. Study 2 demonstrated no adverse effects after each vaccination. Three vaccinees developed low-titer XNAbs and bAbs to FCoV2 spike-2 by the fourth vaccination. Upon challenge, all cats developed FCoV2 NAbs and bAbs to FCoV2 nucleocapsid and RBD. High vaccine-induced T-cell responses to FCoV1 RBD and T-cell mitogen responses declined with an increase in responses to FCoV2 RBD at three weeks post-challenge. Two of the three controls died from FIP, whereas one vaccinee, with the lowest vaccine-induced immunity, died from skin vasculitis lesions and detection of FIPV2 infection by semi-nested RT-snPCR in feces. **Conclusions**: In Pilot Study 1, the pan-CoV vaccine-LNP dose of 50 μg had no adverse effects, but adverse effects were observed at 100 μg dose. In Pilot Study 2, the FCoV1-based B-cell vaccine(s) induced low levels of XNAbs against FIPV2 and delayed challenge infection against high-dose FIPV2. The high-dose FIPV2 infections in the vaccinated and control cats started to clear, by single housing at 23–26 weeks post-challenge, whereas two cats in Pilot Study 1 cleared natural FCoV1 transmission by 26 weeks post-infection.

## 1. Introduction

Feline coronaviruses serotype-1 and 2 (FCoV1, FCoV2) are alpha-coronaviruses that differ from beta-coronaviruses of SARS-CoV-2 (SCoV2) variants which also infect domestic cats [1,2,3,4]. Both FCoV1 and FCoV2 can mutate into feline infectious peritonitis viruses (FIPV1, FIPV2) which infect macrophages and cause lethal clinical diseases [5,6]. The metabolite (GS-441524) of the Remdesivir prodrug (Veklury from Gilead Sciences, Inc., Foster City, CA, USA), which works against SCoV2 in humans, has successfully treated FIP in pet cats, but the drug treatment is expensive and does not always clear infection [7,8,9]. Zoetis Inc. has marketed attenuated, temperature-sensitive FCoV2/FIPV2 vaccine, but its ability to cross-protect against FCoV1 and FIPV1 infection is unclear [10,11]. The importance of developing a vaccine that protects against FCoV1 and subsequent FIPV1 infection is vital, since FCoV1 infects domestic cats in the U.S. and the world more than FCoV2 [5,6]. One difficulty of developing an FCoV1 vaccine is that FCoV1 does not infect feline cell lines at elevated levels [12,13]. In comparison, FCoV2 infects multiple feline cells at prominent levels without losing its FIP pathogenicity [13]. Consequently, a challenge inoculum with a high titer of FCoV1/FIPV1 is not readily available. The alternative of an FCoV1 challenge is to use either (i) FCoV1-infected cats as a source of FCoV1 infection through fecal contact studies of FCoV1-based vaccinated cats or (ii) FCoV1 vaccine as a therapeutic treatment in FCoV1-infected cats, which is the approach of the current Pilot Study 1.

Domestic cats are readily infected fecal-orally with FCoV from other FCoV-infected cats and oral-nasally with SCoV2 from SCoV2-infected owner(s) [14,15,16,17]. We have reported that FCoV1-infected cats develop cross-reactive binding antibodies (XbAbs) to the SCoV2 receptor-binding domain (RBD) [18]. Therefore, combining FCoV1 and SCoV2 spikes as B-cell epitopes may have a synergistic effect against both viruses. The pDNA-lipid nanoparticle (LNP) vaccine should last longer than the mRNA-LNP vaccine used in COVID-19 [19]. However, packaging pDNAs with both full-length spikes in LNP will limit the load of RBD, which possesses the sites that induce FCoV/SCoV2-neutralizing antibodies (NAbs) [18,19,20]. Furthermore, the SCoV2 spike possesses neurotoxin, inflammatory intracellular adhesion molecule-1 (ICAM-1), inflammatory superantigen, and bullous pemphigoid (BP) motifs which may contribute to the adverse effects observed with the commercial full-length spike vaccines for humans [21,22]. Hence, a pan-coronavirus (CoV) vaccine with minimized FCoV1 and SCoV2 spike B-cell epitopes, as well as FCoV/SCoV2-conserved cytotoxic T-lymphocyte (CTL) epitopes, should induce B-cell and CTL immunity against both viruses. SCoV2 protein-adjuvant vaccine, consisting of RBD conjugated to heptad repeat 1 (HR1) and connected to HR2 (RBD-HR1-HR2 with adjuvant), has successfully induced broad-spectrum NAbs, including SCoV2-delta NAbs, and protected ACE2-mice and rhesus macaques against challenge with Omicron and SCoV2 delta variants, respectively [23,24]. These results suggest that the RBD-HR1-HR2 structure may display a better conformation and less toxicity than vaccination with only the RBD mRNA-LNP vaccine [25,26].

Extrapolating from the three studies described above [24,25,26], the current research developed three minimized FCoV1 B-cell epitopes and two minimized SCoV2 epitopes in three pan-CoV pDNA-LNP vaccines. The B-cell epitopes had major molecular modifications by including stem helix (SH), transmembrane (TM), and cytoplasmic tail (CT) sequences. We evaluated these minimalized pan-CoV vaccines for their immunogenicity, safety, and efficacy as therapeutic vaccines against FCoV1 and as possible cross-reacting prophylactic vaccines against FCoV2. Current studies also assessed our two hypotheses regarding the inability of commercial human COVID-19 vaccines in preventing SCoV2 infection. Our first hypothesis is that the negative effects of the adverse regions on the spike and/or LNP caused the vaccine-induced side effects. As a result, the induction of focused protective immune responses diminished, while disease severity decreased. However, these vaccines were still unable to prevent SCoV2 infection. Such infections allow these viruses to persist in humans and cats by mutating and evolving (e.g., more pathogenic SCoV2 variants and FIPVs). Our second hypothesis is that the virus-specific CTL epitopes will cause complete inhibition of SCoV2 and FCoV1 infections. Based on human COVID-19 vaccines, we speculate that the minimized spike may not possess sufficient conserved CTL epitopes, due to the considerable natural mutations that occur on the N-terminal end of the spike [18,27]. The minimalization of the spike sequence will also remove the CTL epitopes. Thus, FCoV/SCoV2-conserved CTL epitopes from highly conserved viral enzymes, such as RNA-dependent RNA-polymerase (RdRp), must be included. Two pilot pan-CoV vaccine studies were performed in (1) the feline immunodeficiency virus (FIV)-infected cats, with or without FCoV1 coinfection, to evaluate the therapeutic immunogenicity and safety of the FCoV1-based pan-CoV vaccine-1 and (2) the SPF cats for the immunogenicity and safety of the sequential vaccination of three pan-CoV vaccines against intranasal challenge with heterologous subtype-2 FCoV2/FIPV2-UCD2. These two pilot studies on pan-CoV vaccines include a limited number of laboratory cats due to the following reasons: (1) In Pilot Study 1, we had only four SPF cats infected for six years with FIV when they were accidentally coinfected with FCoV1 from neighboring FCoV1-infected laboratory cats. (2) The safety of the FCoV1/SCoV2-based pan-CoV pDNA-LNP vaccine was evaluated against FIPV2-UCD2, as Pilot Study 2, with only seven SPF cats available (four vaccinated and three control).

## 2. Materials and Methods

### 2.1. Animals

Our two pilot domestic cat studies used two sets of laboratory cats which were initially specific-pathogen-free (SPF) cats (1) inoculated with FIV for six years before coinfection with FCoV1 (HOK, HOL, HON, HOO) from neighboring FCoV1-infected laboratory cats and (2) vaccinated with three FCoV1-based pan-CoV vaccines (2FM, 2FN, 2FP, FB1) and immunized with LNP (2FL, 2FO), over the duration of 8.9 months before challenge, with FCoV2/FIPV2-UCD2. All four SPF cats in Pilot Study 1 were from the same lineage and litter, bred under IACUC 201401838. They were inoculated with FIV, treated with antiretroviral drugs, and maintained under IACUC 20120253, 201502530, 201702530, 2020002530, and 202200000332. The five SPF female cats (2FL-2FP) and two SPF male cats (FB1 and 2FR) were bred under IACUC 201701838, 202001838, and 202300000347, and vaccinated and inoculated with FIPV2-UCD2 under IACUC 202300000512. The SPF male cat FB1 in Pilot Study 2 was derived from a slightly different lineage of the same tom but related queen (tom D4H and queen 2FB). One additional younger control male cat (2FR) in Pilot Study 2 was from generation-4 litter, from the same lineage (tom D4H and queen Y2F) as the five cats 2FL-2FP (generation 3). The control male (2FR) was inoculated with the same dose of FIPV2-UCD2 as all cats in Pilot Study 2. Three additional SPF cats (2FB, 2FI, and 2FG), of the same lineage but of different generation littermates as 2FL-2FP, served as the prospective age-matched weights of the unvaccinated SPF cats in Pilot Study 2. Our SPF cats were weighed weekly as mandated by IACUC protocols 202001838 and 202300000347. Table S1 shows the laboratory cats used for Pilot Studies 1 and 2.

### 2.2. Immunoblot Strips for CoV RBDs and FIPV2 Whole-Virus

The production of immunoblot strips and the purification of SCoV2, FCoV1, and FCoV2 RBD antigens for the strips were previously described [18]. The FIPV2 UCD2-whole-virus (WV) was produced and purified as previously described for FCoV2 WSU79-1146-WV, along with its corresponding immunoblot strips [18]. Since we did not have a live FCoV1 isolate, we used live purified FIPV2-UCD2 isolated from an FIPV-infected field cat as whole virus in our immunoblot strips. FIPV2-UCD2 immunoblot strips were used to evaluate vaccine-induced XbAbs to FIPV2-UCD2 S2 and nucleocapsid (NC). Remarkably, a chronically FCoV1-infected laboratory cat (UGA4, hereon called UG4) produced sera-containing XbAbs to spike (S) 240 kDa, S 160 kDa, S2 90 kDa, and NC 45 kDa [18] (Figure 10B, UG4). HALO Quantitative Image Analysis (Albuquerque, NM, USA) was used to detect the percent (%) intensity of the immunoblot bands (original immunoblot strips and gels in Supplemental Materials) [28].

### 2.3. Cross-Neutralizing Antibodies (XNAbs) to FIPV2-UCD2 Using Fc9 Cell Line

We did not have culture-adapted FCoV1 virus for the NAb assay or live FCoV1 virus for the challenge study. Instead, we inoculated FIPV2-UCD2 as the virus for the XNAb assay using the feline embryonic-9 (Fc9) cell line, as previously performed with FCoV2-WSU79-1146 for NAb assay against FCoV2 [18]. The sera from vaccinated and LNP-control cats were incubated using the same titration method, with a set concentration of FIPV2-UCD2 in place of FCoV2-WSU79-1146, as described in detail in our previous study [18].

### 2.4. Feline IFNγ and IL-2 ELISpot Analyses

The feline IFNγ and IL-2 ELISpot analyses were performed as previously described [29,30], using the purified SCoV2, FCoV1, and FCoV2 RBDs, and purified FIPV2 UCD2-WV for immunoblot analyses.

### 2.5. Developing and In Vitro Testing of Minimized pan-CoV Vaccines

#### 2.5.1. Developing B-Cell Epitopes of Minimized pan-CoV Vaccines

**Figure 1:**
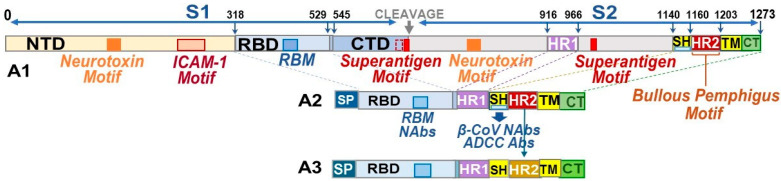
**Schematic figure of a minimized SARS-CoV-2 (SCoV2) spike protein.** The structural organization of the SCoV2 spike protein. (A1) displays the adverse regions residing throughout the spike 1 (S1) and (S2). The adverse sequences are the neurotoxin, intracellular adhesion molecule 1 (ICAM-1), superantigen, and bullous pemphigus (BP) motifs. The complete N-terminal domain (NTD), the majority of the C-terminal domain (CTD), and the regions of spike 2 (S2) protein, except for the five key sections, are deleted from the final B-cell construct. The B-epitope construct consists of the receptor-binding domain (RBD), 16-aa section of CTD, heptad repeat 1 (HR1), stem helix (SH), HR2, transmembrane (TM), and cytoplasmic tail (CT) sections, with the addition of a signal peptide (SP) on the N-terminal of NTD. The feline IL-2 promoter constitutes the SP protein (A2,A3). Due to the presence of the BP sequence in the entire sequence of HR2, SCoV2 HR2 is exchanged with HR2 from a distinctly related αCoV (A3).

**Figure 2:**
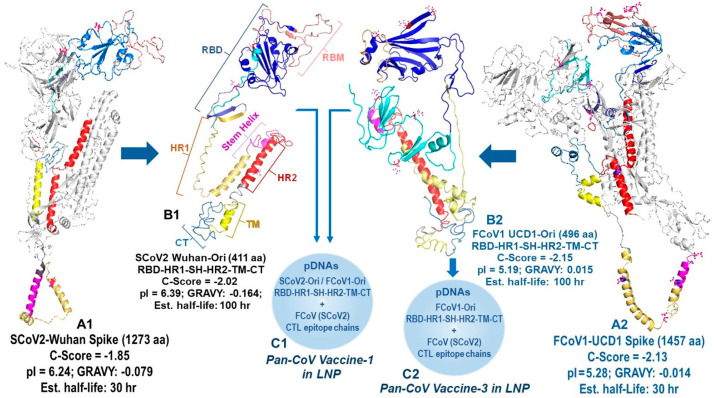
**Production of the foundation SCoV2-Wuhan and FCoV1-UCD1 B-cell constructs.** The models of the full-length spike proteins for SCoV2-Wuhan (**A1**) and FCoV1-UCD1 (**A2**) were constructed using the I TASSER server. The gray regions were molecularly removed to derive the original B-cell epitopes, called SCoV2 Wuhan-Ori (**B1**) and FCoV1 UCD1-Ori (**B2**). The aa sequences of the RBD-HR1-SH-HR2-TM-CT construct for SCoV2-Wuhan (411 aa) and FCoV1 (496 aa) were input individually into the I-TASSER server to obtain the homology model, first for SCoV2 Wuhan (**B1**) and subsequently for FCoV1 UCD1 (**B2**). The RBD (blue), N-terminal RBD (cyan), receptor-binding motif (RBM) (salmon), and SH (magenta) provide the protective B-cell epitopes, stimulating anti-CoV antibody production. The HR1 and HR2 stems serve as helical pillars of the mounted RBD and allow the development of the construct trimer. The TM stabilizes the construct in the cell membrane, while the CT resides in the cell cytoplasm to provide intracellular signaling. The RBD with two (**B1**) or six (**B2**) N-glycosylation sites are shown, each with red asparagine (N). The nucleotide sequences of feline multivalent pan-CoV vaccine-1 and pan-CoV vaccine-2 were composed of FCoV1 and SCoV2 B-cell construct pDNAs (**C1**) and FCoV1 B-cell construct pDNA (**C2**), respectively. The two B-cell construct pDNAs were mixed together with pDNAs of three different FCoV/SCoV2-conserved CTL/TH epitope chains, and all delivered in a lipid nanoparticle (LNP).

All B-cell epitopes for minimized pan-CoV vaccines were developed by assuring the configuration of the protein structure resembled the SCoV2 RBD-HR1-HR2 protein as previously reported by He, C. et al. [24]. The SCoV2 RBD was configured to resemble the RBD on full-length SCoV2 spike, as depicted by artificial intelligence (AI)-based I TASSER homology model (Ann Arbor, MI, USA and Kent Ridge, Singapore) [31] and diagramed with Schrodinger PyMOL 2.5 and 3.0.3 systems (New York, NY, USA) [32]. Our goal was to delete two neurotoxin, one ICAM-1, two inflammatory superantigen, and one bullous pemphigus (BP) motif from the SCoV2 spike [21,22]. One neurotoxin motif was on the N-terminal domain (NTD) of spike 1 (S1), and the second neurotoxin motif was on the S2, next to the fusion peptide between S1-S2 cleavage site and HR1 (Figure 1) [21]. Since He, C. et al. deleted this section completely and retained NAb epitopes most likely on RBD [24], we opted to do the same and connected SCoV2 RBD with short C-terminal domain of the S1 sequence (STNLVKNKCVNFNFNG) to the N-terminal of HR1. Furthermore, the first neurotoxin motif and inflammatory ICAM-1 motif were both on the NTD of S1; therefore, the entire NTD was removed. Hence, our B-cell epitope protein consisted of SCoV2 RBD-HR1-SH-HR2-TM-CT with minor changes made at the connection sequence between RBD and HR1 and between HR1 and stem helix (SH). The connection sequence was made more hydrophilic to expose the RBD and SH outward to react with potential NAbs or antibody-dependent cellular cytotoxicity antibodies (ADCC Abs) [33]. We used the Expansy ProtParam tool of Swiss Institute of Bioinformatics (Lausanne, Switzerland) [34] to select the connection points and make minor changes between the two key structures, RBD and HR1, and between HR1 and SH. Multiple sequence submissions were made to I TASSER to obtain a sequence with models with a C-Score between −2.25 and −1.25. C-Score, ranging from −5 to 2, is the confidence estimation to assess how likely a predicted model matches the native protein structure. A higher C-Score depicts more reliable prediction, while a lower C-Score suggests lower confidence. Since our structure has four major deletions of a full-length spike, we expected a C-Score from −2.25 to −1.25 to be within the range for a reliable prediction model. The full-length SCoV2-Wuhan and FCoV1-UCD1 had a C-Score of −1.85 and −2.13, respectively (Figure 2). I TASSER provided the C-Score for each of their top five models and the Protein Database Bank (RCSB PDB) file (Piscataway, NJ, USA) of their top 10 models.

The Expansy ProtParam tool provided the total amino acids (aa), grand average of hydropathicity (GRAVY), theoretical isoelectric point (pI), estimated half-life, and instability index to identify stable sequences. Our goal was to produce models which had biochemical properties of a full-length spike of SCoV2-Wuhan and FCoV1-UCD1. The full-length SCoV2-Wuhan and FCoV1-UCD1 spikes had a theoretical pI of 6.24 and 5.28, respectively, and an estimated half-life in reticulocytes of >30 h (Figure 2). Numerous SCoV2 and FCoV1 RBD-HR1-SH-HR2-TM-CT sequences were evaluated and the best sequences with the following criteria were chosen: (1) C-Score of −2.25 to −1.25, (2) aa sequence close to a third of the full-length spike, (3) GRAVY of −0.170 to −0.00, (4) acidic pI of 5.5-to-6, (5) estimated half-life of 100 hr, and (6) an instability index showing stable sequence. We have made exceptions for FCoV1 UCD1-Ori and FCoV1 UCD1-sRBD. FCoV1 UCD1-Ori has the largest RBD region to allow for both SCoV2 and FCoV1 receptor-binding motifs (RBMs) to remain intact [18]. Our first reason for a large RBD is that the mutation sites affecting the FCoV2 NAbs have been reported by others [35,36]. One NAb extends between N-terminal of FCoV1 UCD1-Ori in pan-CoV vaccine-1 and N-terminal of FCoV1-sRBD in pan-CoV vaccine-2 (Figure S1). The second reason for a large RBD is that the FCoV1-UCD1-Ori of pan-CoV vaccine-1 has an N-terminal region with an aa sequence identity and similarity to a FCoV2-UCD2 RBD sequence. Lastly, our analysis of deletion of the extremely hydrophobic TM region and the deletion of TM-CT resulted in GRAVY of −0.104 and −0.111, respectively, suggesting that the external region of FCoV1 UCD1-Ori will remain exposed to the intracellular fluid of Golgi apparatus and endoplasmic reticulum (ER) once the TM binds to their membrane.

#### 2.5.2. Developing CTL Epitopes for All pan-CoV Vaccines

**Figure 4:**
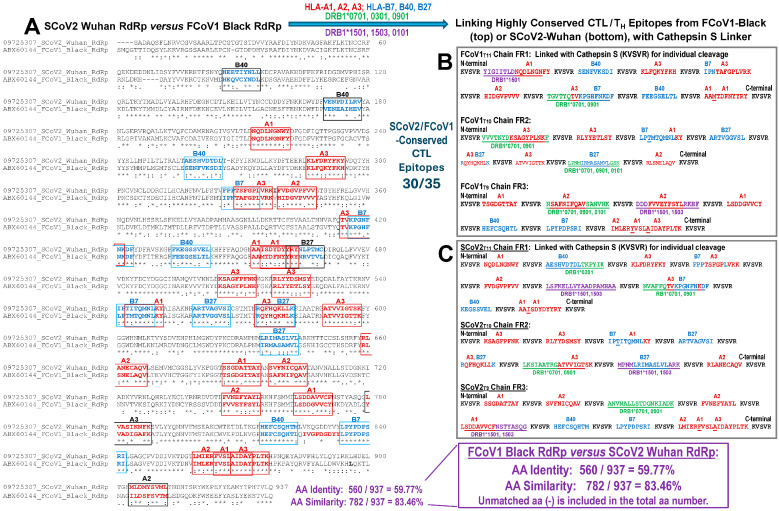
**Mapping of 9-mer CTL and 15-mer TH epitopes on Wuhan and FCoV1 RdRp sequences.** On the alignment (**A**), each box represents two 9-mer CTL epitopes with aa residues conserved between Wuhan (Accession #YP_009725307.1) and FCoV1 (Accession #ABX60144.1). The signs below each vertical pair of amino acids represent identical (*), similar (:), and slightly similar (.) amino acids. HLA allotype epitopes are enclosed in red boxes, and HLA-B allotype epitopes are in blue boxes. The CTL epitopes are combined with TH epitopes to develop three chains each for FCoV1 (**B**) and Wuhan (**C**). TH epitopes (green/purple and underlined aa residues with or without CTL overlap) bind to HLA-DRB1 as described below. The cathepsin S linker (KVSVR) is inserted between the epitopes as a cleavage site for the enzyme, cathepsin S.

The CTL 9-mer epitopes were identified on FCoV1-Black RdRp and SCoV2-Wuhan RdRp using NetMHCpan 4.1 and NetCTL 1.2 of the Dutch Technology Institute (DTU, Lyngby, Denmark) [37,38]. The FCoV1-Black sequence was used because FCoV1-UCD1 RdRp has not been published. The T-helper (TH) 15-mer epitopes overlapping those CTL peptide sequences were determined using NetHMCIIpan 4.0 and NetHMCII 2.3 of DTU [39,40]. The RdRp sequences were aligned using the JustBio Clustal O (1.2.4) multiple sequence alignment program (AgileBio LLC, San Diego, CA) [41]. The CTL 9-mer epitopes were plotted by a rectangular box with human leukocyte (HLA) allotype on the top and two CTL 9-mer peptides within the box (Figure 4A). Only those with the highest aa sequence similarity or identity, especially at the HLA pocket sites, were selected [42]. Five CTL epitopes within a black box were not conserved and not included in the three chains of CTL epitopes. Each chain included 9-mer HLA class-I peptides interspersed with cathepsin S linker sequence (KVSVR) between each CTL peptide or between overlapping CTL peptides and a CTL peptide (Figure 4B, FCoV1 chains; Figure 4C, SCoV2 chains). All HLA class-I boxes of CTL epitopes were aligned to the JustBio Clustal O (1.2.4) multiple sequence alignment for FCoV1-Black RdRp and FCoV2-WSU79-1146 RdRp (Figure S2A). This alignment identified those CTL 9-mers conserved between FCoV1 and SCoV2 and also conserved between FCoV1 and FCoV2. Subsequently, the DRB1 TH 15mer epitopes which overlap the two CTL epitopes were chosen (Figure 4B,C and Figure S2B). Exceptions were made for the SCoV2_T9_ chain with added DRB1*0701 plus *0901 epitopes and the SCoV2_T11_ chain with added DRB*1501 plus *1503 epitopes (Figure 4C). The FCoV1_T10_ chain, without DRB*1501 plus *1503 epitopes, replaced with additional DRB*0701 plus *0901 epitopes (Figure 4B).

The selected HLA class-I and class-II alleles are more commonly present in the U.S. population. The common HLA class-I alleles include HLA-A1, HLA-A2, HLA-A3, HLA-B7, HLA-B40/B44, and HLA-B27 [43]. The common HLA class-II alleles include DRB1*0701, DRB1*1501, DRB1*0301, DRB1*1503, DRB1*0901, and DRB1*0101 [44]. These HLA allotypes were chosen to determine the CTL and TH epitopes on CoV RdRp. Furthermore, a feline leukocyte antigen (FLA) algorithm server is currently not available. Domestic cats can recognize HLA-A2 and HLA-27 peptides [45]. Hence, the servers for human HLA alleles have been used to characterize the surrogate allotypes of the CoV peptides recognized by cats. Current pilot studies have evaluated the T-cell ELISpot analyses of FCoV1, FCoV2, and SCoV2 RBDs, as previously described [29], but did not test CTL peptides due to a limited number of peripheral blood mononuclear cells (PBMCs) isolated from cats.

#### 2.5.3. The 35 CTL Peptide Epitopes for the First Two Vaccinations in Study 1

The CTL peptide epitopes were selected from FCoV2 NC and RdRp using NetMHCpan 4.1 and NetCTL 1.2 [37,38], as described above for selection of CTL peptides on RdRp (Section 2.5.2). SCoV2-Wuhan NC aa sequence and FCoV2-WSU79-1146 aa sequence were aligned using the JustBio alignment program (Figure S3A) [41]. This CTL peptide chain was called FCoV2-CTL35, because its sequence was based on FCoV/ScoV2-conserved FCoV2 RdRp and NC sequences (Figure 4B and Figure S3B).

#### 2.5.4. Plasmid Construction

The B-cell epitope nucleotide (nt) sequence consisted of short Kozak nt sequence (GCTAGCGCCACC) connected to feline IL-2 promoter nt sequence (ATGGCATATAAAATTCAACTTCTTAGTTGTATTGCACTTACTCTTATTCTTGTTACTAAT) on its 5′-end. The B-cell epitope nt sequence was inserted upstream of the enhanced green fluorescent protein (*eGFP*) in the pDNA, pCI-H2H-*eGFP* (Addgene Inc., Watertown, MA, USA). In contrast, the CTL peptide nt chains had a short Kozak nt sequence on their 5′-end, but no feline IL-2 promotor nt sequence.

#### 2.5.5. Production of pDNA-LNP-Based pan-CoV Vaccines

The plasmid concentration was made to 50 μg/mL in an aqueous buffer. LipidFlex^TM^ (PreciGenome LLC, San Jose, CA, USA) was mixed with 4-(dimethylamino)-butanoic acid, (10Z,13Z)-1-(9Z,12Z)-9,12-octadecadien-1-yl-10,13-nonadecadien-1-yl ester (DLin-MC3-DMA, MedChemExpress LLC, Monmouth Junction, NJ, USA) in ethanol to generate the lipid mixture. The ratio of LipidFlex to DLin-MC3-DMA was predetermined by testing several ratios to determine DNA encapsulation ability using gel retardation assay. The lipid nanoparticles containing the plasmid (pDNA-LNP) were constructed using NanoGenerator Flex-S (PreciGenome LLC, San Jose, CA, USA). The formulated pDNA-LNPs were dialyzed against DNase-free water and resuspended in PBS. The size and uniformity of the pDNA-LNP were tested using NanoPartica SZ-100V2 (Horiba, Ltd.; Kyoto, Japan), which was uniformly 150 nm in size. The pDNA-LNP was freshly prepared for each vaccination and injected within six hours of manufacturing.

#### 2.5.6. In Vitro Testing of Minimized pan-CoV Vaccines in Feline Cell Line

The feline cell line used was feline embryonic-9 (Fc9) cells. Both FCoV2-79-1146 and FCoV2-UCD2 readily infected this cell line. Four B-cell construct pDNAs and a pooled pDNAs preparation of FCoV_T9_, FCoV_T10_, and FCoV_11_ chains were individually transfected into a 60% confluent monolayer of Fc9 cells in 25-cm^2^ flask. We used the Takara Xfect^TM^ Transfection Reagent and the transfection method, as described by Takara Bio USA, Inc. (San Jose, CA, USA) [46]. Briefly, 4 μg of our viral pDNA, suspended in Xfect Reaction Buffer, was incubated with Takara’s nanoparticle complexes for 10 min before transfection of Fc9 cells. Upon transfection of 4 h at 37 °C in 5% CO_2_, the pDNA medium was discarded, and the cells were cultured in fresh culture medium for an additional 48 h. After trypsinization of the confluent monolayer of cells, the cell suspension was washed thrice in PBS before testing for GFP expression by flow cytometry using Cytek 3-Laser Aurora.

### 2.6. FIPV RT-PCR and Immunohistochemistry for Detection of FIPV2

The primer sets for NSP14 and RdRp were used to analyze the feces from FIPV2-inoculated cats. The purified pooled FIPV2-UCD2 culture medium sample was used as positive control. An NSP14 primer set of 5′-GTGATGCTATCATGACTAG-3′ Forward and 5′-CACCATTACAACCTTCTAA-3′ Reverse was produced by Integrated DNA Technologies (IDT), which amplified 417 bp on gel [47]. The RdRp primer set of 5′-GGATGGGATATCCAAAGTGTGA-3′ Forward and 5′-CCATCATCAGACAAAATCATCATA-3′ Reverse amplified 440 bp on gel for the first cycle of semi-nested RT-snPCR [48]. The same forward RdRp primer was used together with an inner 5′ACTGTAACATTGTTACAAGC-3′ reverse primer to amplify a 313 bp band in the second cycle of RT-snPCR. Both NSP14 RT-PCR and RdRp RT-snPCR-amplified bands were captured into gel images by Bio-Rad Gel Doc Imager and quantified as percentage intensity using HALO Quantitative Image Analysis (original immunoblot strips and gels in Supplemental Materials). All amplified sequences were confirmed by sequencing performed by Eurofins Genomics (Louisville, KY, USA). The RT-PCR analysis on tissue lesions from an FIPV2-infected cat was performed by the UF Zoological Medicine Diagnostic Laboratory using a previously described method [49]. The immunohistochemistry was performed on tissue lesions from an FIPV2-infected cat by the UF Veterinary Diagnostic Laboratories using Bio-Rad MCA2194 (Clone FIPV3-70; murine monoclonal IgG2a to FIPV1 and FIPV2 NC, Hercules, CA, USA).

### 2.7. Pilot Study 1

The first-generation feline pan-CoV vaccine-1 was formulated as a therapeutic vaccine against FCoV1 in an FIV/FCoV1-coinfected laboratory cat, HOL. This vaccine is also a prophylactic against FCoV1 infection in the FIV-infected laboratory cat, HOK. Both HOK and HOL have been receiving triple AIDS drugs (Tenofovir Disoproxil Fumarate at 20 mg/kg BID) (twice a day), Lamivudine at 50 mg/kg BID, and Raltegravir at 50 mg/kg BID since 2019. Two age-matched cats (HON, HOO), challenged with the same inoculum and dose of FIV-FC1 at the same time as HOL and HOK in 2018, served as the unvaccinated FIV-infected control cats. All four FIV-infected cats were infected with FCoV1 in 2020 and again in late 2022. All four FIV-infected cats cleared the FCoV1 infection by single housing upon first FCoV1 exposure. However, upon second exposure, HOK and HOO cleared FCoV1 infection by single housing, but HOL and HON did not clear FCoV1 and were coinfected with FCoV1. Vaccinations of HOK and HOL in Study 1 were initiated when they were 6.5 years old and terminated at approximately 7.5 years old.

**Figure 6:**
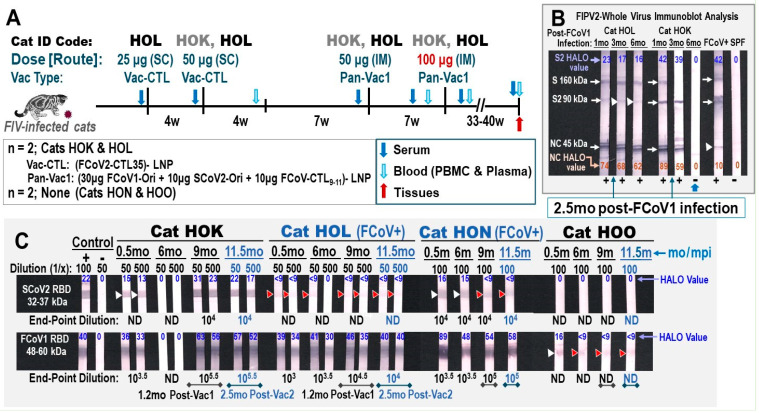
**Safety and immunogenicity of feline pan-CoV vaccine-1 vaccination.** (**A**) The vaccination schedule of two FIV-infected cats (FCoV1-free/FIV-infected HOK and FCoV1/FIV-coinfected HOL) is shown with the vaccine dose in μg, vaccine type, and vaccination route as either subcutaneous (SC) or intramuscular (IM). The serum (blue arrow), blood (light-blue arrow) for PBMC and plasma, feces (green arrow), and tissues (red arrow) are collected at the time points shown with the designated arrow. The samples from unvaccinated FCoV1/FIV-coinfected cat HON and unvaccinated FCoV1-free/FIV-infected cat HOO were collected at the same time as those collected from vaccinated cats. (**B**) The XbAbs to FIPV2-WV for vaccinated cats HOK and HOL at 1:50 dilution are shown for serum/plasma collected at 1, 3, and 6 months post-FCoV1 infection (6 mpi), which occurred before vaccination. Both HOK and HOL were single housed at 2.5 mpi. HALO Quantitative Image displayed the strong bands with an arrow for one or more strips at 24–74% intensity and the weak bands with a white arrowhead at 10–17% intensity (HOL S2, FCoV+ UG4 NC). (**C**) The serum/plasma of all four cats were evaluated for the bAbs to SCoV2 RBD (top) and FCoV1 RBD (bottom) at 1:50 and 1:500 dilutions for vaccinated cats HOK and HOL and 1:100 dilution for unvaccinated cats HON and HOO. The HALO intensity values of all strong bands without arrowheads are 30–89% intensity for FCoV1 RBD and 17–31% for SCoV2 RBD. The weak bands with a white arrowhead are 13–16% intensity. The immunoblot strips have the brightness and contrast intensities of 8% and 0% for (**B**), respectively, and both 5% for (**C**). The original Immunoblot strips can be found in Supplemental Materials.

The first two vaccinations of HOL (25 μg and 50 μg) and the first vaccination of HOK (50 μg) were administered subcutaneously (SC) with LNP containing a single chain of 25 FCoV1 CTL epitopes, called Vac-CTL (Figure 6A). The next two vaccinations of HOK and HOL were administered intramuscularly (IM) with the pan-CoV vaccine-1. This vaccine was tailored for safety study on vaccine dose and contained 50 μg and 100 μg of immunogen pDNAs in the first and second vaccinations, respectively. The 50 μg dose of immunogen pDNA contained 30 μg FCoV1-UCD1 RBD-HR1-SH-HR2-TM-CT (FCoV1-Ori), 10 μg SCoV2-Wuhan RBD-HR1-SH-HR2-TM-CT (SCoV2-Ori), and 10 μg FCoV/SCoV2-conserved CTL epitope chains (three FCoV-CTL_9-11_ chains; each with 9, 10, or 11 epitopes per chain). The vaccine dose of 100 μg is the same ratio of pDNAs, which is equivalent to double the amount of 50 μg dose. The schedule shows the timing, route, and dose of the Vac-CTL and Pan-Vac1 vaccinations in the weeks post-first vaccination (Figure 6A).

### 2.8. Pilot Study 2

**Figure 9:**
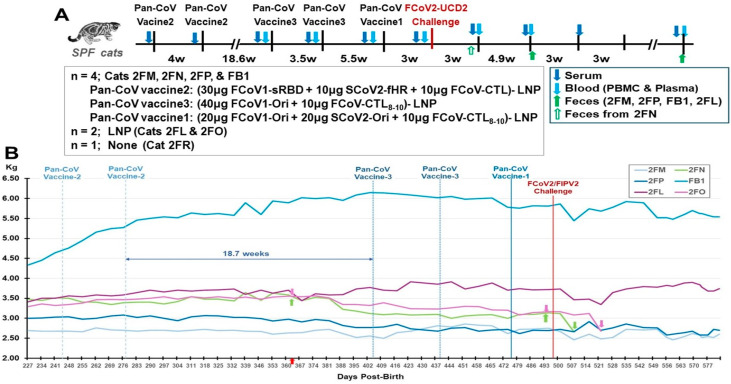
**Safety and immunogenicity of three feline pan-CoV vaccines in Pilot Study 2.** The vaccination schedule of the first two vaccinations with pan-CoV vaccine-2, followed by two vaccinations with pan-CoV vaccine 3, and a last single vaccination with pan-CoV vaccine-1 are shown (**A**). The collections of serum (blue arrow), blood (light-blue arrow) for PBMC and plasma, and feces (green arrow) are made at the time points shown with the designated arrow on the schedule. The number of SPF cats, with their identification code, is shown for vaccine, LNP-control, and unvaccinated groups immediately below the schedule. The compositions of each pan-CoV vaccine, with their amount, are shown next to each vaccine. The weights of each cat were measured weekly during vaccination and twice a week after one wpc (**B**). The weights of four vaccinated cats and two LNP-control cats are shown from day 227 to day 577 post-birth. The red arrow on day 364 at the X-axis represents all cats at one year old. This figure does not include the SPF kitten 2FR since he was not age-matched with the six SPF cats. The first magenta arrow for 2FO and the first green arrow for 2FN show the time point (day 375) when they had the highest weight at post 1-year-old. The second arrows represent the time point on day 495, which is three days before the challenge on day 498. The last arrows for 2FN and 2FO are on days 508 and 522, respectively, which mark the day of humane euthanasia.

Four SPF cats at 35 weeks old (females 2FM, 2FN, 2FP) and 30 weeks old (male FB1) were vaccinated sequentially with pan-CoV vaccine-2 twice, followed by pan-CoV vaccine-3 twice and, lastly, pan-CoV vaccine-1 once, before being challenged 3 weeks later with FIPV2-UCD2 (Figure 9A). Control SPF cats included the 35-week-old, generation-3 littermates (females 2FL and 2FO) and the younger generation-4 littermate male 2FR (16-week-old). Our goal was to use the same (2FL-2FP, 2FR) or close (FB1) littermates (Section 2.1) which will ensure the pattern of weight gains or losses is similar. Two other generations of littermates (generation-1 2FB; generation-2 2FG and 2FI) were used as unvaccinated SPF cats for retrospective age-matched weights.

The first two vaccinations at an interval of four weeks were with the pan-CoV vaccine-2, which had the smallest FCoV1 RBD (sRBD), but with an identical minimized S2 (HR1-SH-HRT2-TM-CT) structure as the next two vaccine (Figure 9A). Our goal was to vaccinate first with the smallest FCoV1 RBD, followed by the larger full-length FCoV1 RBD. A rest interval of 18.7 weeks was allowed to ensure all cats were over one year old before receiving the next two (3rd and 4th) vaccinations with pan-CoV vaccine-3, a full-length FCoV1 RBD connected to the minimized S2 structure. The pan-CoV vaccine-3 contained no SCoV2 B-cell epitopes to focus the immunity towards FCoV1 B-cell epitopes on RBD-HR1-SH-HR2-TM-CT structure. All pan-CoV vaccines contained FCoV/SCoV2-conserved CTL epitope chains with TH peptides to generate CTL activities against the challenge virus, FIPV2-UCD2. The purified FIPV2-UCD2 in PBS at a dilution titer of 10^6^ was administered intranasally (0.1 mL per each nostril) at three weeks post-last the fifth vaccination with pan-CoV vaccine-1. The immune analyses of vaccinated and control cats at pre-challenge were FIPV2-UCD2 XNAb analysis (Section 2.3) and immunoblot-based FIPV2 XbAb analyses (Section 2.2). The immune analyses at post-challenge were direct NAb to FIPV2-UCD2, bAbs to FIPV2-WV and FCoV2 RBD, and indirect bAbs to FCoV1 RBD. The feline IFNγ and IL-2 ELISpot analyses with PBMC from vaccinated and LNP-control cats at pre- and post-challenge evaluated the T-cell responses to SCoV2, FCoV1, and FCoV2 RBDs, and to ConA. Fecal FIPV2-UCD2 load was performed by FIPV2 RT-snPCR (Section 2.6), whereby cycle 1 and cycle 2 of RT-snPCR quantitated the viral load by HALO Quantitative Image Analysis [29].

### 2.9. Statistical Analyses

The statistical comparison between the vaccine group and the combined control group was determined by a two-tailed Fisher’s exact test. The statistical comparison of the in vitro results was based on a two-tailed paired *t*-test for ELISpot studies and a two-tailed unequal variance (Welch’s) *t*-test for duplicate HALO results of immunoblot and RT-snPCR studies. All analyses were considered statistically significant when *p* < 0.05.

## 3. Results

### 3.1. Developing and In Vitro Testing of Minimized Pan-CoV Vaccines

#### 3.1.1. Developing B-Cell Epitopes for pan-CoV Vaccine-1

We developed the B-cell epitopes for three minimized pan-CoV vaccines using AI-based I TASSER homology modeling. We identified the structural conformation of the minimized spike protein comparable to the original full-length spike, especially at the outward trajectory of RBM on the RBD. The B-cell epitopes consisted of RBD connected to HR1-SH-HR2-TM-CT structure where the stem helix (SH), transmembrane (TM), and cytoplasmic tail (CT) were added to the reported SCoV2 RBD-HR1-HR2 protein vaccine [23,24]. Such minimalization reduces the size by a third of the full-length spike and removes two neurotoxin, two superantigen, and one ICAM-1 motifs (Figure 1). Identical regions were deleted from the FCoV1 B-cell epitopes. The order of structural organization for the RBD-HR1-SH-HR2-TM-CT is identical to that of the original spike for both FCoV1 and SCoV2. The original FCoV1-UCD1 B-cell epitopes (FCoV1-Ori) in pan-CoV vaccine-1 consisted of FCoV1-UCD1 RBD, previously described in [18], and were connected to FCoV1-UCD1 HR1-SH-HR2-TM-CT. In addition, the original SCoV2-Wuhan RBD, as previously described in [18], was connected to the SCoV2-Wuhan HR1-SH-HR2-TM-CT and included in the pan-CoV vaccine-1. Both FCoV1 and SCoV2 B-cell epitopes included SH, because SCoV2 SH serves as a conserved βCoV NAb epitope(s) and ADCC epitope [33]. Furthermore, the added TM and CT will retain the B-cell epitopes on the membrane of endoplasmic reticulum (ER) of the transinfected host cell, mimicking the scenario of spike expressed during SCoV2 infection [50]. The structure of RBD-HR1-SH-HR2-TM-CT of both FCoV1 and SCoV2 was adjusted at the connection sequences between each region using the best structural model from I TASSER homology modeling (Figure 2, next page). The details of B-cell epitope construction are described in Section 2.5.1.

#### 3.1.2. Developing B-Cell Epitopes for pan-CoV Vaccine-2

**Figure 3:**
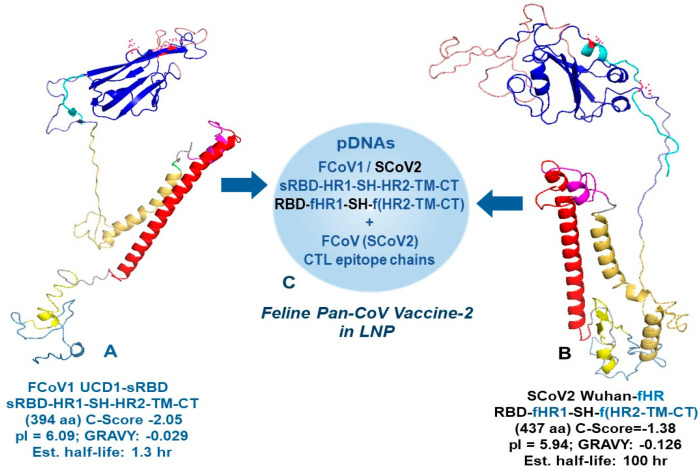
**The minimized FCoV1 UCD1-sRBD and SCoV2 Wuhan-fHR constructs for human pan-CoV vaccine-2.** The left view of the FCoV1 UCD1-sRBD (**A**) and right view of the recombinant SCoV2 Wuhan-fHR (**B**) are shown together with their sequence (Seq) code, structure (exchanges in blue for recombinant), C-score, and biochemical features (aa number, pI, GRAVY, and estimated half-life). FCoV1 UCD1-sRBD has a shortened RBD attached to the minimized S2 HR1-SH-HR2-TM-CT, similar to the one for FCoV1 UCD1-Ori. SCoV2 Wuhan-fHR is a SCoV2 recombinant with FCoV1 HR1 and FCoV1 HR2-TM-CT exchanges. The RBD with two (**B**) or three (**A**) N-glycosylation sites are shown, each with red asparagine (N). The pDNAs of these B-cell constructs are mixed with FCoV/SCoV2-conserved CTL epitope chains in the LNP (sphere display), resulting in the final human pan-CoV vaccine (**C**).

The FCoV1 B-cell epitopes in the pan-CoV vaccine-2 consisted of a short FCoV1-UCD1 RBD (FCoV1-sRBD) connected to the FCoV1 HR1-SH-HR2-TM-CT (Figure 3, next page), identical to the one used in pan-CoV vaccine-1. The shortened FCoV1-sRBD had the amino-end deleted from the RBD, leaving the carboxyl-end with the RBM similar to that of human αCoV-NL-63, which uses human ACE2 as its cell receptor (Figure S1) [51,52]. The pan-CoV vaccine-2 also contained the original SCoV2-Wuhan RBD connected to a recombinant (FCoV1-UCD1 HR1)-SCoV2 SH-(FCoV1-UCD1 HR2-TM-CT). This hybrid structure was developed to remove the BP motif on SCoV2 HR2, while retaining SCoV2 SH with βCoV NAb epitope(s) (Figure 1 and Figure 3B). FCoV1-UCD1 HR1 and HR2 replaced both SCoV2 HR1 and HR2, since FCoV1 HR2 is devoid of BP. The resulting SCoV2/FCoV1 recombinant B-cell epitope construct, called SCoV2- fHR, was configured as the best structural model by using I TASSER modeling and the Expansy ProtParam tool (Figure 3B).

#### 3.1.3. Developing B-Cell Epitopes for pan-CoV Vaccine-3

The pDNA for FCoV1 B-cell epitopes in the pan-CoV vaccine-3 was identical to the pDNA for FCoV1-Ori RBD-HR1-SH-HR2-TM-CT (Figure 2B2) used in pan-CoV vaccine-1. The pan-CoV vaccine-3 contained 40 μg of FCoV1-Ori pDNA by excluding the pDNA for SCoV2 B-cell epitopes. Consequently, this vaccine was used to assess the immunogenicity of the FCoV1-Ori.

#### 3.1.4. Developing CTL Epitopes for All pan-CoV Vaccines

The initial CTL epitopes used in Pilot Study 1 were derived from both the FCoV-conserved RdRp and nucleocapsid peptide sequences. A single chain of 25 peptide sequences (FCoV-CTL25) consisted of cathepsin S linker (KVSVR; cat-SL) sequences interspersed between each peptide sequence (Figures S2 and S3) [53]. A long single chain can bundle and may not expose the chain as a linear sequence. Thus, the cathepsin S enzymes in the cytoplasm and endosome are unable to lyse at the unexposed cat-SL to release the CTL peptides. Therefore, three short chains containing 9, 10, and 11 CTL peptides from RdRp, with each peptide interspersed with cat-SL (Section 2.5.2), were individually inserted into the pCI-H2H-*eGFP*. These FCoV1 and FCoV2 CTL peptides are identical and possess high aa similarity to SCoV2 CTL peptides (Figure 4A). Few of the CTL peptide sequences in each chain contained an overlap of TH epitope (Figure 4B) to enhance the expression of CTL peptides in the host cells.

#### 3.1.5. The In Vitro Expression of Minimized pan-CoV Vaccines in Feline Cell Line

**Figure 5:**
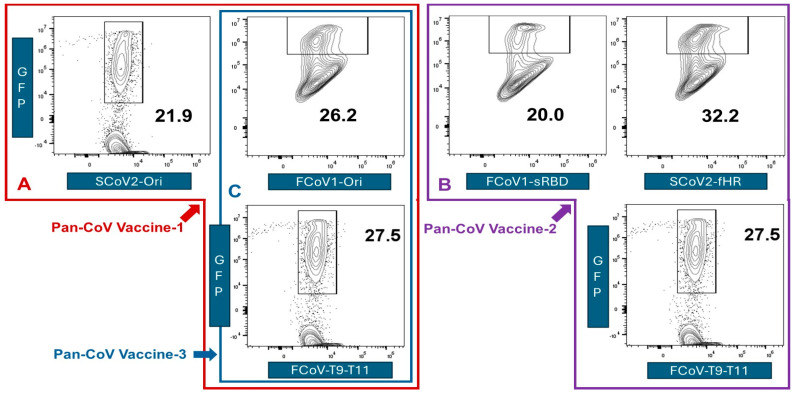
**In vitro testing of pan-CoV vaccines in Fc9 cells.** The SCoV2-Ori pDNA, FCoV1-Ori DNA, and three pDNAs of pooled FCoV1-T9-T11 chains (Figure 4B) were combined into LNPs as pan-CoV vaccine-1 at 2–4 h before in vitro transfection. FCoV1-T9-T11 chain sequences are named conserved FCoV-T9-11 chain sequences because FCoV1 and FCoV2 aa sequences are identical except for three pairs of peptide epitopes, each with one aa similarity (Figure S2A). The individual vaccine-transfected Fc9 cells are evaluated by flow cytometry for GFP expression. The flow cytometry profiles for pan-CoV vaccine-1 display a GPF expression of 21.9% for SCoV2-Ori, 26.2% for FCoV1-Ori, and 27.5% FCoV-T9-T11-transfected cells (**A**). Three flow cytometry profiles for pan-CoV-2 display a GFP expression of 20.0% for FCoV1-sRBD, 32.2% for SCoV2-fHR, and 27.5% for FCoV-T9-T11 (**B**). Panels A and B have the three flow cytometric profiles bracketed within 90-angled L-shaped red (**A**) and purple (**B**) boxes. The two flow cytometry profiles for pan-CoV vaccine-3 display a GFP expression of 26.2% for FCoV1-Ori and 27.5% for FCoV-T9-T11, which is bracketed with a vertical rectangular blue box (**C**).

We transfected pDNAs containing these B-cell and CTL epitope nt sequences into the *Felis catus* embryonic-9 cells (Fc9 cells) and measured the GFP expression by flow cytometry (Figure 5), as detailed in Section 2.5.6. The highest GFP expression was observed with SCoV2-fHR pDNA, followed by three pooled pDNAs of FCoV CTL peptide chains, FCoV1-Ori pDNA, SCoV2-Ori pDNA, and lastly, the FCoV1-sRBD pDNA. Based on the in vitro expression levels, the best three pDNAs to combine against FCoV1 challenge were FCoV1-Ori and SCoV2-fHR for FCoV1/SCoV2 B-cell epitopes and the pooled FCoV-conserved CTL peptide epitopes.

### 3.2. Pilot Study 1: Testing of pan-CoV Vaccine-1 in FIV-Infected Cats With or Without FCoV1 Coinfection

#### 3.2.1. Pilot Study 1: Schedule, Cat Distribution, and Vaccine-Induced Adverse Effects Four

SPF cats (HOK, HOL, HON, HOO) were inoculated six years earlier with a Florida isolate, FIV-FC1 [45,54]. Two cats (HOK, HOL) were subsequently treated daily with triple-AIDS drugs. All four FIV-infected cats were coinfected with FCoV1 from neighboring FCoV1-infected laboratory cats [18]. These FIV/FCoV1-coinfected cats were individually housed to clear FCoV1 infection. Only two cats (HOK, HOO) cleared FCoV1 infection by 6 mo post-FCoV1 exposure, based on the clearance of antibodies (Abs) to FCoV1 RBD and XbAbs to FCoV2 NC. All cats were asymptomatic except for one coinfected cat (HOL), which developed chronic diarrhea controlled by daily oral prednisolone during the Pilot Study. Therefore, this cat needed the therapeutic pan-CoV vaccine to decrease her diarrhea. HOL received the first two vaccinations at a 4-week interval with FCoV-CTL25-LNP, followed 12 weeks later with 50 μg pan-CoV vaccine-1, and then 5 weeks later with 100 μg pan-CoV vaccine-1 (Figure 6A). HOK received a single vaccination of FCoV-CTL25-LNP, and the remaining vaccinations were identical to those administered to HOL. Both HOK and HOL received triple-AIDS drugs daily throughout the Pilot Study. The collection of serum and blood from unvaccinated controls HON and HOL occurred at the same time as those shown for vaccinated cats. Adverse vaccine reactions to 100 μg of pan-CoV vaccine-1 were fever, fast pulse and respiration rates, lethargy, and loss of appetite, which occurred 24 h post-vaccination in both cats. They were immediately treated with a single low dose of Meloxicam which resolved all adverse effects by the next day. No major weight loss was observed in the vaccinated cats (Figure S4).

#### 3.2.2. Pilot Study 1: Pan-CoV Vaccine-1-Induced Antibodies

The pan-CoV vaccine-1-induced antibody immunity of vaccinated cats (HOK, HOL) was compared to those produced by chronic FCoV1 infection (HON) (Figure 6B, HOK and HOL shown). The vaccinated cat HOL had a chronic FCoV1 infection and prednisolone treatment which made assessing the vaccine immunogenicity and efficacy more difficult. The bAbs to FCoV1 RBD immunoblot demonstrated that all four cats at 0.5 mo post-FCoV1 exposure had FCoV1 RBD bAbs. The FCoV1 RBD bAbs completely or almost disappeared by 6 mo in HOK and HOO, respectively (Figure 6C). In contrast, both HOL and HON retained high titers of FCoV1 RBD bAbs at 6 mo post-exposure and higher titers thereafter at 9 mo and 11.5 mo post-exposure. Although the SCoV2 RBD-HR1-SH-HR2-TM-CT pDNA load in the vaccine was lower than that of FCoV1 RBD-SH-HR2-TM-CT pDNA, the vaccinations still induced a high titer of SCoV2 RBD bAbs in the vaccinated cat HOK, but only a negligible titer in the vaccinated HOL. Unexpectedly, the chronically FCoV/FIV-coinfected cat HON developed an almost undetectable titer of cross-reactive SCoV2 RBD bAb(s) at 1:100 dilution which decreased with time.

**Figure 7:**
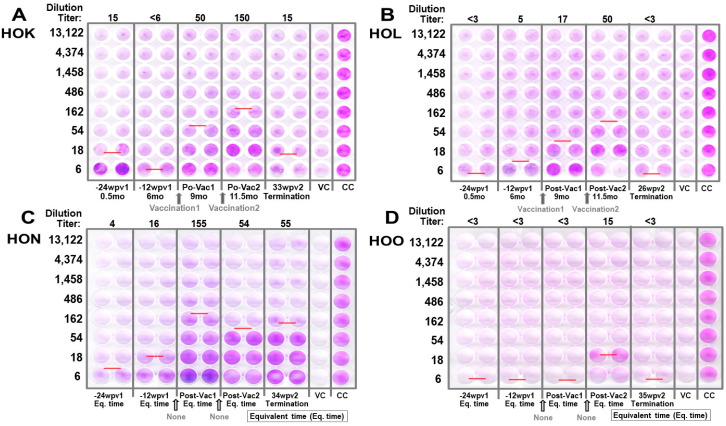
**FCoV2-UCD2 XNAbs of cats in Pilot Study 1.** Four crystal violet-stained plates for HOK (**A**), HOL (**B**), HON (**C**), and HOO (**D**) are shown. The virus control lane (VC) and uninfected cell control lane (CC) are shown in lanes 11 and 12, respectively. The intensity of crystal violet purple staining demonstrates the viable cells remaining after serum Abs neutralized the FIPV2 infection. The red line represents an estimate of XNAb dilution titer for the duplicate wells, which is also shown at the top of the plate as dilution titer. The time point of the serum used is shown at the bottom of the plate for each duplicate well. The serum dilutions for each row of wells are shown on the left of the plate from the lowest (bottom) to highest (top). At the bottom of the plate, the time of first pan-CoV vaccine-1 (vaccination 1) is between −12 weeks post-first vaccination (−12 wpv1) and post-first vaccination 1 (Po-Vac1) for HOK (**A**) and HOL (**B**). The equivalent time points for Vaccination 1 and Vaccination 2 are shown with an arrow between −12 wpv1 and Post-Vac1 and another arrow between Post-Vac1 and Post-Vac2 for HON (**C**) and HOL (**D**). The XNAb analysis for HOO, upon repeated testing, showed no titer at all time points, including Post-Vac 2 Eq. time.

FCoV1 virus was not available at the time of this study. Therefore, the sera from vaccinated and control cats were evaluated for XNAbs against FIPV2-UCD2 at various time points of pre- and post-vaccinations. Remarkably, both HOK and HOL had vaccine-induced XNAbs, but the unvaccinated/FCoV1-negative HOO had none (Figure 7A,B,D). The most unexpected results observed were in the unvaccinated/FCoV1-positive HON who had consistently high XNAbs throughout the study. These elevated results persisted even on the day of euthanasia of HON (Figure 7C).

#### 3.2.3. Pilot Study 1: T-Cell Immunity Induced by FCoV1/SCoV2 RBDs

**Figure 8:**
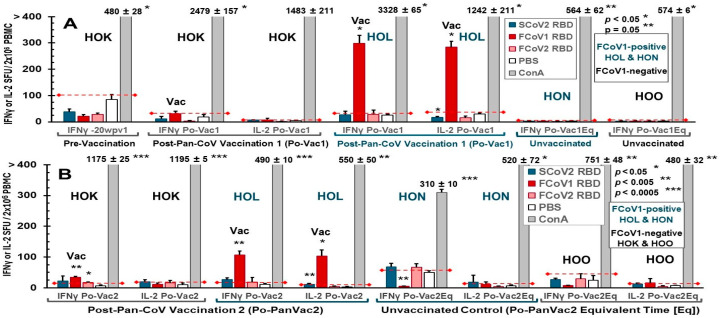
**Feline IFNγ and IL-2 ELISpot analyses of cats in Pilot Study 1.** The IFNγ and IL2 ELISpot responses to SCoV2 (blue bar), FCoV1 (red bar), and FCoV2 (salmon bar) RBD stimulations are shown at post-first pan-CoV vaccination (Po-Vac1) for vaccinated cats HOK and HOL or equivalent time (Po-Vac1Eq) for unvaccinated cats HON and HOO (**A**). Additionally, HOK’s IFNγ responses at pre-vaccination of -20 weeks post-first pan-CoV vaccination are also shown on the left as the first set. The control stimulations include concanavalin A (ConA) as a positive T-cell mitogen and the PBS diluent as a negative control. The IFNγ/IL-2 responses of PBMC from all four cats to the same RBDs are shown at post-second pan-CoV vaccination (Po-Vac2) for HOK and HOL or equivalent time (PoVac2Eq) for HON and HOO (**B**). Those average responses with significant differences from the average value of the negative control at *p* < 0.05, *p* < 0.005, or *p* < 0.0005 are shown with (*), (**), or (***) above the bar.

A limited number of PBMC can be isolated from the blood of these cats. As a result, only the IFNγ and IL-2 productions of the T-cell ELISpot responses to FCoV1, FCoV2, and SCoV2 RBDs and T-cell mitogen concanavalin A (ConA) were compared to the PBS control after each pan-CoV vaccine-1 vaccination (Figure 8A,B). Vaccinated cat HOK had no significant increase in IFNγ and IL-2 responses to all RBD at post-first pan-CoV vaccination (Figure 8A) and a slight but significant increase in IFNγ at post-second pan-CoV vaccination (Figure 8B). In contrast, vaccinated cat HOL developed significant increases in both IFNγ and IL-2 responses to FCoV1 RBD at post-first pan-CoV vaccination and lower but significant increases at post-second vaccination (Figure 8B). Both control cats HON and HOO had no response to any of the RBDs at the time, equivalent to post-first and post-second vaccinations. Furthermore, both vaccinated cats developed substantially to significantly higher cytokine responses to ConA than those of unvaccinated control cats (Figure 8A). The vaccinated HOK retained high cytokine responses to ConA at post-second pan-CoV vaccination, but the vaccinated HOL decreased in cytokine responses to ConA at levels similar to those of the control cats at post-second vaccination (Figure 8B).

### 3.3. Pilot Study 2: Testing of pan-CoV Vaccines in SPF Cats

#### 3.3.1. Pilot Study 2 Schedule, Vaccine-Induced Adverse Effects, and Clinical Signs Post-Challenge

Four SPF cats (2FM, 2FN, 2FP, FB1) were vaccinated sequentially with pan-CoV vaccine-2 twice, followed by pan-CoV vaccine-3 twice, and, lastly, with pan-CoV vaccine-1 once, before challenge 3 weeks later with FIPV2-UCD2 (Figure 9A). The control SPF groups consisted of two cats immunized with LNP (2FL, 2FO) at the same time as the vaccination of the vaccine group and one cat with no immunization (2FR). No adverse reaction was observed after each vaccination, except for weight loss (Figure 9B). Three vaccinated cats (FB1, 2FN, 2FP) at post-third vaccination and LNP-immunized cat 2FO started to lose weight. Vaccinated cat 2FM at post-fourth vaccination and LNP-immunized cat 2FL also started to lose weight. All vaccinated and LNP-control cats had sudden loss of weight starting at 10 days post-challenge (dpc). One vaccinated cat 2FN developed a lesion on top of her head at 9 dpc which had a scratch marking and was treated with triple-antibiotics ointment daily and single oral Convenia next day. This cat was immediately single housed, but the lesion expanded, her weight declined, and she developed a fever of 104 °F in the morning of 16 dpc. She was treated with prednisolone and diphenhydramine, which resolved the fever in the afternoon. She was euthanized the next day, since her lesions spread to the neck (Figure S5), and she had lost 15% of her weight, when compared to the weight measured immediately before challenge. However, her weight loss was 25.3% when compared to the peak weight on Day 375 before the third vaccination. Necropsy of this cat was unremarkable with no signs of FIP disease. The lesions were analyzed by immunohistology and by pan-coronavirus RT-PCR but were negative for FIPV2.

The LNP-immunized control cats had the most weight loss of 10.5% (2FL) and 15.2% (2FO) on Day 522 (25 dpc) when compared to the weight immediately before challenge (day 495) (Figure 9B, Table 1). Cat 2FO was euthanized due to the severe weight loss of 15.2%, and necropsy showed pleural fluid in her lung cavity. The younger control cat (2FR) developed fever and tachycardia 5 days post-challenge (dpc) and was treated with Meloxicam. However, by 7 dpc, 2FR displayed ocular opacity, lethargy, hunching, and problem with micturition. Cystocentesis confirmed urinary tract infection (UTI) based on cocci bacteria in the urine from the bladder. 2FR was immediately treated with the antibiotic Convenia, which resolved the clinical symptoms for close to three weeks, but fever reoccurred with deep abdominal breathing and hunching for micturition at 29 dpc. He was retreated with Convenia but could not resolve the symptoms and had a 9.8% weight loss when compared to the weight on 21 dpc, as well as an X-ray showing thoracic fluid on the day of euthanasia at 36 dpc. Necropsy results showed FIP disease signified by pleural effusion, fluid in the heart, and multiorgan lesions typical of FIP lesions (Figure S6). Comparison of weight loss and peak S2 XbAbs summarized in Table S2. Furthermore, four challenged live cats displayed FIP-related complete blood counts and blood chemistry (Table S3).

#### 3.3.2. Pilot Study 2: B-Cell-Based pan-CoV Vaccine Abs

**Figure 10:**
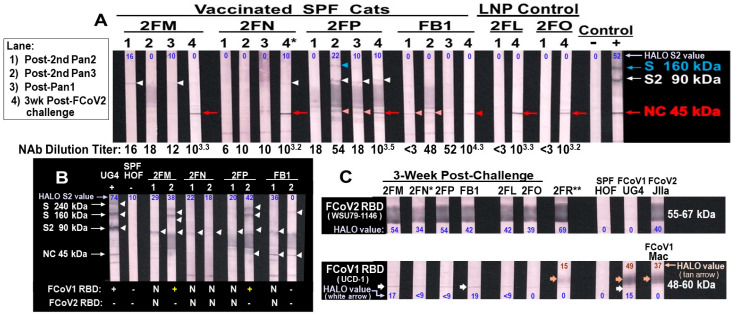
**Vaccine-induced XbAbs to FIPV2-WV and post-challenge bAbs to FCoV2 RBD of the vaccinated and control cats.** (**A**) The sera/plasma, collected at post-second vaccination with pan-CoV vaccine-2 or empty LNP (lane 1), post-2nd vaccination with pan-CoV vaccine-3 or empty LNP (lane 2), post-vaccination with pan-CoV vaccine-3 or empty LNP (lane 3), and at 3 wpc (lane 4), are reacted to low-dose (4 μg/strip), FIPV2-WV immunoblot strips. The bAb reactivities with blue arrowhead (17% intensity), white arrowhead (10–22%), light red arrowhead (18–35%), and red arrow (34–55%) or arrowhead (17%) point to the S 160 kDa, S2 90 kDa, unknown 45.5 kDa (light red arrowhead), and NC 45 kDa bands, respectively. (**B**) The high-dose (6 μg/strip) immunoblot strips reacting with serum/plasma from vaccinated cats, at post-second vaccination with pan-CoV vaccine-2 (lane 1) and at post-second vaccination with pan-CoV vaccine-3 (lane 2). All vaccinated cats have S2 band for both strips, except FB1 with only strip 1. 2FM and 2FP show additional binding to the S 240 kDa and S 160 kDa bands, whereas FB1 has weak S 160 kDa on strip 2. (**C**) The serum collected at 3 wpc from three vaccinated cats (2FM, 2FP, FB1) and two LPN-control cats (2FL, 2FO) show strong reactivity to FCoV2-WSU79-1146 RBD ((**C**), top) and a negligibly thin band to FCoV1-UCD1 RBD for 2FM and FB1 ((**C**), bottom, white arrow). Vaccinated cat 2FN’s serum from 2.3 wpc (*) (euthanasia day) and unimmunized control 2FR’s serum at 6.7 wpc (**) show a strong reaction to FCoV2 RBD ((**C**), top: 2FN, 2FR), comparable to the positive control plasma from FCoV2-inoculated cat JIIa. Cats 2FN’s and 2FR’s serum reactivities to FCoV1-UCD1 RBD display a light thin band (white arrow), while 2FR has an additional weak glycosylation band (orange arrow). However, this reactivity is not comparable in intensity to the FCoV1-infected controls, UG4, and pet Mac ((**C**), bottom, orange arrow). All FCoV2-WV and RBD immunoblot analyses are performed at 1:50 dilution, except for FCoV2-infected control JIIa at 1:100,000. The UG4 and HOF sera served as positive and negative controls, respectively, for all panels, with panel (**C**) (bottom only for UG4; top and bottom for HOF). The % intensity of bands determined by HALO Quantitative Image Analyses (shown in Original Immunoblot Strips and Gels in Supplemental Materials).

Since we did not have FCoV1-WV immunoblot strips, we used FIPV2 UCD2-WV immunoblot strips instead to evaluate vaccine-induced XbAbs to FCoV2 S, S2, and NC. Only vaccinated cat 2FM had XbAbs to S2 (90 kDa) at post-second vaccination with pan-CoV vaccine-2 on low-dose FCoV1-WV strip (Figure 10A, lane 1). In contrast, serum from vaccinated 2FM, FB1, and 2FN had strong XbAb reactivity to S2 on high-dose FCoV1-WV strips, but 2FP had a weak S2 XbAb reactivity (Figure 10B, lane 1). The sera from cats 2FM and 2FP, post-second vaccination with pan-CoV vaccine-3, developed strong XbAbs to S 240kDa and S 160kDa, and FB1 also developed weak XbAbs to S 160kDa on high-dose FIPV2-WV immunoblot strips (Figure 10B, lane 2). Only vaccinated 2FP retained the XbAbs to S 160kDa on a low-dose FCoV2-WV immunoblot strip (Figure 10A, lane 2). All vaccinated and control cats developed bAbs to FIPV2 NC (45 kDa) and FIPV2 NAbs at 3 weeks post-challenge (wpc) (Figure 10A), indicating that all cats were most likely infected with FIPV2-UCD2. As confirmation of previous findings, the serum from 3 wpc were tested for bAbs to FCoV2 RBD and bAbs to FCoV1 RBD (Figure 10C). All challenged cats tested strongly positive for bAbs to FCoV2 RBD (Figure 10C, top) but had negligible bAb reactivities to FCoV1 RBD (Figure 10C, bottom). The serum from unimmunized control 2FR demonstrated slightly higher XbAb reactivities to FCoV1 RBD, but not to the intensities of serum bAbs from FCoV1-infected cats UG4 and pet-Mac. Furthermore, sera from 2FM and FB1 had a slightly stronger single thin band of bAb reactivity(s) to FCoV1 RBD than the control cats, suggesting remains of FCoV1 bAb(s) from vaccination.

#### 3.3.3. Pilot Study 2: T-Cell Immunity Induced by FCoV1/SCoV2 RBDs of B-Cell Constructs

**Figure 11:**
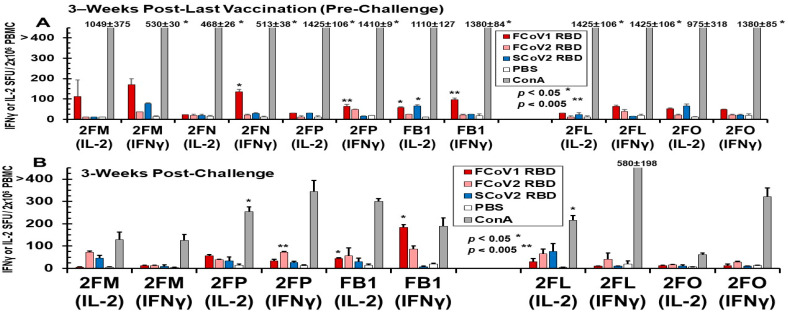
**Feline IFNγ and IL-2 ELISpot analyses of cats in Pilot Study 2.** The IFNγ and IL2 ELISpot responses to SCoV2 (blue bar), FCoV1 (red bar), and FCoV2 (salmon bar) RBD stimulations are shown at 3 weeks post-last pan-CoV vaccination or LNP-immunization, before challenge, for vaccinated and LNP-control cats, respectively (**A**). The control stimulation included concanavalin A (ConA) as a positive T-cell mitogen and the PBS diluent as a negative control. The IFNγ/IL-2 responses to the same RBD stimulations of the PBMC from five live cats are shown at 3 wpc (**B**). Those average responses with significant differences from the average value of the negative control at *p* < 0.05 or *p* < 0.005 are shown with (*) or (**) above the bar.

The PBMC collected from vaccinated and LNP-immunized cats immediately before challenge developed IL-2 and IFNγ T-cell responses to FCoV1 RBD, which were consistently higher in vaccinated cats than in LNP-immunized cats (Figure 11A). The PBMC from three vaccinated cats (2FN, 2FP, FB1) developed statistically higher responses to FCoV1 RBD than their PBS control. All cats, except for 2FN (IL-2 and IFNγ) and 2FM (IFNγ), developed robust IL-2 and IFNγ responses to the T-cell mitogen, ConA. The PBMC from all cats, except for euthanized cat 2FN, at 3 wpc decreased by 2.5–15-fold in IL-2 and IFNγ responses to ConA (Figure 11B). Additionally, all cats, except for 2FO with minimum responses, had predominantly lower responses to FCoV1 RBD at 3 wpc, while inversely developing higher responses to FCoV2 RBD at 3 wpc than those at pre-challenge. The increase in responses to FCoV2 RBD at 3 wpc suggests that all vaccinated and LNP-immunized live cats were infected with FIPV2.

#### 3.3.4. Pilot Study 2: FIPV2 Challenge Infection Demonstrated by Fecal FIPV2 Load and High-Dose FIPV2 Immunoblot Strips

**Figure 12:**
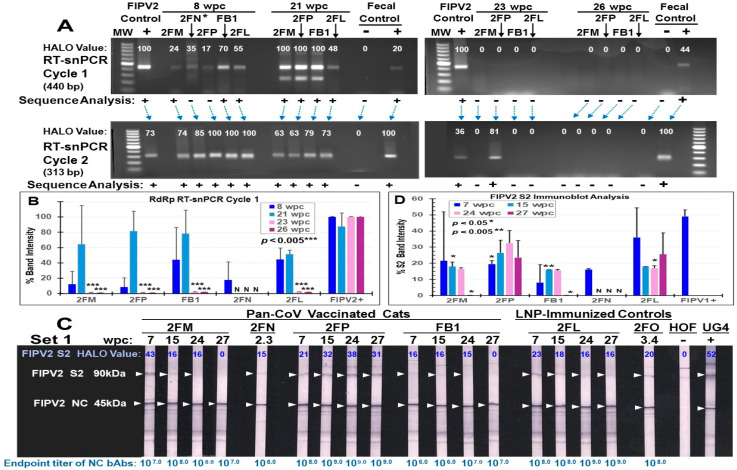
**The fecal FIPV2 load and serum bAbs from the vaccinated and LNP-control cats after challenge.** The fecal samples from vaccinated live cats (2FM, 2FP, FB1) and LNP-control live cat (2FL) at 8, 21, 23, and 26 wpc were monitored for FIPV2-UCD2 shedding using RdRp RT-snPCR cycle 1 ((**A**), top gels) and cycle 2 ((**A**), bottom gels). A fecal sample from 2FN (*) was collected from the colon at 2.3 wpc during necropsy. The bar graph (**B**) of HALO results from cycle 1 is shown as a comparison between average value of individual cat and average value of corresponding FIPV2-UCD2 control (FIPV2+). Fecal positive at 8 wpc and negative controls were from the FB1′s and SPF cat’s reverse-transcribed cDNAs, respectively. The sera were collected from all four live cats at 7, 15, 24, and 27 wpc and two cats, 2FN (2.3 wpc) and 2FO (3.4 wpc), on the day of euthanasia (**C**). All sera were tested at 1:200 dilution using high-dose (6 μg per strip) immunoblot strips. Reciprocal endpoint dilution titers (1/X) of NC bAbs are shown below each strip. The positive and negative control sera (incubated at 1:50 dilution) were collected from FCoV1-infected UG4 and SPF HOF, respectively. The brightness and contrast intensities were 5% (**C**). The bar graph of HALO average values derived from panel 12C and Figure S8C shows sporadic statistical difference between the average value at each time point and the average value of FIPV1-positive control (**D**). No significant difference was observed between S2 band intensities of each vaccinated cat and those of LNP-control 2FL. The original gels can be found in Supplemental Materials.

Fecal samples collected from 2FN at 2.3 wpc on the day of euthanasia, and all remaining live cats (three vaccinated 2FM, 2FP, and FB1, and LNP-control 2FL) at 8 wpc were all positive by FIPV2 RdRp RT-snPCR upon cycle 2 (Figure 12A, top left gel). Although samples from 2FN and 2FP each have a light band at cycle 1, their amplicons from cycle 1 were negative by FIPV2 RdRp sequencing. The same fecal samples tested by FIPV2 NSP14 RT-PCR were negative for the predicted 417 bp band, when the FIPV2-UCD2 control was positive (Figure S8A). Two vaccinated cats (2FM, 2FP) had a delay in fecal shedding of 440 bp RdRp as shown in cycle 1 at 8 wpc, which peaked in cycle 1 at 21 wpc, like the remaining live cats (Figure 12B, top left gel). As expected, fecal samples from all live cats had a 313 bp band in cycle 2 and were all positive by sequencing at 21 wpc. Fecal samples from 23 to 26 wpc became negative by RdRp RT-snPCR in both cycles, except 2FP in cycle 2 at 23 wpc (Figure 12A, right top gel).

Immunoblot analysis of FIPV2 S2 bands demonstrates loss of S2 bAbs at 27 wpc in two vaccinated cats (2FM, FB1) (Figure 12C, 27wpc). The mean intensity values of duplicate tests at 7 wpc are lower for all vaccinated cats than LNP-control 2FL, whereby vaccinated FB1 has the lowest mean value (Figure 12D). Cat 2FM’s first test shows strong S2 band (Figure 12C), whereas her second test shows a weak S2 band (Figure 12C), leading to a high standard deviation (Figure 12D). The band intensity values show a steady decline in S2 bAbs for 2FM and a sudden decline for FB1 at 27 wpc. In contrast, the intensity values of NC bAbs show a slight peak in NC bAbs at 24 wpc for FB1, 15 wpc for 2FM and 2FP, and 7 wpc for 2FL (Figure S8D). No significant difference was observed between NC band intensities of each vaccinated cat and those of LNP-control 2FL. Consequently, serum from each time point was evaluated for their reciprocal endpoint titer of NC bAbs (Figure 12C, bottom of each strip). 2FM and FB1 have the lowest endpoint titer of 10^7^ at 27 wpc, suggesting that both are clearing FIPV2 infection more rapidly than the others.

## 4. Discussions

The current two pilot studies, with minimum numbers of cats, were conducted to evaluate the safety of the pan-CoV pDNA-LNP vaccines and provide a few insights regarding their immunogenicity. We did not expect to obtain vaccine efficacy against FCoV1 coinfection in immunosuppressed FIV-infected cats (Pilot Study 1) and in a small study of four vaccinated and three control cats (Pilot Study 2), especially against high-dose, heterologous serotype-2 FIPV2. Our immediate goals were to produce pan-CoV vaccines and perform two pilot studies to determine (1) if the addition of CTL epitope sequences from FCoV/SCoV2-conserved RdRp, which is our novel concept, will enhance the immunogenicity of the B-cell epitopes and will not interfere with the analyses of challenge infection status, (2) if removing all known inflammatory regions of the S proteins, using the prefusion configuration produced by AI-based I TASSER modeling, will adequately stimulate NAb/XNAb production to RBD and T-cell cytokine responses to RBD using the LNP delivery system, and (3) whether an LNP delivery system, which is also considered to be an adjuvant [55], can be used safely as a delivery system.

We have previously determined that both COVID-19-vaccinated humans and FCoV1-infected cats possessed strong SCoV2/FCoV1-specific, CTL-promoting T-cell immunity [30]. We decided to use CTL epitopes from only RdRp (Figure S2) and not NC (Figure S3) in our pan-CoV vaccines, because the RdRp aa sequence is highly conserved among all FCoVs and does not conflict with FCoV diagnostics for infection and FCoV/FIPV-infected pet cats. Furthermore, the CTL 9-mer epitope sites are mostly identical to those between SCoV2 and FCoV1 for RdRp, with high aa similarities at appropriate CTL pockets for the HLA/FLA alleles (Figure 4A). More importantly, we excluded the CTL peptide sequences from NC in the pan-CoV vaccines to prevent the production of NC antibodies (Abs) induced by the vaccines in both pilot studies. NC proteins are highly immunogenic, and the production of NC Abs occurs rapidly upon FCoV infection [18], and possibly upon NC vaccination. Additionally, few NC Abs cross-react with the NC epitopes from both FCoV1 and FCoV2 (Figure 6B, HOL and HOK; Figure 10B, FCoV1-infected UG4) [56,57]. Thus, by excluding NC CTL epitopes from the vaccine, the presence of NC Abs upon FCoV challenge will indicate FCoV infection in the inoculated cats.

We inserted the B-cell and CTL epitope sequences individually into each pCI-H2H-*eGFP* upstream of the eGFP segment and downstream of the chicken actin promoter. We then evaluated the expression of each B-cell sequence or combined CTL epitope sequences using GFP as a marker for expression in feline Fc9 cells. This in vitro system was used to forecast the potential level of expression in cats and to assess whether expression correlated with the estimated half-life provided by the Expansy ProtParam tool. Based on the in vitro expression, the three best vaccine immunogens were SCoV2-fHR (32.2% expression), FCoV1-T9-T11 (27.5%), and FCoV1-Ori (26.2%) (Figure 5). All of these B-cell epitope constructs had a long estimated half-life of 100 h and low pI, which is ideal for expression on Goli apparatus and ER, since proteins found in these compartments generally have low pH [58,59].

In Pilot Study 1, our pan-CoV vaccine-1 was used in one FIV/FCoV1-coinfected cat and one FCoV1-cleared/FIV-infected cat as a therapeutic vaccine and to determine whether XNAbs develop in immunosuppressed FIV-infected cats. Remarkably, FCoV1/FIV-coinfected cats (HOL and HON) had XbAbs to FIPV2-WV S, S2, and NC (Figure 6B for HOL) and one of them (HON) had XbAbs to SCoV2 RBD (Figure 6C). Clearing of FCoV1 infection by single housing is demonstrated first by the loss of XbAbs to S protein followed by S2 and then NC (Figure 6B for HOK). Some chronically infected cats may not clear FCoV infection, as demonstrated by HOL’s XbAbs to FCoV2-WV (Figure 6B) and HOL’s and HON’s bAbs to FCoV1 RBD (Figure 6C, bottom). The loss of XbAbs to FIPV2-WV and bAbs to FCoV1 RBD for HOK at 6mo post-infection (Figure 6B,C, bottom) indicated that HOK cleared FCoV1 infection. The clearing of FCoV1 infection was further supported by the lack of an XNAb titer at 6mo post-infection and also by the development of XNAb upon vaccinations (Figure 7A).

In Pilot Study 1, FCoV1-based pan-CoV vaccine-1 induced low titers of XNAbs to FCoV2 in HOK and HOL which were slightly lower than those in chronically FCoV1-infected HON (Figure 7A–C). Unexpectedly, the sera from unvaccinated FCoV1/FIV-coinfected cat HON contained the strongest XNAbs to FCoV2 NAb epitope(s) (Figure 7C), whereas, as expected, the sera from unvaccinated FCoV1-cleared/FIV-infected cat HOO were generally negative for XNAbs (Figure 7D). Thus, NAb analyses for differentiating infection with FCoV1/FIPV1 from FCoV2/FIPV2 must be confirmed using bAbs to FCoV1 RBD and FCoV2 RBD. In contrast, T-cell responses were directed to FCoV1 RBD and did not cross-stimulate responses to FCoV2 RBD (Figure 8A). The IL-2/IFNγ T-cell responses to FCoV1 RBD post-first vaccination were negative for HOK, but extremely high for HOL, most likely caused by active infection and vaccine stimulation. HOK developed a significant IFNγ response to FCoV1 RBD only at post-second vaccination, whereas HOL’s responses decreased, but were still significantly high compared to her PBS control (Figure 8B). These decreases in both IFNγ/IL-2 responses to FCoV1 RBD in HOL were most likely caused by a decline in her immune system, as exemplified by the decline in both IFNγ/IL-2 T-cell responses to ConA, which were most likely enhanced by oral prednisolone to control her diarrhea. However, we did not observe any weight loss even after a second vaccination with high-dose pan-CoV vaccine 1 (Figure S4).

The major goal of Pilot Study 2 was to develop and test the SCoV2 spike B-cell epitopes minimized by deleting four major adverse regions and exchanging one SCoV2 BP motif with FCoV1 HR2. Deletions of these regions produced the SCoV2 and FCoV1 B-cell epitope structures of RBD-HR1-SH-HR2-TM-CT for FCoV1/SCoV2-based pan-CoV vaccine-1 and FCoV1-based pan-CoV vaccine-3 (Figure 9A). The pan-CoV vaccine-2 included the pDNA of the shortened RBD (FCoV1-sRBD) B-cell epitope sequence (Figure S1), together with the pDNA of the SCoV2-fHR sequence. The SCoV2-fHR protein has SCoV2 RBD and SH with FCoV1 HR1, HR2, TM, and CT to remove SCoV2 HR2, which has a BP sequence. The SCoV2-fHR configuration was the most difficult to develop because simply replacing the SCoV2 HR2 with HR2 from FCoV1, FCoV2, common cold human αCoV (CHαCoV) NL-63, or CHαCoV 229E did not adequately configure with the SCoV2 HR1. Furthermore, shortening the HR2 of FCoV1, FCoV2, NL-63, or 229E to the size of SCoV2 HR1, while retaining the aa sequence similarity/identity, did not provide a stable configuration between the shortened αCoV HR2 and SCoV2 HR1. The best results were obtained when both SCoV2 HR1 and HR2 were replaced with FCoV1 HR1 and HR2, followed by replacing SCoV2 TM-CT with FCoV1 TM-CT for vaccination of cats. Remarkably, the pDNA of such recombinant SCoV2-fHR produced the highest eGFP expression in the in vitro Fc9 cells (Figure 5B).

In Pilot Study 2, we were surprised that immunogenicity to S2 HR1-SH-HR2 construct did not increase with more vaccinations (Figure 10A) when all vaccines had the same FCoV1 S2 construct. In fact, serum from 2FM at post-last (fifth) vaccination with Pan-CoV vaccine-1 had less S2 XbAb reactivity (Figure 10A, lane 3) than the one at post-second vaccination with pan-CoV vaccine-2 (lane 2). Similarly, serum from 2FP at post-last vaccination had less S2 XbAbs than the post-fourth vaccination with pan-CoV vaccine-3 (lane 2). Serum from both 2FN and FB1 had substantial S2 reactivity at post-second vaccination which either decreased or completely lost S2 band intensity at post-fourth vaccination on high-dose immunoblot strips (Figure 10B). In our Pilot Study 2, the peak bAb titer appears at post-second and -fourth vaccinations (Figure 10B, Table S2). According to the studies, on repeated vaccination with mRNA-LNP in mice, the Abs to the mRNA immunogen after reaching the peak decreases with additional vaccinations, caused by inflammatory responses to LNP overwhelming or tolerizing the immunity to the immunogen [60].

The third goal of Pilot Study 2 with SPF cats was to determine the safety of the three pan-CoV vaccines in LNP against FIPV2-UCD2 challenge. The human COVID-19 vaccine publications did not include an LNP-immunized control group and did not state safety with annual boosting [61,62,63]. Additionally, these articles did not describe performing accelerated multiple vaccinations in pre-clinical animal studies, most likely due to the urgency of the COVID-19 crisis in 2020. Consequently, two LNP-immunized control SPF cats and one unimmunized control cat were included in our Pilot Study 2. Five vaccinations with our pan-CoV vaccines or LNP alone were tested within the vaccination period from the first vaccination to the last vaccination of approximately 33 weeks or 7.6mo. The weight loss started post-fourth vaccination for 2FL and 2FM, post-third vaccination for FB1 and 2FP, and 15.1 weeks post-second vaccination for 2FN and 2FO (Table S2). Based on HALO Image Analysis of high-dose immunoblot strips (Figure 10B), 2FM and 2FP had the most XbAbs to S2 at post-fourth vaccination, and 2FN and FB1 had the most XbAbs to S2 at post-second vaccination. Specifically, the peak S2 XbAbs preceded the start of weight loss for FB1, were concurrent to the start of weight loss for 2FN and 2FM, and occurred after the start of weight loss for 2FP (Table S2). These results indicate that the peak immunogenicity and initiation of weight loss may be causally related to the lowering of immunogenicity, as reported in repeated vaccination of mice [60,64,65,66]. As reported in murine studies, mice vaccinated with empty LNP, as well as mRNA-LNP, developed weight loss due to the use of LNP [64,65]. Such weight loss is speculated to be caused by the inflammatory responses induced by LNP or in combination with mRNA-LNP [60,64,65,66]. Overall, all vaccinated and control cats shortly after FIPV challenge had slight to severe weight losses on days 508 and 522, respectively (Figure 9B and Figure S7A), which were not observed in the three unvaccinated/unchallenged SPF cats (Table 1, Figure S7). Such a rapid weight loss after challenge is most likely caused by the high-dose FIPV2 infection [67], based on the fecal level of FIPV2 at 2.3wpc for 2FN and 8 wpc for the surviving three vaccinated cats (2FM, 2FP, FB1) and one control (2FL) (Figure 12B).

Upon necropsy, 2FO displayed pleural effusion and multiple organ damage typical of wet FIP. Similarly, the unvaccinated control 2FR with UTI developed recurrent UTI and pleural effusion by X-ray at 47 days pc. This finding was supported by a subsequent necropsy, which also showed multiple organ damage due to FIP (Figure S6). However, the necropsy of vaccinated 2FN, with a head lesion most likely from scratching, showed no internal signs of FIP, and the head and neck lesions of vasculitis (Figure S5) were negative for FCoV/FIPV NC antigen by immunochemistry and FCoV/FIPV RNA by pan-CoV PCR. However, her fecal samples collected from the GI tract were negative for FIPV by NSP14 RT-PCR but positive for FCoV/FIPV by RdRp RT-snPCR and the RdRp specificity of the amplified sample was confirmed by sequencing (Figure 12B). Her serum collected on the day of euthanasia at 2.3 wpc had bAb(s) to FCoV2 NC and RBD and FCoV2/FIPV2 NAb dilution titer of 10^3.2^ (Figure 10A, 2FN lane 4; Figure 10C, 2FN). The presence of bAbs to FCoV2 NC/RBD, high post-challenge NAbs to FCoV2, and FCoV RdRp RT-snPCR at 2.3 wpc clearly demonstrated that 2FN was infected with FIPV2. This observation was expected because immunity against infection is as sensitive as RdRp RT-snPCR for viruses in pleural fluid and feces. Meli et al. reported that high FCoV antibody titers correlate with high FCoV loads, and also Felten et al. reported FCoV antibody titer correlates with fecal shedding frequency [68,69]. Importantly, vasculitis lesions caused by type-III hypersensitivity are often the outcome of FIP infection [70,71,72].

As expected, 2FN was infected with the challenge virus because she had only weak vaccine-induced XbAbs to FCoV2 S2 present in the high-dose FCoV2-WV immunoblot strips (Figure 10B), but no XbAbs detected in the low-dose FCoV2-WV immunoblot strips (Figure 10A). In addition, IL2/IFNγ T-cell responses of 2FN to ConA were low, when compared to those of cats at pre-challenge, even though she had statistically significant IFNγ responses to FCoV1 RBD (Figure 11A). In contrast, vaccinated cats 2FM, 2FP, and FB1 had S2 XbAbs as early as post-fourth vaccination with pan-CoV vaccine-2 using the high-dose immunoblot strips (Figure 10B) and also with the low-dose immunoblot strips for 2FM (Figure 10A). These three vaccinated cats developed vaccine-induced XbAbs to S 160 kDa band, but only 2FM and 2FP also developed XbAbs to S 240 kDa band (Figure 10B). Conversely, when compared to their PBS-treated cultures, FB1 developed statistically significant IL-2 and IFNγ T-cell responses to the FCoV1 RBD before challenge. This was followed by 2FM with substantial increases in both IL-2/IFNγ responses to FCoV1 RBD, and then 2FP with only significant IFNγ responses to FCoV1 RBD (Figure 11A). Remarkably, vaccinated FB1 retained statistically significant IL2/IFNγ responses to FCoV1 RBD even at 3 wpc (Figure 10B). Thus, vaccinated cat 2FP had the highest XbAbs to S proteins, whereas vaccinated cat FB1 had the most consistent and durable IL2/IFNγ T-cell responses to FCoV1 RBD. However, FB1 had only low consistent XNAbs to FCoV2 at pre-challenge and the highest FCoV2 NAbs of 10^4.3^ at 3 wpc (Figure 10A, XNAb titers).

In Pilot Study 2, one of four vaccinated cats and two of three control cats were humanely euthanized due to FIP disease. However, all cats became infected with high-dose FIPV2 challenge. No statistical differences were observed in the death rate (*p* = 0.4857) and disease (weight loss) rate (*p* = 0.1429) between the vaccinated group and LNP-control group in Pilot Study 2. These results suggest, due to the limited number of cats used, that the FCoV1/SCoV2-based pan-CoV vaccine may be able to delay FIPV infection and subsequent FIP disease but needs to be confirmed by another study. A potential delay in fecal load of 2FM and 2FP was demonstrated by their fecal FIPV2 load being none-to-low in cycle 1 (RT-snPCR) and at 8 wpc when compared to a higher fecal load of LNP-control 2FL (Figure 12A, cycle 1 at 8 wpc). They also had a delay in manifestation of typical FIP signs of anemia, low albumin (A), high globulin (G), low A/G ratio, and high total protein (Table S3) [6,67]. Additionally, all live cats initiated their FIPV2 clearance at 23 wpc, except for 2FP (cycle 2 at 23 wpc), which cleared FIPV2 load in both cycles at 26 wpc (Figure 12A, 26 wpc). The duplicate immunoblot tests support the clearance of S2 bAbs in 2FM and FB1, who also have the lowest NC bAbs (Figure 12B). More importantly, no enhancement in infection or clinical disease was observed in the vaccinated cats as previously reported for FCoV [73]. FCoV/SCoV2-specific CTL activities may have contributed to a delay in FIPV2 infection and FIP disease manifestation. FIV multi-antigenic peptide vaccine, containing only FIV- and human immunodeficiency virus (HIV)-specific CTL epitopes selected by the same approach as current studies, protected vaccinated cats against low-dose heterologous FIV challenge [30,74]. In support of our CTL-selection system, the commercial Fel-O-Vax^®^ FIV also had strong FIV-specific CTL activities to FIV/HIV-specific polymerase [45]. Since FCoV1 infections are more common in the U.S. and the world, the next challenge should be if FCoV1-based pan-CoV vaccine-4 is to be used. Based on our in vitro expression analysis, our pan-CoV vaccine-4 should contain FCoV1-Ori, SCoV2-fHR, and FCoV-T9-T11, which provided the highest in vitro pDNA expression. Our overarching goal for feline pan-CoV vaccine is to combine FCoV1, FCoV2, and SCoV2 B-cell constructs, in addition to the FCoV/SCoV2-based CTL/TH epitope chains, to generate sterilizing immunity against FIPV1, FIPV2, and SCoV2.

## 5. Conclusions

The FCoV1/SCoV2-based B-cell epitope constructs, configured by AI-based I TASSER modeling program, induced bAbs to FCoV1 and SCoV2 RBDs and XNAbs to FIPV2 in Pilot Study 1. The pDNAs of B-cell constructs and CTL/TH epitope chains delivered in LNP had no adverse effects at 50 μg dose but developed adverse effects at 100 μg dose. In Pilot Study 2, three pan-CoV pDNA-LNP vaccines, each with a different FCoV1-based B-cell construct, vaccinated sequentially up to a total of five vaccinations, induced substantial levels of XbAbs and low levels of XNAbs to FIPV2, which peaked at post-second and -fourth vaccinations. Weight loss, caused most likely by LNP, initiated at weeks post-second vaccination to post-fourth vaccination. Thus, the loss of XbAbs at post-fifth vaccination may be causally related to the weight loss. Hence, a potential delay in FIPV2 infection, FIP disease manifestation, and fecal shedding was observed in the FCoV1/SCoV2-based pan-CoV pDNA-LNP-vaccinated live cats. Furthermore, single housing for 23–26 wpc demonstrated initiation of FIPV2 clearance in live vaccinated and LNP-control cats.

## Figures and Tables

**Table 1:** Weight loss (Wt) as an indicator of the disease condition of FIPV2 infection.

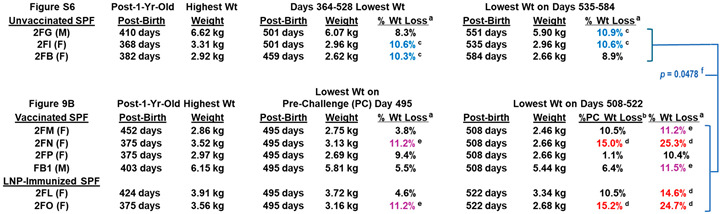

^a^ Percent weight loss (% Wt Loss) is the weight at the designated day when compared to its highest weight at post one-year-old (Post-1-Yr-Oid) shown in Figure 9B and Figure S6. ^b^ Percent pre-challenge weight loss (% PC Wt Loss) is the weight at the designated day when compared to its weight at pre-challenge day 495. ^c^ The highest % weight loss is shown in blue percentage for unvaccinated SPF cats. ^d^ The highest % weight loss and % PC weight loss, ≥3.7% above those of unvaccinated cats, are shown in red percentage for vaccinated and LNP-immunized cats. ^e^ The % weight loss with marginal level (≥0.3%), compared to those of unvaccinated cats, are shown in lavender percentage for vaccinated and LNP-immunized cats. ^f^ The highest % weight loss of three unvaccinated SPF cats was compared to the highest % weight loss of all six challenged cats, to determine whether statistically significant difference exists when >11% weight loss is caused by FIPV2 infection. Thus, significant difference (*p* = 0.0478) exists between these two groups.

## Data Availability

The complete amino acid (aa) and nucleotide (nt) sequences of FIPV2-UCD2 (accession number: PV941793) have been deposited to the NCBI GenBank. We have included the following nt and aa sequences of the B-cell constructs also to the NCBI GenBank for the following SCoV2-fHR (PV982376); FCoV1-sRBD (PV982374); FCoV1-UCD1-Ori (PV982373); SCoV2-Wuhan-Ori (PV982375). The CTL/TH aa sequences of SCoV2 and FCoV1 RdRp proteins for feline pan-CoV vaccine are shown in Figure 4 and those for FCoV1 and FCoV2 proteins are shown in Figure S2.

## Supplementary Materials

The following supporting information can be downloaded at: https://www.mdpi.com/article/10.3390/vaccines13111172/s1, Original Immunoblot Strips & Gels. Figure S1: Amino acid (aa) sequence alignment of FCoV1 UCD1 with FCoV2 UCD2; Figure S2: Mapping of 9-mer CTL and 15mer TH epitopes on FCoV1 and FCoV2 RdRp sequences; Figure S3: Mapping of 9-mer CTL epitopes on FCoV2 nucleocapsid (NC) for FCoV2-CTL35 chain; Figure S4: Comparing weight changes between the vaccinated FIV-infected cats and unvaccinated FIV-infected cats in Pilot Study 1. Figure S5: Skin lesions of an FIPV2-challenged/vaccinated cat 2FN. Figure S6: FIP pathology of unimmunized control cat 2FR; Figure S7: Comparison of weight changes between the cats in Pilot Study 2 and their lineage-related unvaccinated/unchallenged SPF cats. Figure S8: NSP14 RT-PCR, RdRp RT-snPCR, and FIPV2 NC immunoblot analyses. Table S1: Laboratory cats used in Pilot Studies 1 and 2. Table S2: Comparison of weight loss and peak XbAbs to S2; Table S3: Complete blood count (CBC), blood chemistry, FIPV2 load, and clinical status of live FIPV2-challenged, vaccinated and LNP-immunized cats.

## References

[1] Nooruzzaman M., Diel D.G. (2023). Infection dynamics, pathogenesis, and immunity to SARS-CoV-2 in naturally susceptible animal species. J. Immunol..

[2] Sweet A.N., André N.M., Stout A.E., Licitra B.N., Whittaker G.R. (2022). Clinical and molecular relationships between COVID-19 and feline infectious peritonitis (FIP). Viruses.

[3] Hosie M.J., Hofmann-Lehmann R., Hartmann K., Egberink H., Truyen U., Addie D.D., Belák S., Boucraut-Baralon C., Frymus T., Lloret A. (2021). Anthropogenic infection of cats during the 2020 COVID-19 pandemic. Viruses.

[4] Park E.S., Kuroda Y., Uda A., Kaku Y., Okutani A., Hotta A., Tatemoto K., Ishijima K., Inoue Y., Harada M. (2024). The comparison of pathogenicity among SARS-CoV-2 variants in domestic cats. Sci. Rep..

[5] Pedersen N.C. (2014). An update on feline infectious peritonitis: Virology and immunopathogenesis. Vet. J..

[6] Hartmann K. (2005). Feline infectious peritonitis. Vet. Clin. N. Am. Small Anim. Pract..

[7] Zuzzi-Krebitz A.M., Buchta K., Bergmann M., Krentz D., Zwicklbauer K., Dorsch R., Wess G., Fischer A., Matiasek K., Hönl A. (2024). Short treatment of 42 days with oral GS-441524 results in equal efficacy as the recommended 84-day treatment in cats suffering from feline infectious peritonitis with effusion-a prospective randomized controlled study. Viruses.

[8] Murphy B.G., Perron M., Murakami E., Bauer K., Park Y., Eckstrand C., Liepnieks M., Pedersen N.C. (2018). The nucleoside analog GS-441524 strongly inhibits feline infectious peritonitis (FIP) virus in tissue culture and experimental cat infection studies. Vet. Microbiol..

[9] Taylor S.S., Coggins S., Barker E.N., Gunn-Moore D., Jeevaratnam K., Norris J.M., Hughes D., Stacey E., MacFarlane L., O’Brien C. (2023). Retrospective study and outcome of 307 cats with feline infectious peritonitis treated with legally sourced veterinary compounded preparations of remdesivir and GS-441524 (2020–2022). J. Feline Med. Surg..

[10] Scherk M.A., Ford R.B., Gaskell R.M., Hartmann K., Hurley K.F., Lappin M.R., Levy J.K., Little S.E., Nordone S.K., Sparkes A.H. (2013). 2013 AAFP Feline Vaccination Advisory Panel Report. J. Feline Med. Surg..

[11] Stone A.E.A., Brummet G.O., Carozza E.M., Kass P.H., Petersen E.P., Sykes J., Westman M.E. 2020 AAHA/AAFP Feline Vaccination Guidelines. Not Recommended Vaccines. JAAHA. https://www.aaha.org/aaha-guidelines/2020-aahaaafp-feline-vaccination-guidelines/feline-vaccination-home/.

[12] O’Brian A., Mettelman R.C., Volk A., Andre N.M., Whittaker G.R., Baker S.C. (2018). Characterizing replication kinetics and plaque production of type I feline infectious virus in three feline cell lines. Virology.

[13] Thiel V., Thiel H.-J., Tekes G. (2014). Tackling feline infectious peritonitis via reverse genetics. Bioengineered.

[14] Addie D.D., Jarrett J.O. (2001). Use of a reverse-transcriptase polymerase chain reaction for monitoring feline coronavirus shedding by healthy cats. Vet. Rec..

[15] Drechsler Y., Alcaraz A., Bossong F.J., Collisson E.W., Diniz P.P.V.P. (2011). Feline coronavirus in multicat environments. Vet. Clin. N. Am. Small Anim. Pract..

[16] Barrs V.R., Peiris M., Tam K.W.S., Law P.Y.T., Brackman C.J., To E.M.W., Yu V.Y.T., Chu D.K.W., Perera R.A.P.M., Sit T.H.C. (2020). SARS-CoV-2 in quarantined domestic cats from COVID-19 households or close contacts, Hong Kong, China. Emerg. Infect. Dis..

[17] Klaus J., Meli M.L., Willi B., Nadeau S., Beisel C., Stadler T., Egberink H., Zhao S., Lutz H., SARS-CoV-Sequencing Team (2021). Detection and genome sequencing of SARS-CoV-2 in a domestic cat with respiratory signs in Switzerland. Viruses.

[18] Yamamoto J.K., Edison L.K., Rowe-Haas D.K., Takano T., Gilor C., Crews C.D., Tuanyok A., Arukha A.P., Shiomitsu S., Walden H.D.S. (2023). Both feline coronavirus serotypes 1 and 2 infected domestic cats develop cross-reactive antibodies to SARS-CoV-2 receptor binding domain: Its implication to pan-CoV vaccine development. Viruses.

[19] Singh A.K., Goel K., Dhanawat M. (2025). Plasmid DNA and mRNA delivery: Approaches and challenges. Adv. Immunol..

[20] Starr T.N., Czudnochowski N., Liu Z., Zatta F., Park Y.J., Addetia A., Pinto D., Beltramello M., Hernandez P., Greaney A.J. (2021). SARS-CoV-2 RBD antibodies that maximize breadth and resistance to escape. Nature.

[21] Cheng M.H., Zhang S., Porritt R.A., Rivas M.N., Paschold L., Wilscher E., Binder M., Arditi M., Bahar I. (2020). Superantigenic character of an insert unique to SARS-CoV-2 spike supported by skewed TCR repertoire in patients with hyperinflammation. Proc. Natl. Acad. Sci. USA.

[22] Tu T.H., Bennani F.E., Masroori N., Liu C., Nemati A., Rozza N., Grunbaum A.M., Kremer R., Milhalcioiu C., Roy D.C. (2025). The identification of a SARs-CoV2 S2 protein derived peptide with super-antigen-like stimulatory properties on T-cells. Commun. Biol..

[23] Ma X., Zou F., Yu F., Li R., Yuan Y., Zhang Y., Zhang X., Deng J., Chen T., Song Z. (2020). Nanoparticle vaccines based on the receptor binding domain (RBD) and heptad repeat (HR) of SARS-CoV-2 elicit robust protective immune responses. Immunity.

[24] He C., Yang J., Hong W., Chen Z., Peng D., Lei H., Alu A., He X., Bi Z., Jiang X. (2022). A self-assembled trimeric protein vaccine induces protective immunity against Omicron variant. Nat. Commun..

[25] Mulligan M.J., Lyke K.E., Kitchin N., Absalon J., Gurtman A., Lockhart S., Neuzil K., Raabe V., Bailey R., Swanson K.A. (2020). Phase I/II study of COVID-19 RNA vaccine BNT162b1 in adults. Nature.

[26] Vogel A.B., Kanevsky I., Che Y., Swanson K.A., Muik A., Vormehr M., Kranz L.M., Walzer K.C., Hein S., Güler A. (2021). BNT162b vaccines protect rhesus macaques from SARS-CoV-2. Nature.

[27] Liu Y., Ye Q. (2023). The key site variation and immune challenges in SARS-CoV-2 evolution. Vaccines.

[28] (2025). HALO Image Analysis Platform.

[29] Nair S., Sahay B., Arukha A.P., Edison L.K., Crews C.D., Morris J.G., Kariyawasam S., Yamamoto J.K. (2024). Interferon-γ/IL-2 and mRNA responses to the SARS-CoV2, feline coronavirus serotypes 1 (FCoV1), and FCoV2 receptor binding domains by the T cells from COVID-19-vaccinated humans and FCoV1-infected cats. Methods Mol. Biol..

[30] Sahay B., Aranyos A.M., McAvoy A., Yamamoto J.K. (2018). Utilization of feline ELISpot to evaluate the immunogenicity of a T cell-based FIV MAP vaccine. Methods Mol. Biol..

[31] I-TASSER Server for Protein Structure & Function Predictions (Zhang Lab, University of Michigan, Ann Arbor, MI and National University of Singapore, Kent Ridge, Singapore). https://zhanggroup.org/I-TASSER/.

[32] Schrodinger LLC PyMOL 2.5 and 3.0.3 Systems. https://pymol.org/2/.

[33] Pinto D., Sauer M.M., Czudnochowski N., Low J.S., Tortorici M.A., Housley M.P., Noack J., Walls A.C., Bowen J.E., Guarino B. (2021). Broad betacoronavirus neutralization by a stem helix-specific human antibody. Science.

[34] Expasy ProtParam Tool. https://web.expasy.org/protparam/.

[35] Kida K., Hohdatsu T., Fujii K., Koyama H. (1999). Selection of antigenic variants of the S glycoprotein of feline infectious peritonitis virus and analysis of antigenic sites involved in neutralization. J. Vet. Med. Sci..

[36] Corapi W.V., Darteil R.J., Audonnet J.C., Chappuis G.E. (1995). Localization of antigenic sites of the S glycoprotein of feline infectious peritonitis virus involved in neutralization and antibody-dependent enhancement. J. Virol..

[37] DTU Health, Tech Department of Health Technology, Lyngby, Denmark, NetMHCpan 4.1 Server. https://services.healthtech.dtu.dk/services/NetMHCpan-4.1/.

[38] DTU Health, Tech Department of Health Technology, Lyngby, Denmark, NetCTL 1.2 Server. https://services.healthtech.dtu.dk/services/NetCTL-1.2/.

[39] DTU Health, Tech Department of Health Technology, Lyngby, Denmark, NetMHCIIpan 4.0 Server. https://services.healthtech.dtu.dk/services/NetMHCIIpan-4.0/.

[40] DTU Health, Tech Department of Health Technology, Lyngby, Denmark, NetMHCII 2.3 Server. https://services.healthtech.dtu.dk/services/NetMHCII-2.3/.

[41] JustBio Server, AgileBio LLC, San Diego, CA. https://justbio.com/.

[42] Marsh S.G.E., Parham P., Barber L.D. (2000). The HLA Facts Book.

[43] Allele Frequencies in Worldwide Populations Database Provides Top 2-3 HLA Alleles Under Population of USA Caucasian and USA African American in Their Surveys. http://www.allelefrequencies.net/hla6006a.asp.

[44] National Marrow Donor Program: High-Resolution HLA Alleles and Haplotypes in the US Populations as of 2006. https://network.nmdp.org/services-support/bioinformatics-immunobiology/haplotype-frequencies/high-resolution-hla-alleles-and-haplotypes-in-the-us-populations-as-of-2006.

[45] Aranyos A.M., Roff S.R., Pu R., Owen J.L., Coleman J.K., Yamamoto J.K. (2016). An initial examination of the potential role of T-cell immunity in protection against feline immunodeficiency virus (FIV) infection. Vaccine.

[46] Takara Xfect Transfectiton Reagent Protocol-At-A-Glance (PT5003-2, Takara Bio USA, Inc. San Jose, CA). https://www.takarabio.com/documents/User%20Manual/Xfect%20Transfection%20Reagent%20Protocol/Xfect%20Transfection%20Reagent%20Protocol-At-A-Glance_103012.pdf.

[47] Tanaka Y., Sasaki T., Matsuda R., Uematsu Y., Yamaguchi T. (2015). Molecular epidemiological study of feline coronavirus strains in Japan using RT-PCR targeting nsp14 gene. BMC Vet. Res..

[48] Watanabe S., Masangkay J.S., Nagata N., Morikawa S., Mizutani T., Fukushi S., Alviola P., Omatsu T., Ueda N., Iha K. (2010). Bat coronaviruses and experimental infection of bats, the Philippines. Emerg. Infect. Dis..

[49] Holbrook M.G., Anthony S.J., Navarrele-Macias I., Bestebroer T., Muster V.J., van Doremalen N. (2021). Updated and validated pan-coronavirus PCR assay to detect all coronavirus genera. Viruses.

[50] Steiner S., Kratzel A., Barut G.T., Lang R.M., Moreira E.A., Thomann L., Kelly J.N., Thiel V. (2024). Sars-CoV-2 biology and host interactions. Nat. Rev. Microbiol..

[51] Hofmann H., Pyrc K., van der Hoek L., Geier M., Berkhout B., Pöhlmann S. (2005). Human coronavirus NL63 employs the severe acute respiratory syndrome coronavirus receptor for cellular entry. Proc. Natl. Acad. Sci. USA.

[52] Wu K., Li W., Peng G., Li F. (2009). Crystal structure of NL63 respiratory coronavirus receptor-binding domain complexed with its human receptor. Proc. Natl. Acad. Sci. USA.

[53] Lützner N., Kalbacher H. (2008). Quantifying cathepsin S activity in antigen presenting cells using a novel specific substrate. J. Biol. Chem..

[54] Huang C., Conlee D., Gill M., Chu H.J. (2010). Dual-subtype feline immunodeficiency virus vaccine provides 12 months of protective immunity against heterologous challenge. J. Feline Med. Surg..

[55] Chatzikleanthous D., O’Hagan D.T., Adamo R. (2021). Lipid-based nanoparticles for delivery of vaccine adjuvants and antigens: Toward multicomponent vaccines. Mol. Pharm..

[56] Vlasova A.N., Zhang X., Hasoksuz M., Nagesha H.S., Haynes L.M., Fang Y., Lu S., Saif L.J. (2007). Two-way antigenic cross-reactivity between severe acute respiratory syndrome coronavirus (SARS-CoV) and Group 1 animal CoVs is mediated through an antigenic site in the N-terminal region of the SARS-CoV nucleoprotein. J. Virol..

[57] Zhao S., Li W., Schuurman N., van Kuppeveld F., Bosch B.J., Egberink H. (2019). Serological Screening for coronavirus infections in cats. Viruses.

[58] Tokmakov A.A., Kurotani A., Sato K.I. (2021). Protein pI and intracellular localization. Front. Mol. Biosci..

[59] Kurotani A., Tokmakov A.A., Sato K.I., Stefanov V.E., Yamada Y., Sakurai T. (2019). Localization-specific distributions of protein pI in human proteome are governed by local pH and membrane charge. BMC Mol. Cell Biol..

[60] Ndeupen S., Qin Z., Jacobsen S., Bouteau A., Estanbouli H., Igyártó B.Z. (2021). The mRNA-LNP platform’s lipid nanoparticle component used in preclinical vaccine studies is highly inflammatory. iScience.

[61] Jackson L.A., Anderson E.J., Rouphael N.G., Roberts P.C., Makhene M., Coler R.N., McCullough M.P., Chappell J.D., Denison M.R., Stevens L.J. (2020). An mRNA vaccine against SARS-CoV-2—Preliminary report. N. Engl. J. Med..

[62] Walsh E.E., Frenck R.W., Falsey A.R., Kitchin N., Absalon J., Gurtman A., Lockhart S., Neuzil K., Mulligan M.J., Bailey R. (2020). Safety and immunogenicity of two RNA-based COVID-19 vaccine candidates. N. Engl. J. Med..

[63] Corbett K.S., Flynn B., Foulds K.E., Francica J.R., Boyoglu-Barnum S., Werner A.P., Flach B., O’Connell S., Bock K.W., Minai M. (2020). Evaluation of the mRNA-1273 vaccine against SARS-CoV-2 in nonhuman primates. N. Engl. J. Med..

[64] Gao F.-X., Wu R.-X., Shen M.-Y., Huang J.-J., Li T.T., Hu C., Luo F.-Y., Song S.-Y., Mu S., Hao Y.-N. (2022). Extended SARS-CoV-2 RBD booster vaccination induces humoral and cellular immune tolerance in mice. iScience.

[65] Wang J., Ding Y., Chong K., Cui M., Cao Z., Tang C., Tian Z., Hu Y., Zhao Y., Jiang S. (2024). Recent advances in lipid nanoparticles and their safety concerns for mRNA delivery. Vaccines.

[66] Tahtinen S., Tong A.-J., Himmels P., Oh J., Paler-Martinez A., Kim L., Wichner S., Oei Y., McCarron M.J., Freund E.C. (2022). IL-1 and IL-1ra are key regulators of the inflammatory response to RNA vaccines. Nat. Immunol..

[67] Sherding R.G., Birchard S.J., Sherding R.G. (2006). Feline infectious peritonitis (*Feline coronavirus*). Saunders Manual of Small Animal Practice.

[68] Meli M., Kipar A., Muller C., Jenal K., Gonczi E., Borel N., Gunn-Moore D., Chalmers S., Lin F., Reinacher M. (2004). High viral loads despite absence of clinical and pathological findings in cats experimentally infected with feline coronavirus (FCoV) type I and in naturally FCoV-infected cats. J. Feline Med. Surg..

[69] Felten S., Klein-Richers U., Hofmann-Lehmann R., Bergmann M., Unterer S., Leutenegger C.M., Hartmann K. (2020). Correlation of feline coronavirus shedding in feces with coronavirus antibody titer. Pathogens.

[70] Stranieri A., Scavone D., Paltrinieri S., Giordano A., Bonsembiante F., Ferro S., Gelain M.E., Meazzi S., Lauzi S. (2020). Concordance between histology, immunohistochemistry, and RT-PCR in the diagnosis of feline infectious peritonitis. Pathogens.

[71] Mônica Slaviero M., Cony F.G., da Silva R.C., De Lorenzo C., de Almeida B.A., Bertolini M., Driemeier D., Pavarini S.P., Sonne L. (2024). Pathological findings and patterns of feline infectious peritonitis in the respiratory tract of cats. J. Comp. Pathol..

[72] Paltrinieri S., Parodi M.C., Cammarata G. (1999). In vivo diagnosis of feline infectious peritonitis by comparison of protein content, cytology, and direct immunofluorescence test on peritoneal and pleural effusions. J. Vet. Diagn. Investig..

[73] Takano T., Yamada S., Doki T., Hohdatsu T. (2019). Pathogenesis of oral type I feline infectious peritonitis virus (FIPV) infection: Antibody dependent enhancement infection of cats with type I FIPV via the oral route. J. Vet. Med. Sci..

[74] Sahay B., Aranyos A.M., Mishra M., McAvoy A.C., Martin M.M., Pu R., Shiomitsu S., Shiomitsu K., Dark M.J., Sanou M.P. (2019). Immunogenicity and efficacy of a novel multi-antigenic peptide vaccine based on cross-reactivity between feline and human immunodeficiency viruses. Viruses.

