# Supplementary material for: Pilot Studies Testing Novel Minimized Pan-Coronavirus (CoV) Vaccines in Feline Immunodeficiency Virus-Infected Cats With or Without Feline CoV Serotype-1 (FCoV1) Coinfection and in Specific-Pathogen-Free Cats Against Pathogenic FCoV2
Source: Vaccines (Basel). 2025 Nov 18;13(11):1172. doi: 10.3390/vaccines13111172 (PMC12656953; doi:10.3390/vaccines13111172)
Supplement: Supplementary file 1 [file vaccines-13-01172-s001.zip › vaccines-3790918-supplementary/Original Immunoblot Strips & Gels.pdf]

Table of Content

HALO Quantitative Image Analysis for Immunoblot Strips:

PPT Slide 2: Immunoblot Figure 6B

PPT Slide 3: Immunoblot Figure 6C

PPT Slide 4: Immunoblot Figure 10A

PPT Slide 5: Immunoblot Figure 10B

PPT Slide 6: Immunoblot Figure 10C

PPT Slide 7: Immunoblot Figure 12C Duplicate Set 1 and Figure S8C Duplicate Set 2

HALO Quantitative Image Analysis for RT-PCR Gels:

PPT Slide 8: RdRp semi-nested RT-snPCR (RT-snPCR) Figure 12A in Manuscript (cropped gels Set 1)

PPT Slide 9: RdRp RT-snPCR Gels Set 1 at 8WPC & 21WPC for Figure 12A Left top & bottom (whole gels)

PPT Slide 10: RdRp RT-snPCR Gels Set 1 at 23WPC & 26WPC for Figure 12A Right top & bottom (whole gels)

PPT Slide 11: RdRp RT-snPCR Gels on Duplicate Set 2 at 8WPC & 21WPC together with description (cropped gels)

PPT Slide 12: RdRp RT-snPCR Gels on Duplicate Set 2 at 8WPC for Bar Graph Figures 12B/S8B (whole gels)

PPT Slide 13: RdRp RT-snPCR Gels on Duplicate Set 2 at 21WPC for Bar Graph Figures 12B/S8B (whole gels)

PPT Slide 14: RdRp RT-snPCR Gels on Duplicate Set 2 at 23WPC & 26WPC for Bar Graph Figures 12B/S8B (whole gels)

PPT Slide 15: NSP14 regular RT-PCR for Supplemental Figure S8A with Cycle-1 Gel at 8WPC (top cropped, bottom whole gel)

Figure 6B

Figures 6B in text has brightness intensity of 8% and contrast intensity of 0%.

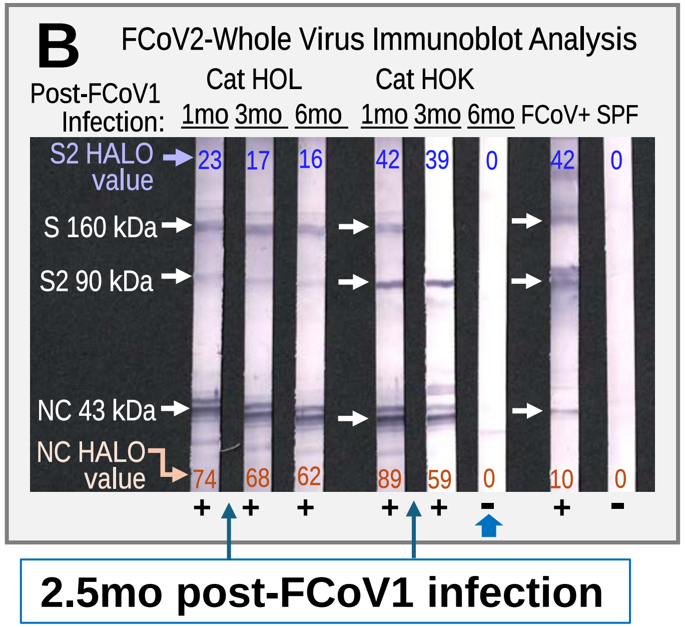

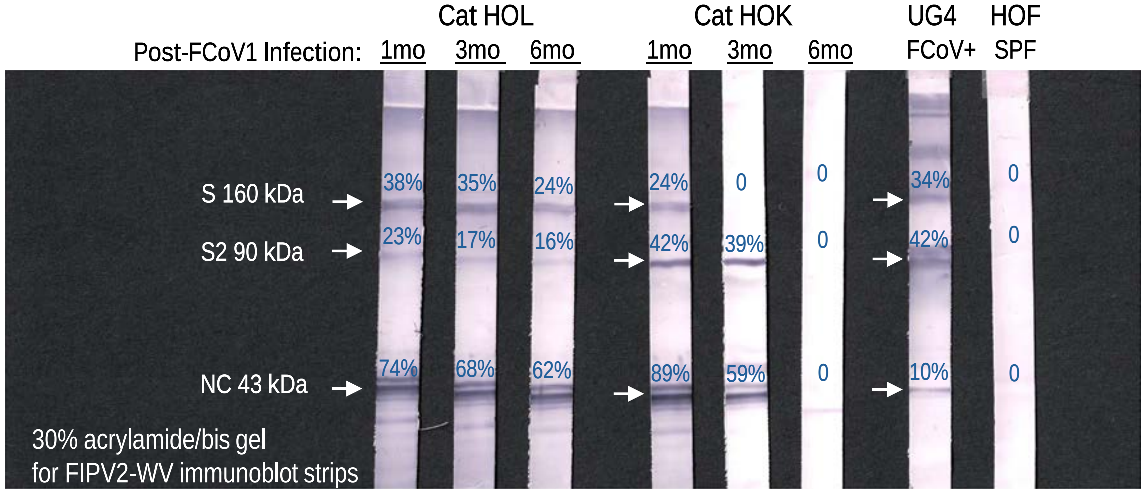

HALO values should be in percentage.

Figure 6C

This figure 6C in the text has brightness intensity of 5% and contrast intensity of 5%.

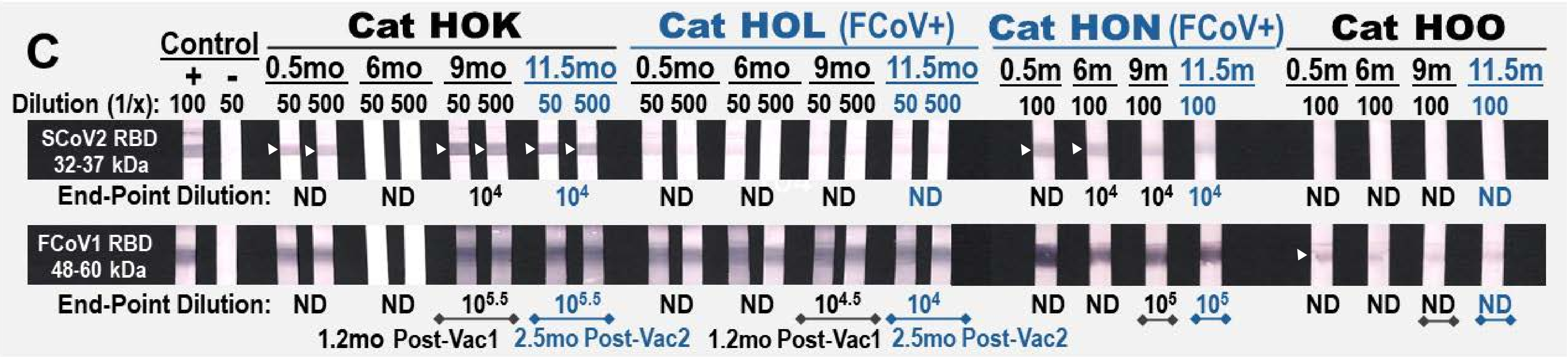

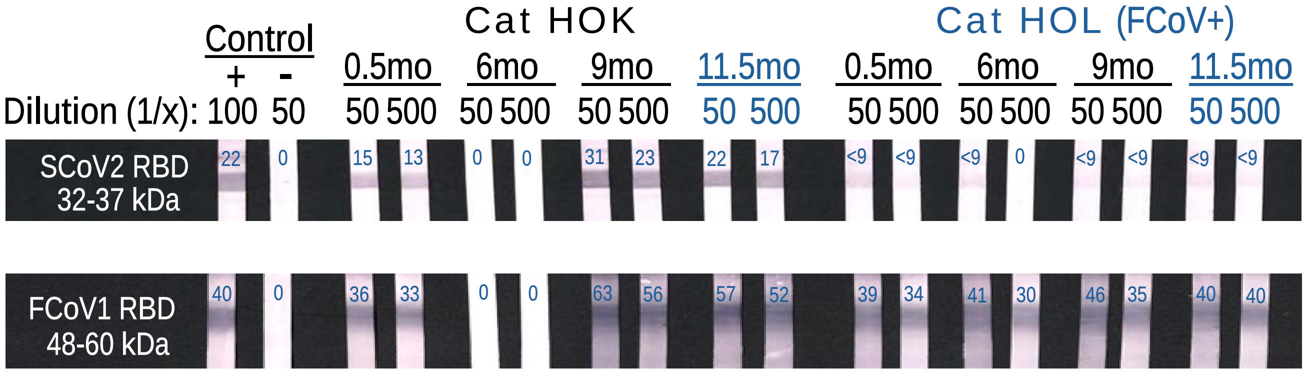

Percent band brightness intensity adjustment of -5% at 5% contrast intensity

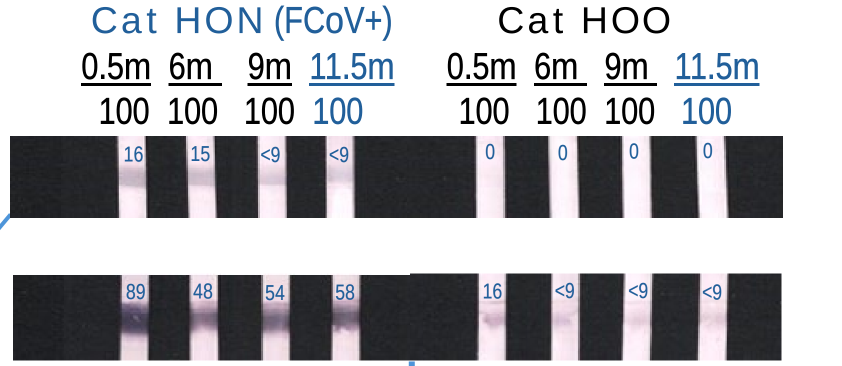

Percent band intensity adjustment of -5% at 5% contrast intensity

Percent band intensity adjustment of -5% at 5% contrast intensity

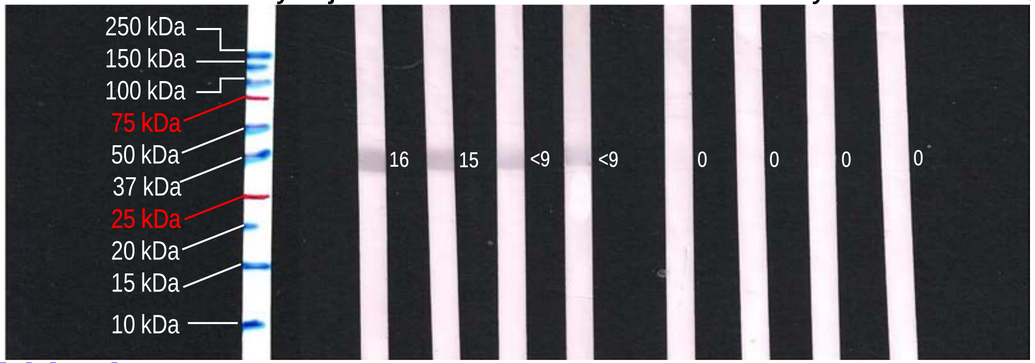

Percent band intensity adjustment of -5% at 5% contrast intensity

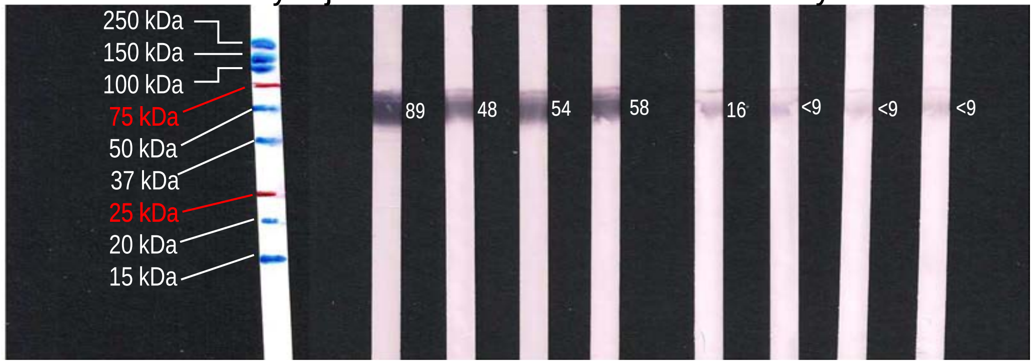

40% acrylamide/bis gel for RBD immunoblot strips

HALO values should be in percentage.

Figure 10A

Figure 10A in the text has no adjustments for brightness and contrast intensities.

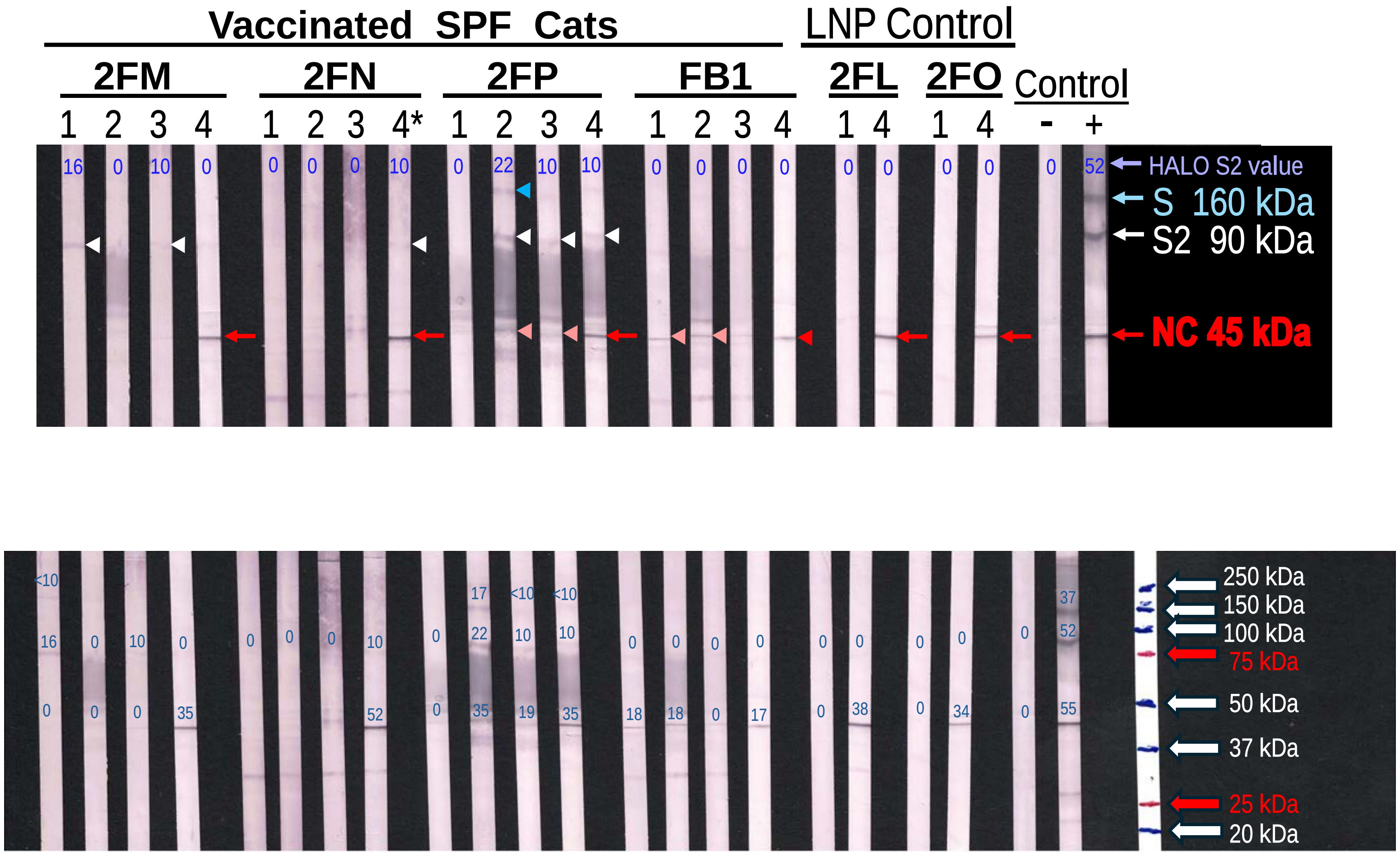

30% acrylamide/bis gel
for FIPV2-WV immunoblot strips

HALO values should be in percentage.

Figure 10B

Figure 10B in the text has no adjustments for brightness and contrast intensities.

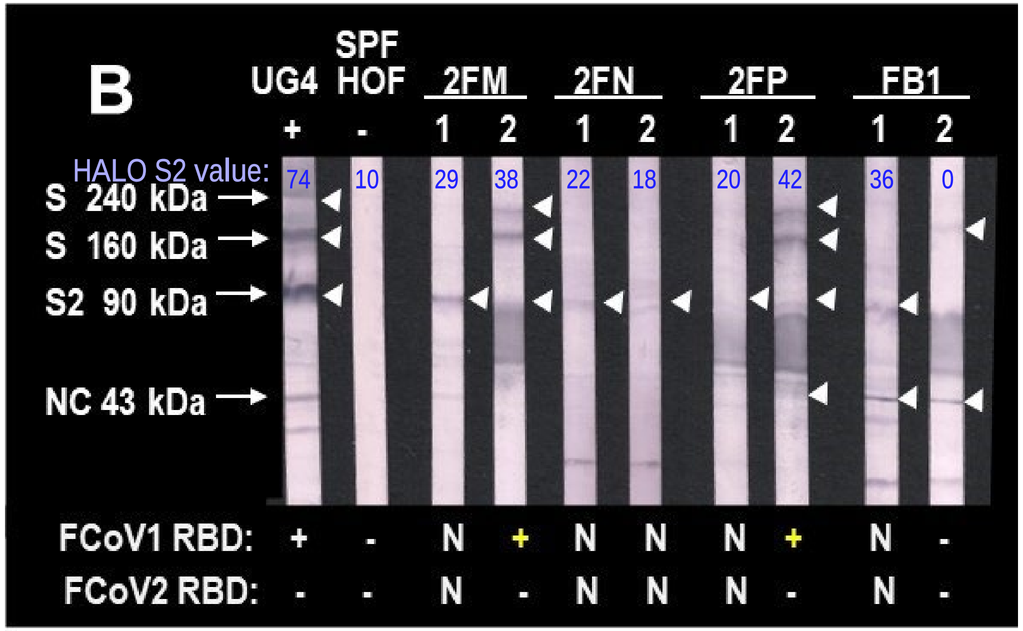

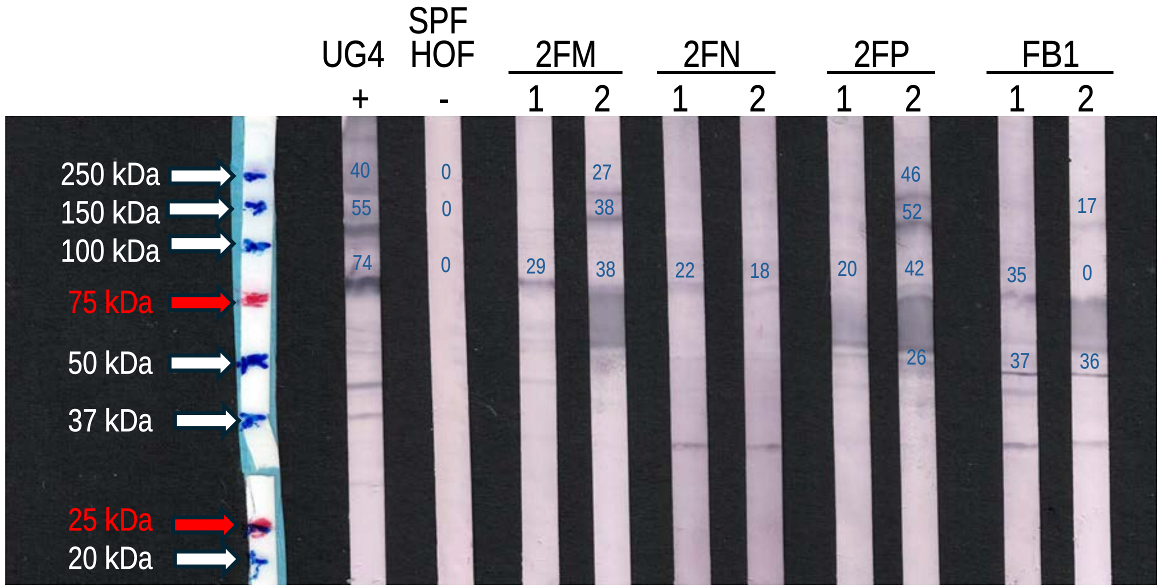

30% acrylamide/bis gel
for FIPV2-WV immunoblot strips

HALO values should be in percentage.

Figure 10C

Figure 10C in the text has no adjustments made for both brightness and contrast intensities.

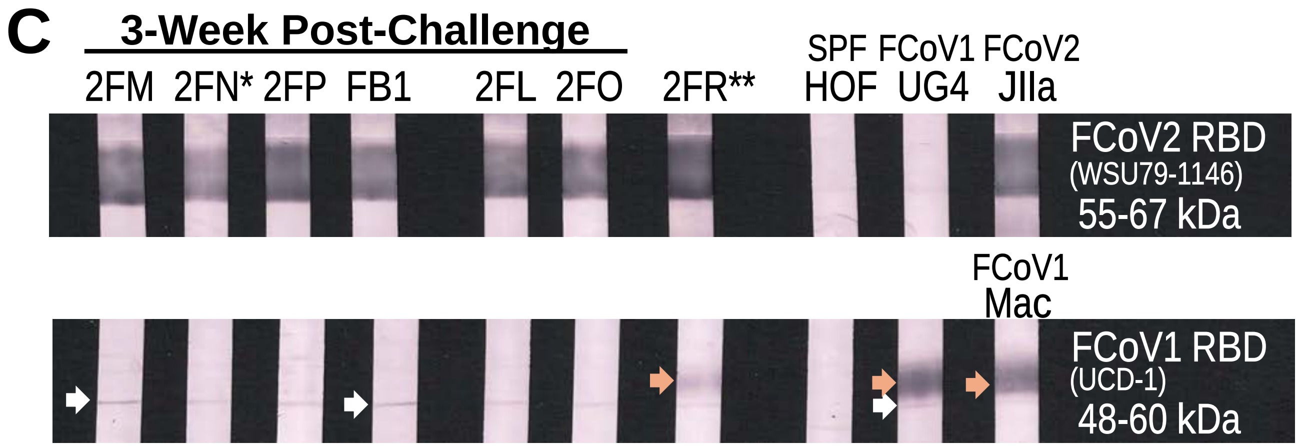

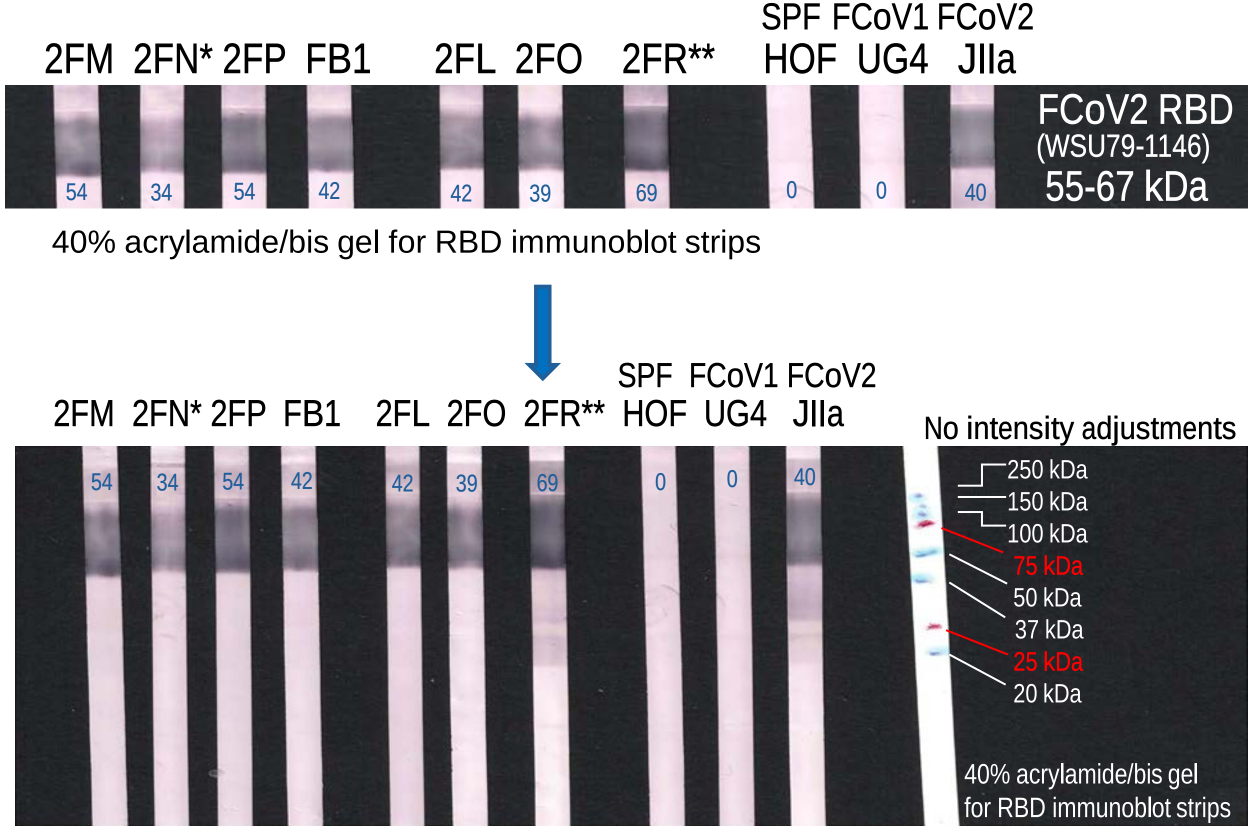

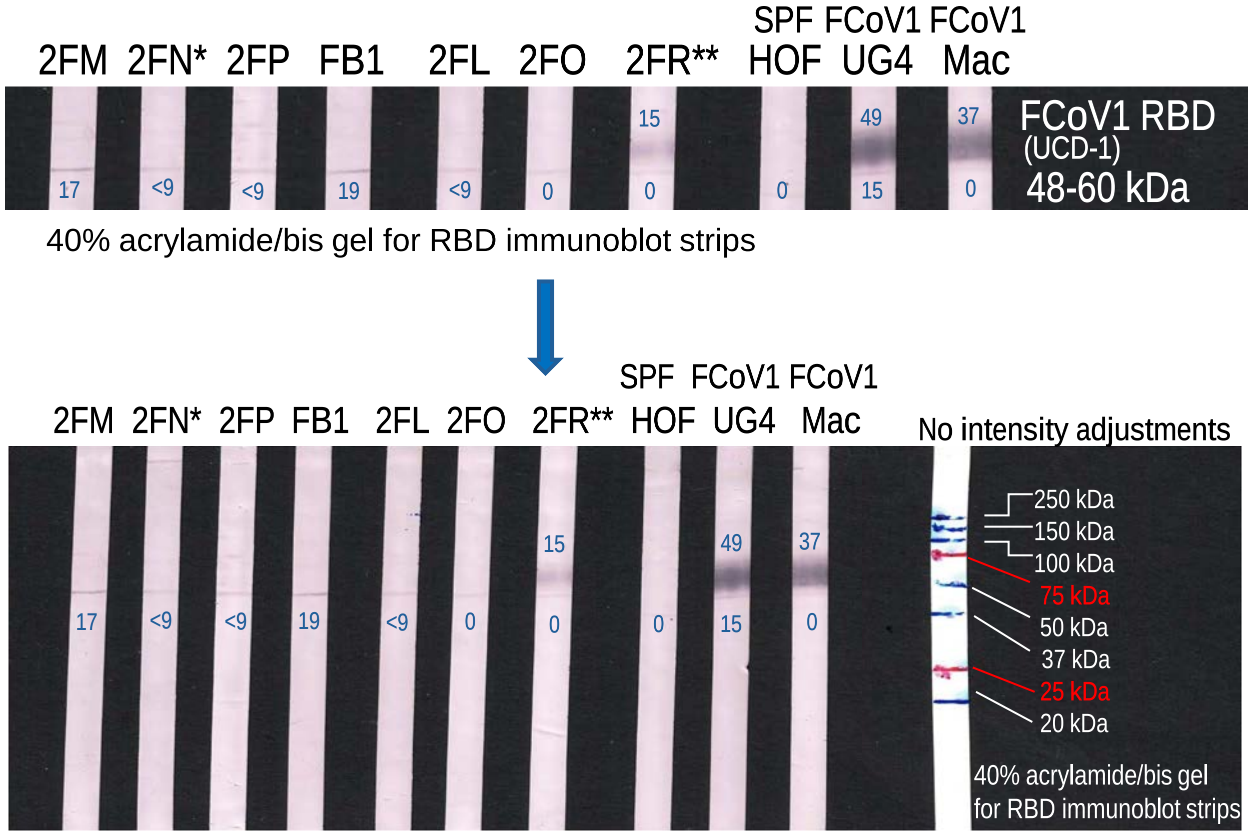

HALO values should be in percentage.

Figure 12C
Figure S8C

Figure 12C
Identical Immunoblot Set 1

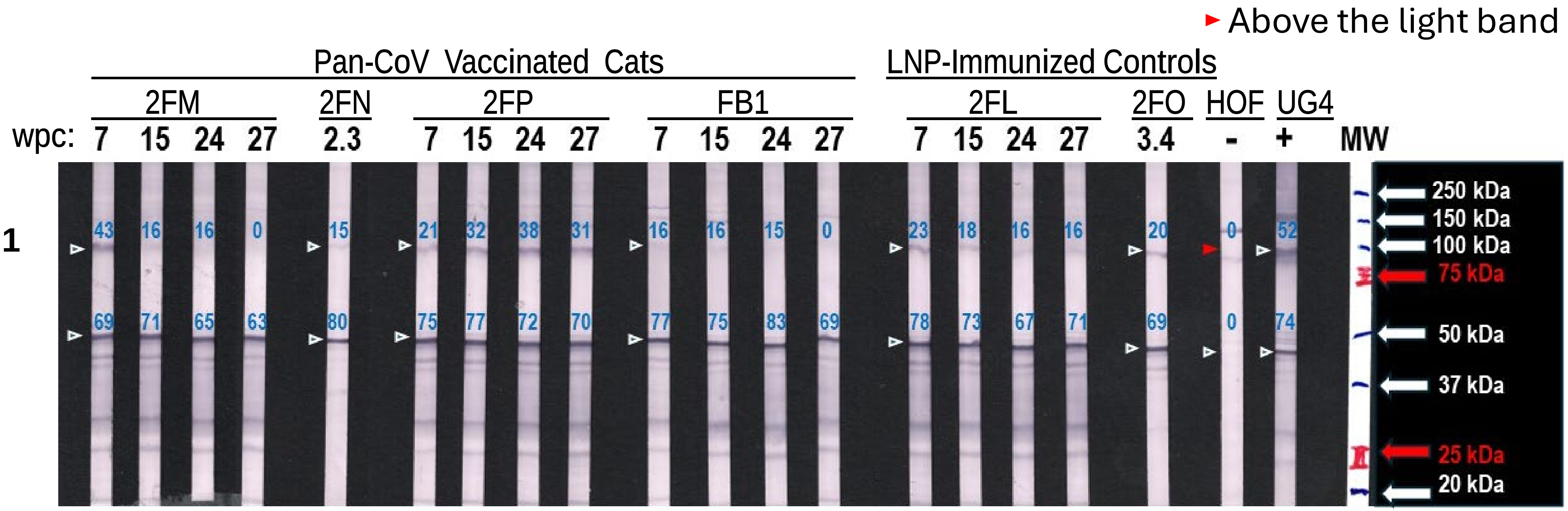

Figure S8C
Identical Immunoblot Set 2

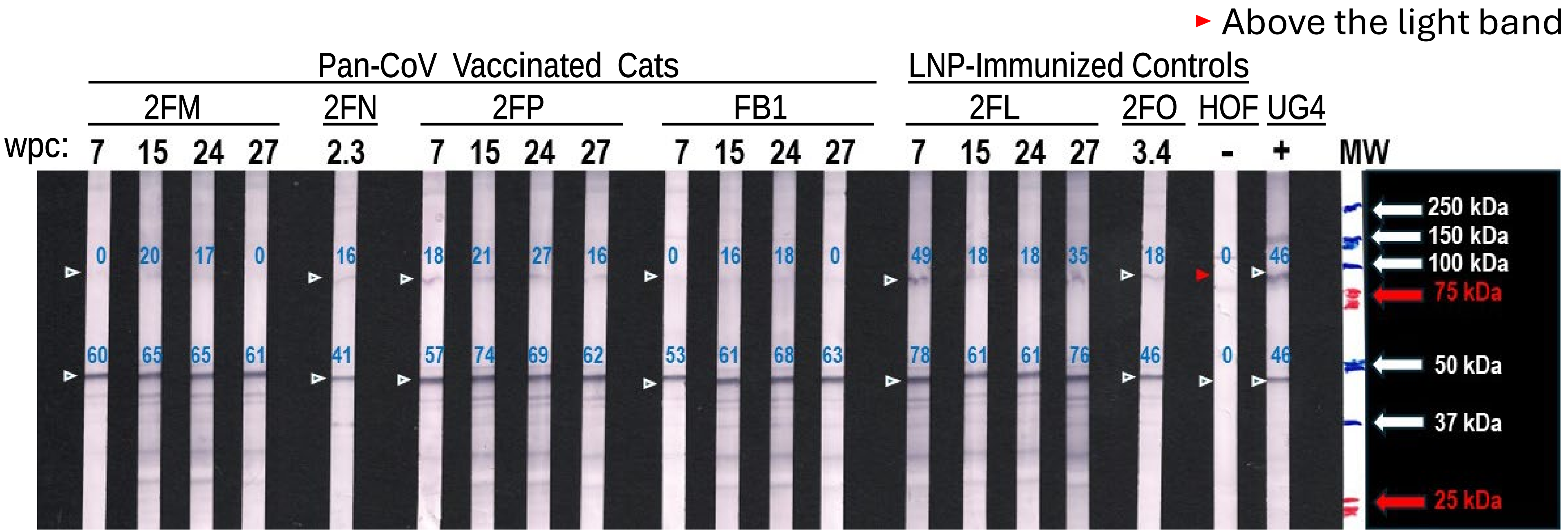

HALO values should be in percentage.

Figure 12A Set 1 in Manuscript
RdRp RT-snPCR
Gel Set 1
(8-21WPC)

Figure 12A in Manuscript (Set 1)

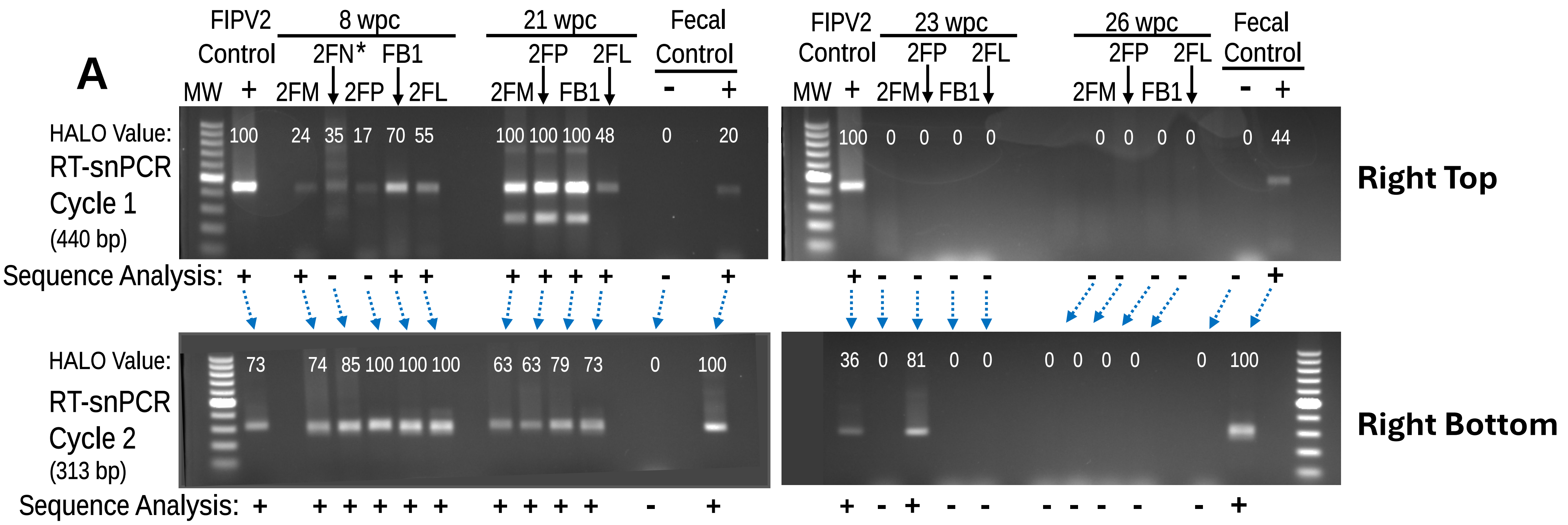

The whole gels of these cut gels are shown in PPT Slides 9 (Left top & bottom) and 10 (right top & bottom)

Figure 12A Left
RdRp RT-snPCR
Gel Set 1
(8-21WPC)

Figure 12A Left top & bottom Duplicate Set 1 8 WPC & 21 WPC

Lane 1: Purified FIPV2 Positive Control
Lane 10: Fecal (SPF) Negative Control
Lane 11: Fecal (FB1) Positive Control

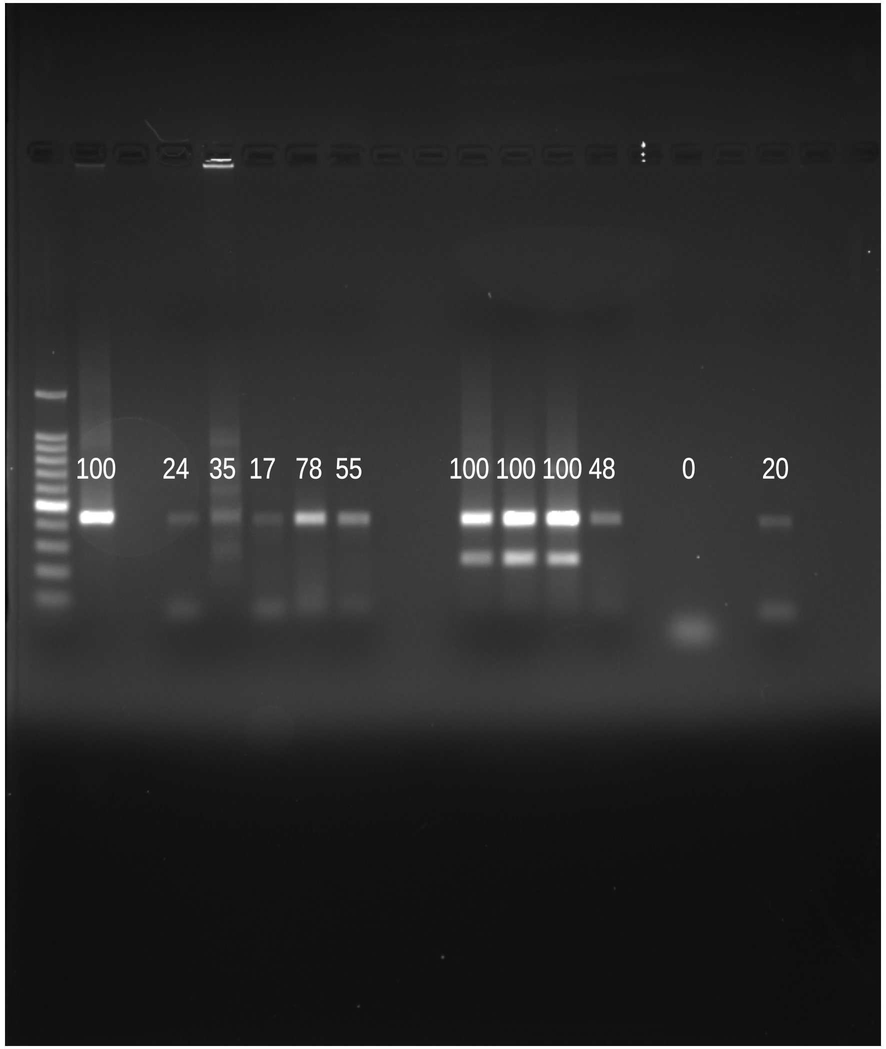

Cycle 1

Lane 1: Purified FIPV2 Positive Control
Lane 10: Fecal (SPF) Negative Control
Lane 11: Fecal (FB1) Positive Control

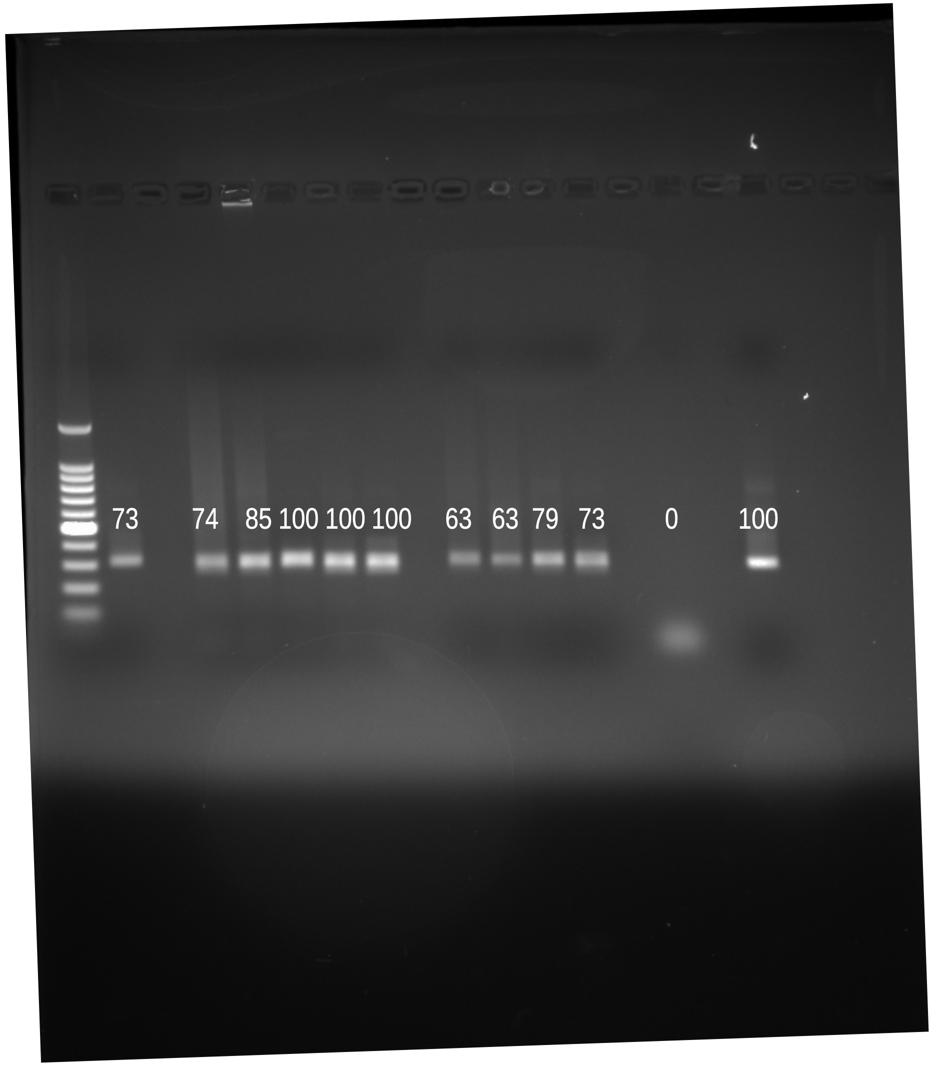

Cycle 2

HALO values should be in percentage.

Figure 12A Right
RdRp RT-snPCR
Gel Set 1
(23-26WPC)

Figure 12A Right top & bottom
Duplicate Set 1
23 WPC & 26 WPC

Lane 1: Purified FIPV2 Positive Control
Lane 10: Fecal (SPF) Negative Control
Lane 11: Fecal (FB1) Positive Control

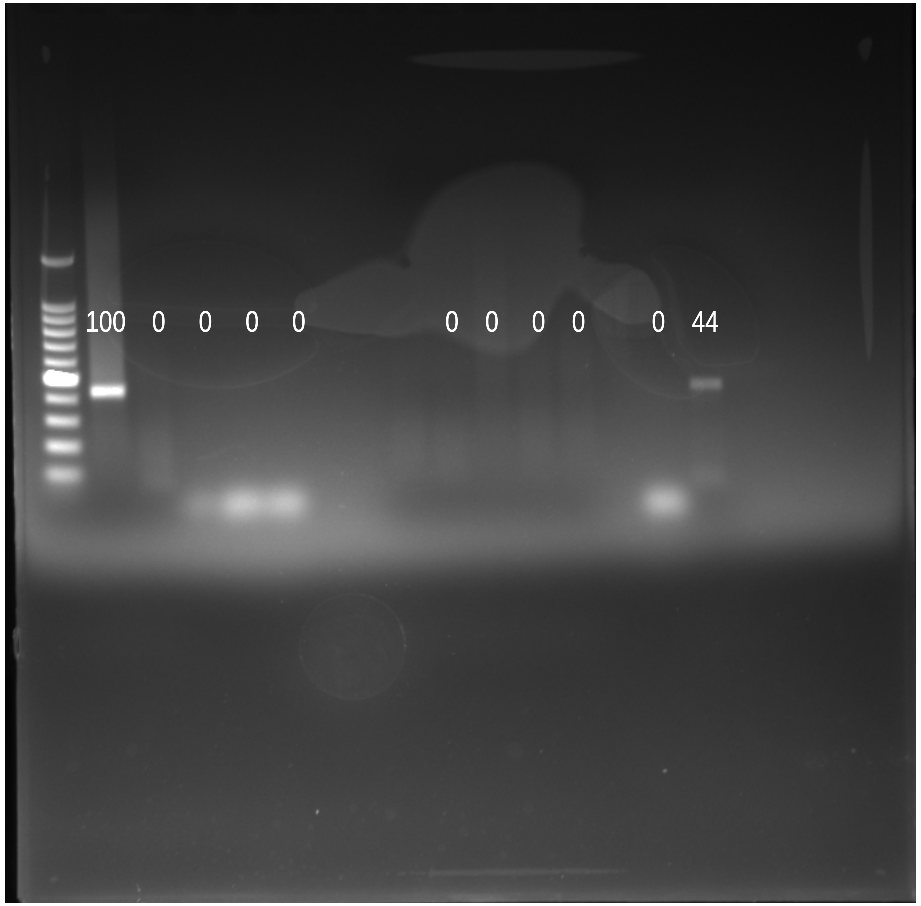

Cycle 1

Lane 1: Purified FIPV2 Positive Control
Lane 10: Fecal (SPF) Negative Control
Lane 11: Fecal (FB1) Positive Control

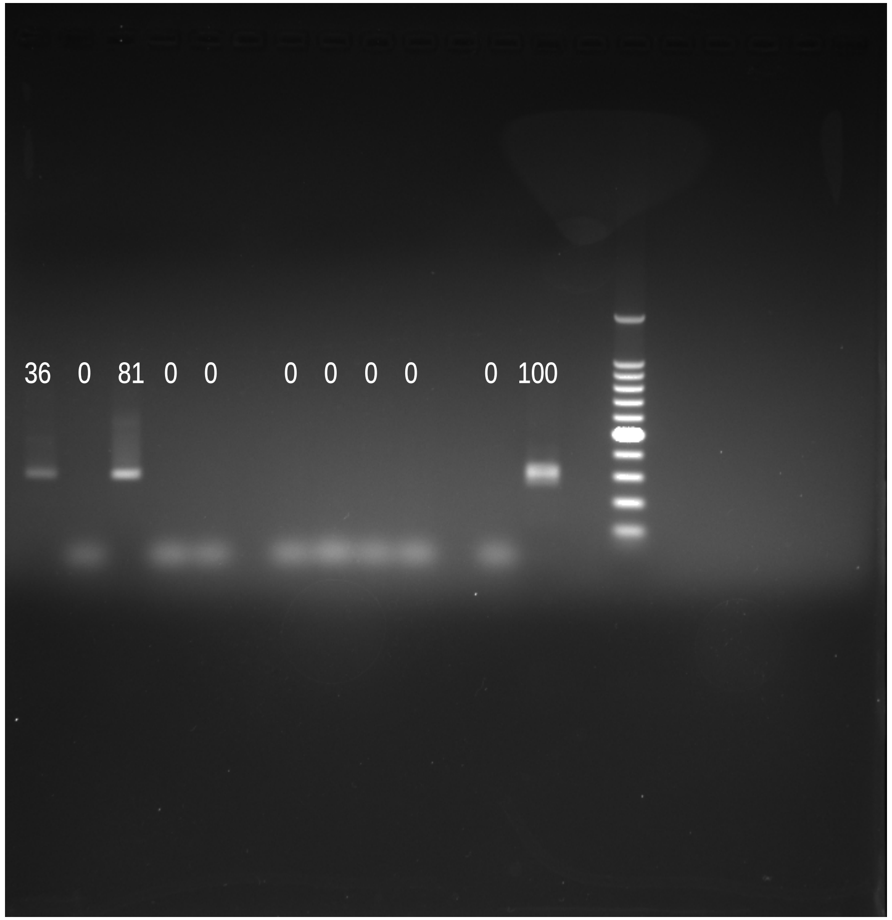

Cycle 2

HALO values should be in percentage.

Figure 12B/S8B Set 2 for Bar Graphs Duplicate

RdRp RT-snPCR
Gel Set 2
(8-21WPC)

Duplicate Set 2
Only HALO Values used in Manuscript for Bar Graph
8 WPC & 21 WPC

Figure 12B

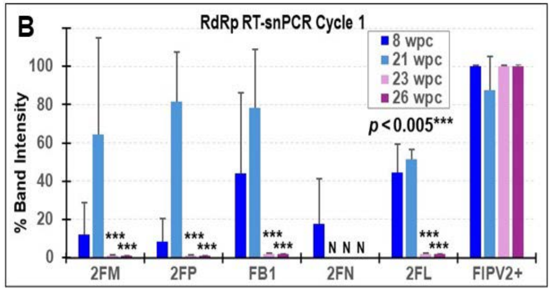

Figure S8B

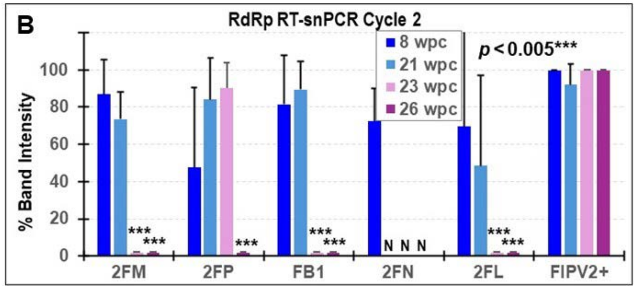

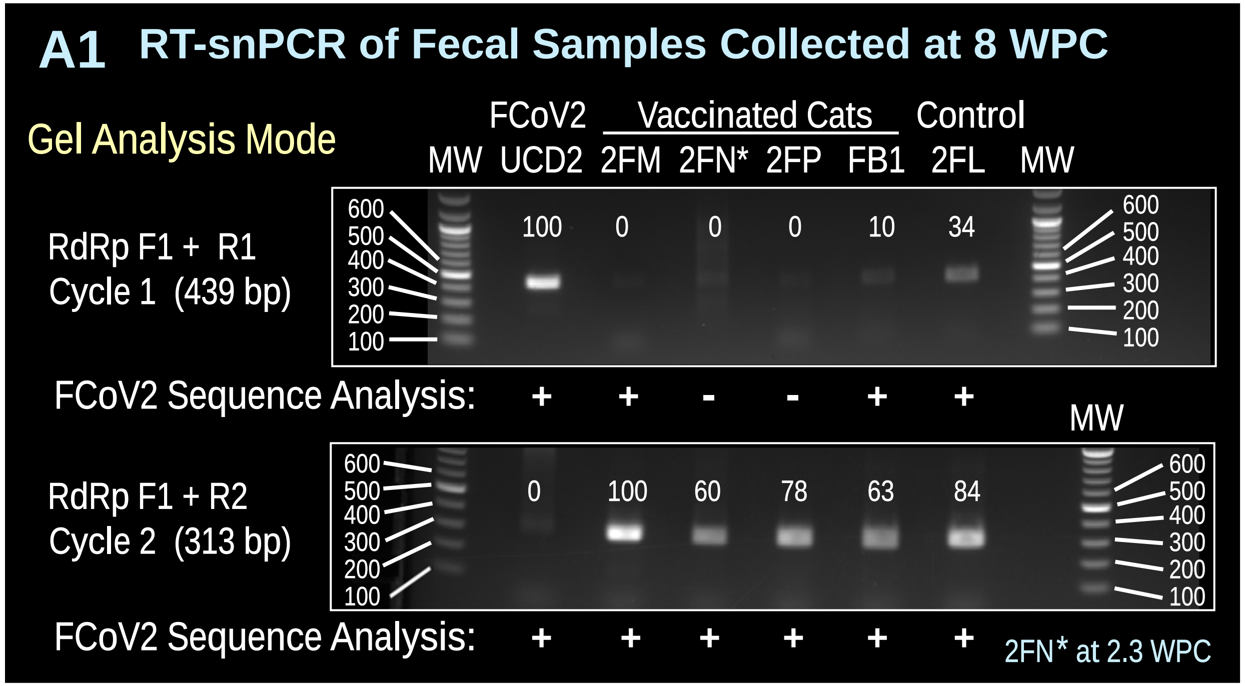

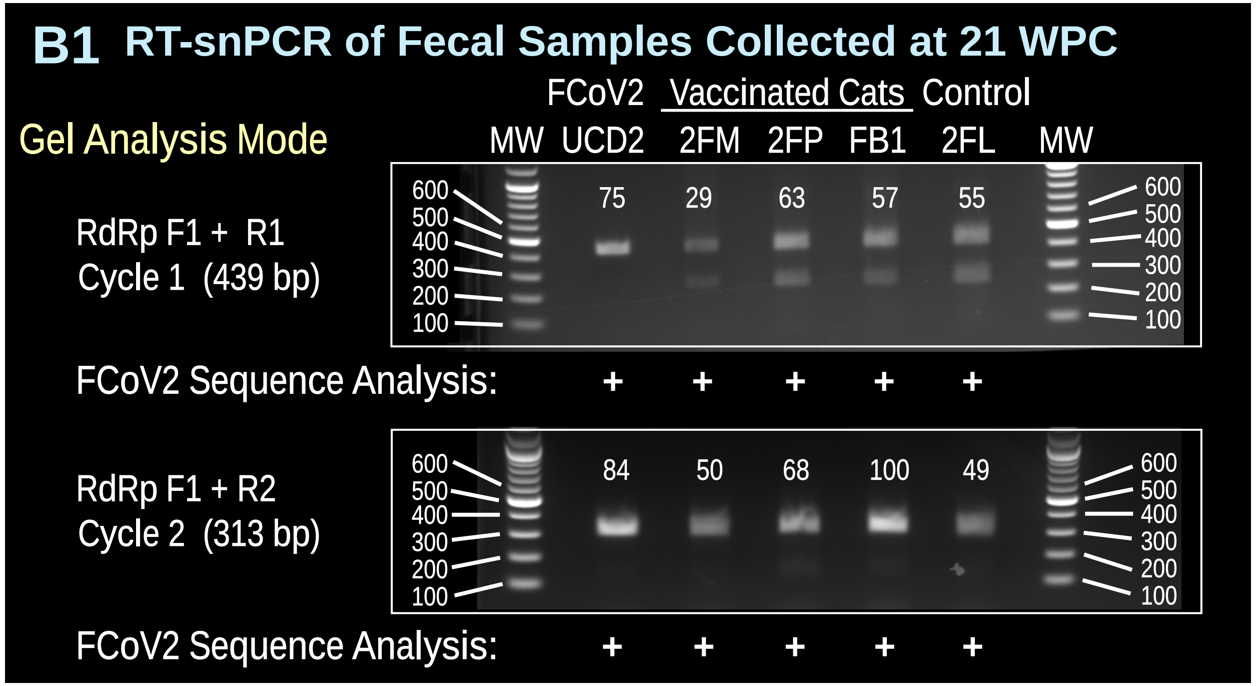

The whole gels of these cut gels are shown in PPT Slides 12 (Left top & bottom) and 13 (right top & bottom)

HALO values should be in percentage.

Figures 12B/S8B for Bar Graph Duplicate (Whole Gels)

RdRp RT-snPCR
Gel Set 2
(8-21WPC)

Duplicate Set 2
Only HALO Values used in Manuscript for Bar Graphs (Figures 12B & S8B)
8 WPC

Figure 12B

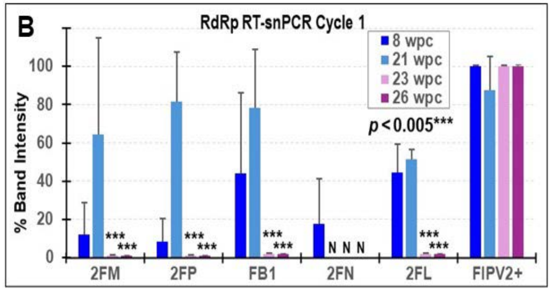

Lane 1: Purified FIPV2 Positive Control
Lane 7: Buffer used as Negative Control

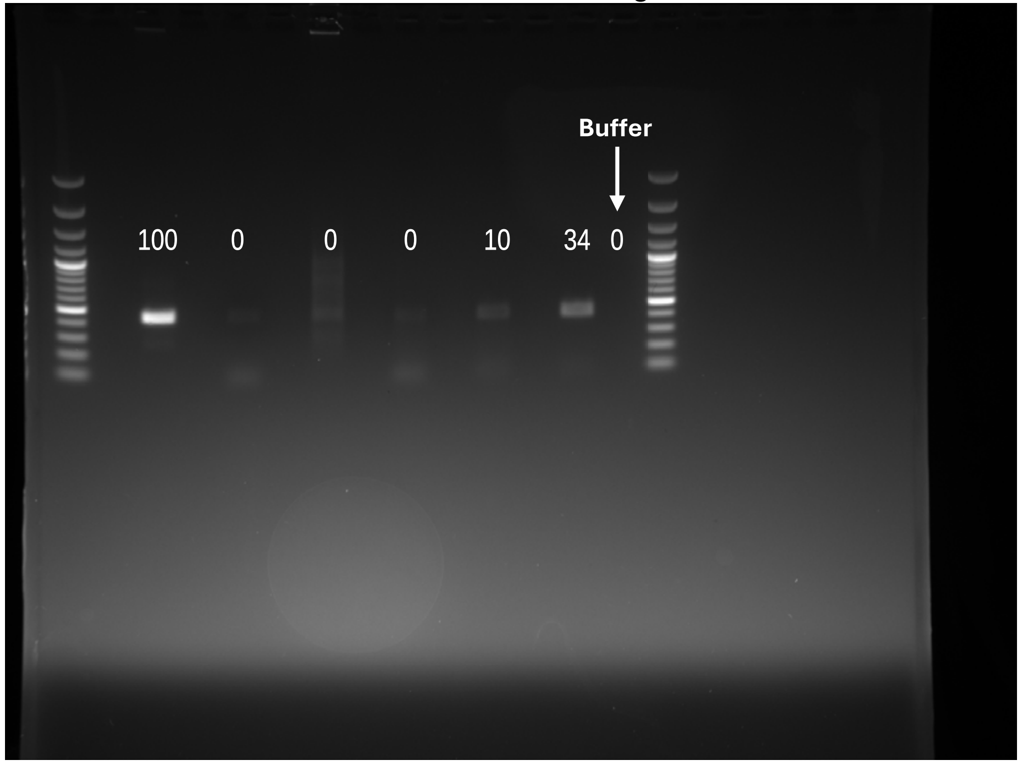

Cycle 1

Figure S8B

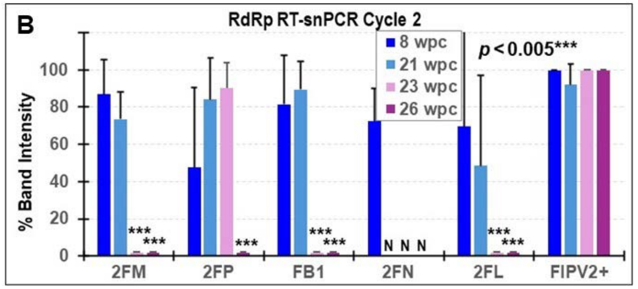

Lane 1: Purified FIPV2 Positive Control
Lane 7: Buffer used as Negative Control

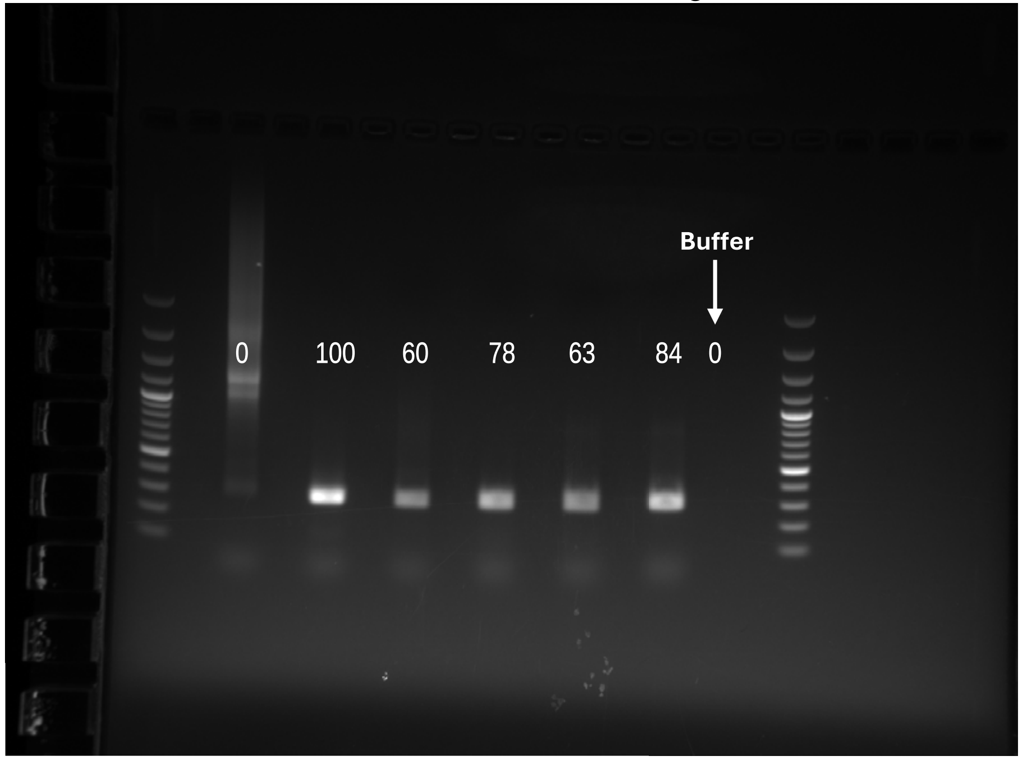

Cycle 2

HALO values should be in percentage.

Figure 12B/S8B for Duplicate Bar Graph (Whole Gels)

RdRp RT-snPCR

Gel Set 2

(8-21WPC)

Duplicate Set 2

Only HALO Values used in Manuscript for Bar Graph (Figures 12B & S8B)

21 WPC

Figure 12B

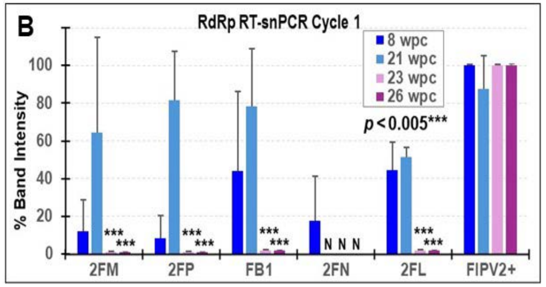

Lane 1: Purified FIPV2 Positive Control

Lane 6: Buffer used as Negative Control

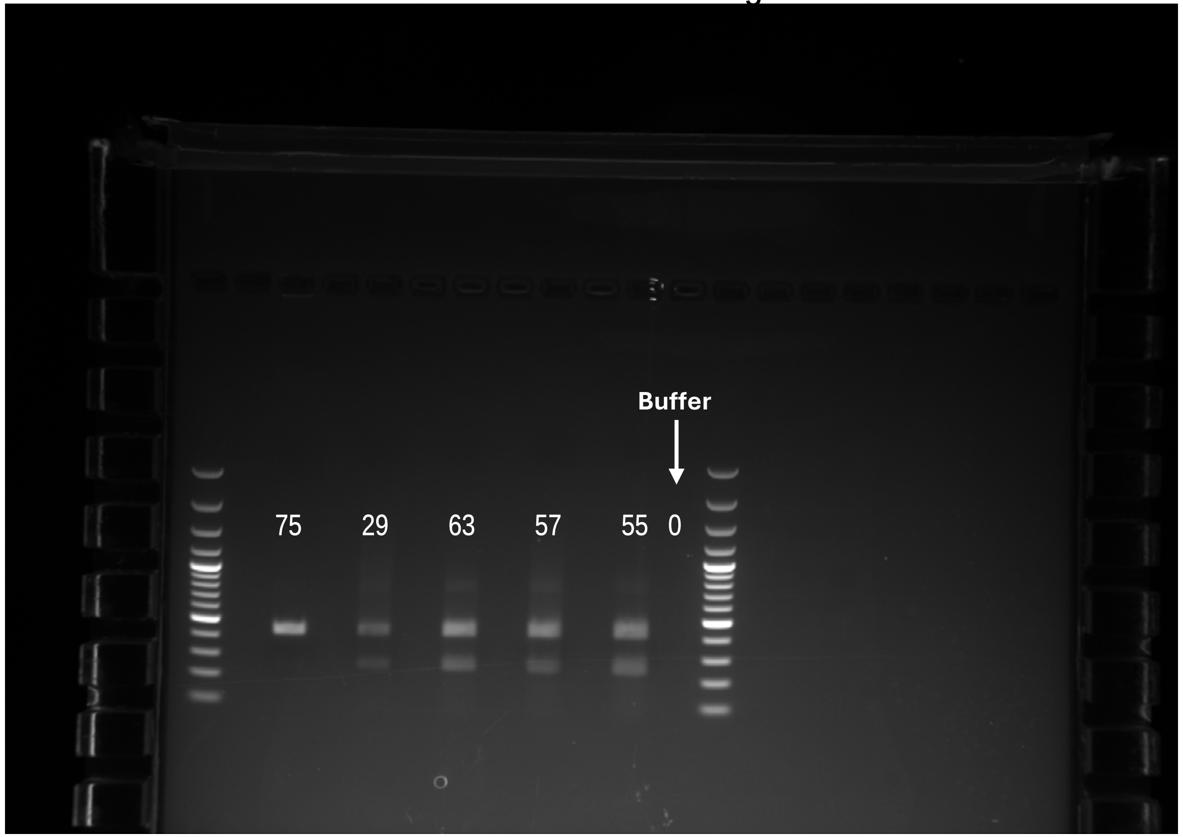

Cycle 1

Figure S8B

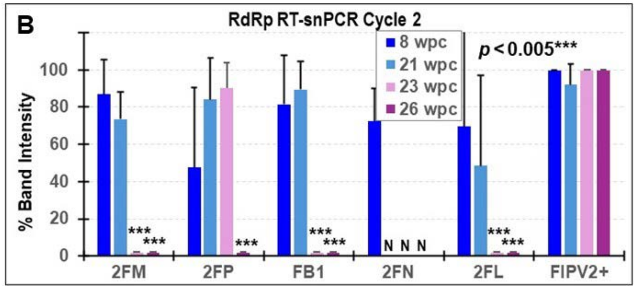

Lane 1: Purified FIPV2 Positive Control

Lane 6: Buffer used as Negative Control

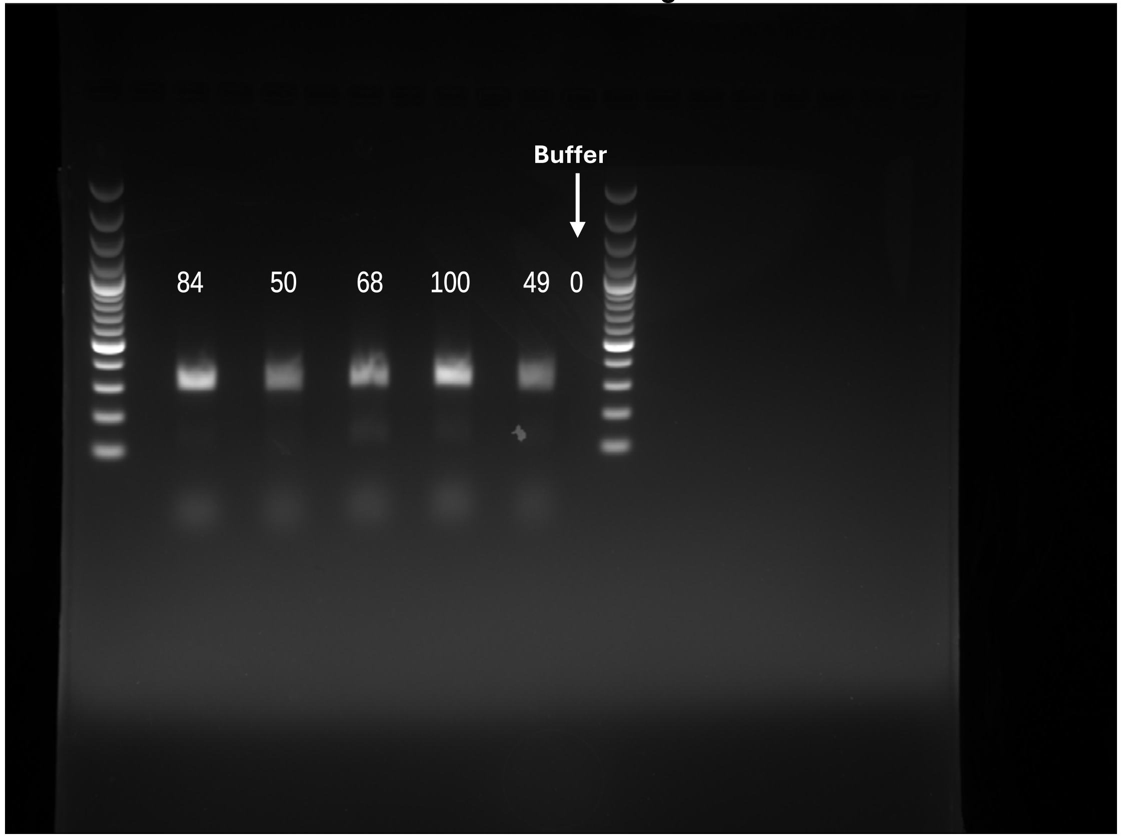

Cycle 2

HALO values should be in percentage.

Figure 12A/S8B Set 2 Duplicate

RdRp RT-snPCR

Gel Set 2 (23-26WPC)

<u>Cycle 1 and Cycle 2 Gels</u>

Lane 1: Purified FIPV2 Positive Control
Lane 2: 2FM at 23 WPC
Lane 3: 2FP at 23 WPC
Lane 4: FB1 at 23 WPC
Lane 5: 2FL at 23 WPC
Lane 6: 2FM at 26 WPC
Lane 7: 2FP at 26 WPC
Lane 8: 2FL at 26 WPC
Lane 9: FB1 at 26 WPC
Lane 10: Fecal (SPF) Negative Control
Lane 11: Fecal (FB1) Positive Control

Duplicate Set 2

Only HALO Values used in Manuscript for Bar Graph (Figures 12B & S8B)

23 WPC & 26 WPC

Figure 12B

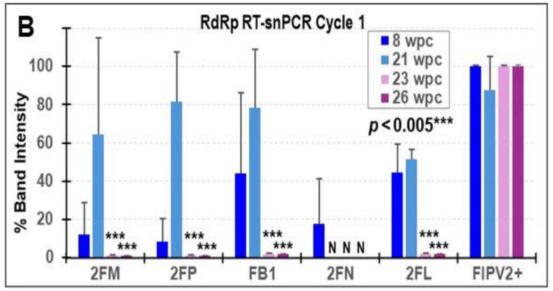

Figure S8B

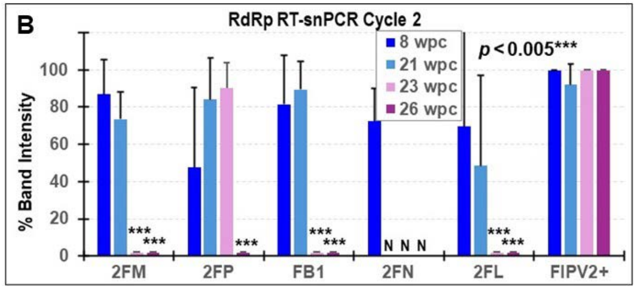

Cycle 1

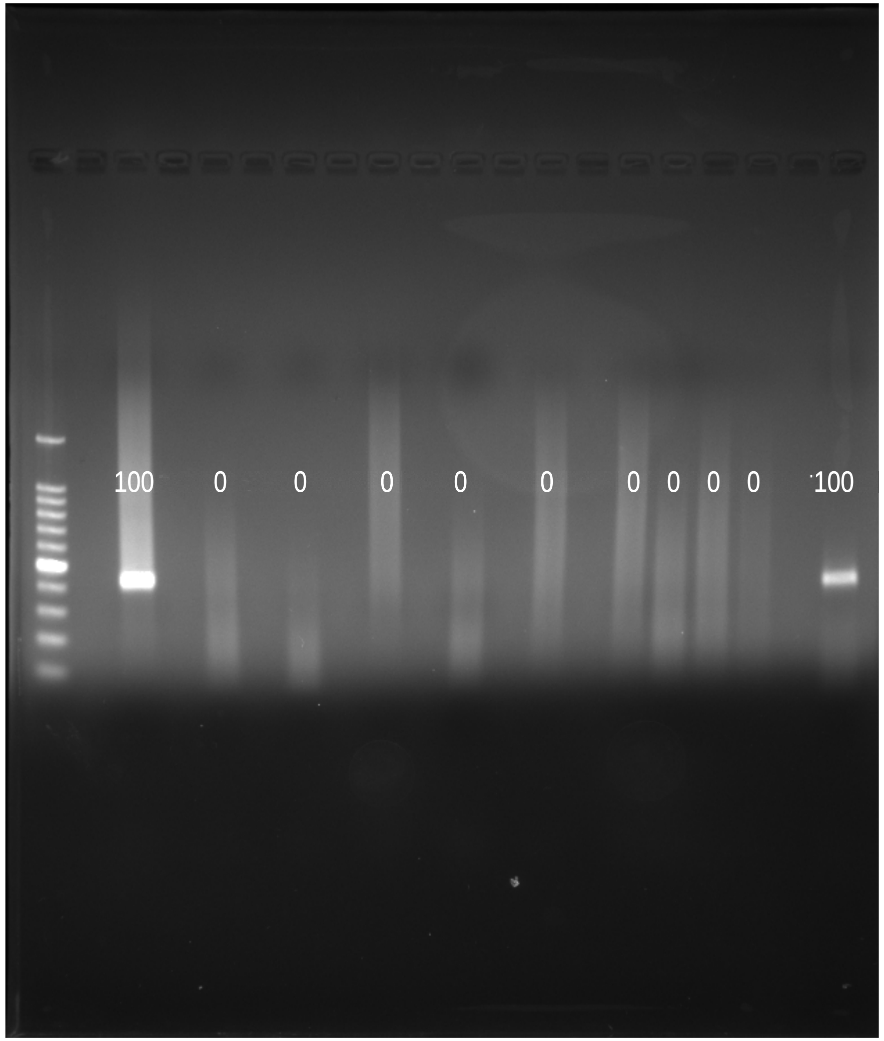

Cycle 2

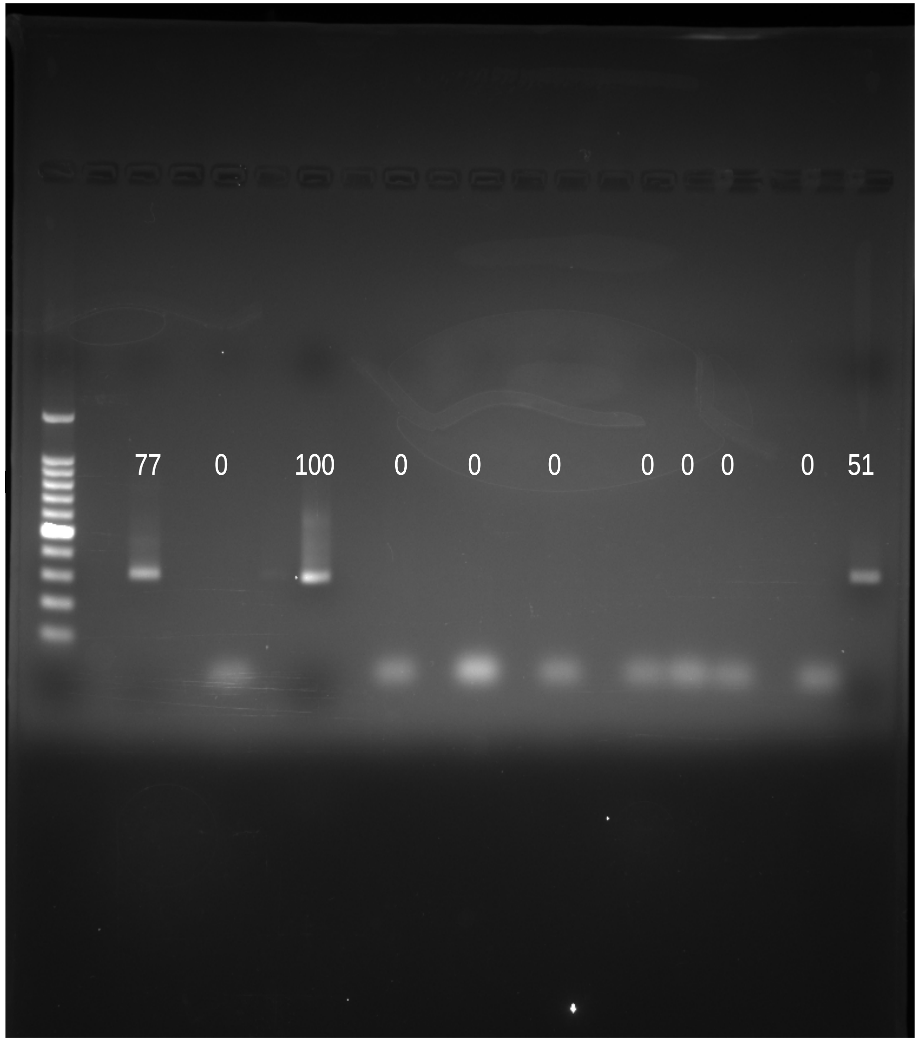

HALO values should be in percentage.

Figure S8A
NSP14 RT-PCR Gel

Figure S8A
8 WPC (Cycle 1)

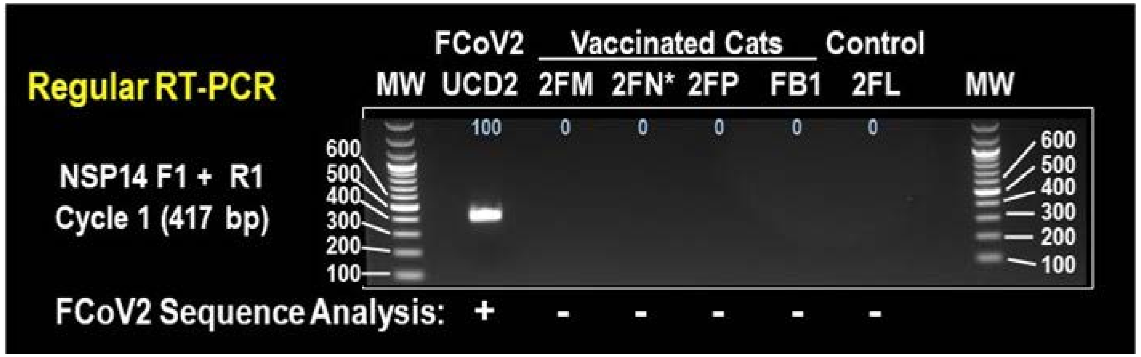

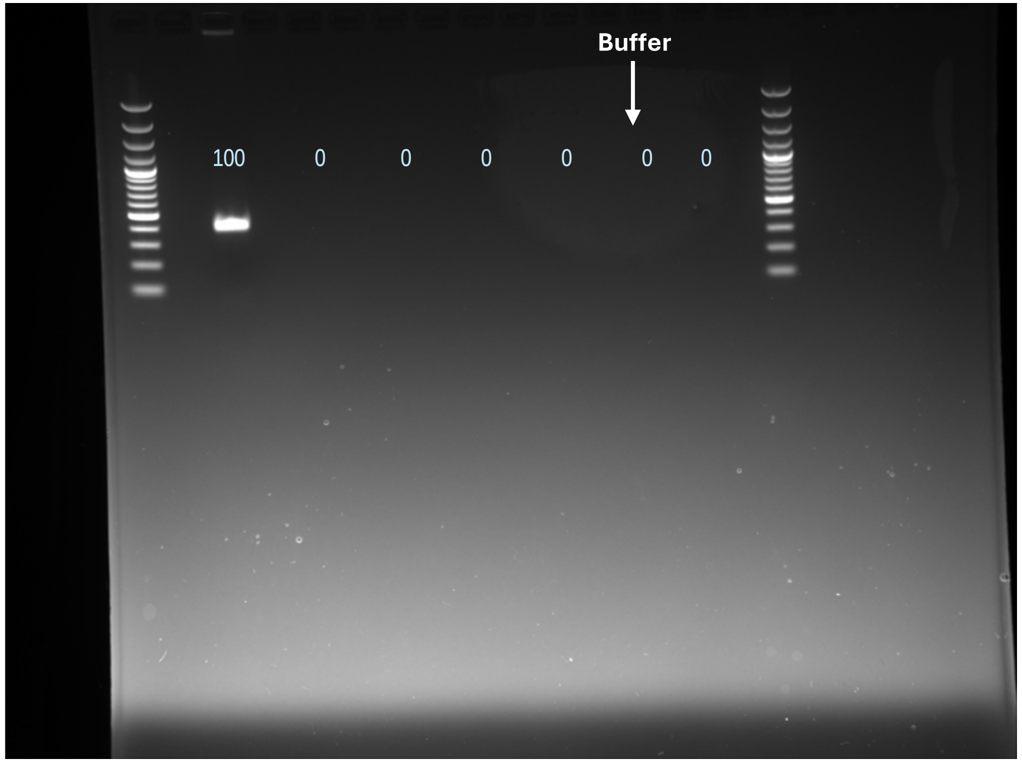

Single Cycle

Lane 1: Purified FIPV2 Positive Control
Lane 7: Buffer used as Negative Control

HALO values should be in percentage.
