# Supplementary material for: Pilot Studies Testing Novel Minimized Pan-Coronavirus (CoV) Vaccines in Feline Immunodeficiency Virus-Infected Cats With or Without Feline CoV Serotype-1 (FCoV1) Coinfection and in Specific-Pathogen-Free Cats Against Pathogenic FCoV2
Source: Vaccines (Basel). 2025 Nov 18;13(11):1172. doi: 10.3390/vaccines13111172 (PMC12656953; doi:10.3390/vaccines13111172)
Supplement: Supplementary file 1 [file vaccines-13-01172-s001.zip › vaccines-3790918-supplementary/vaccines-3790918-supplementary.pdf]

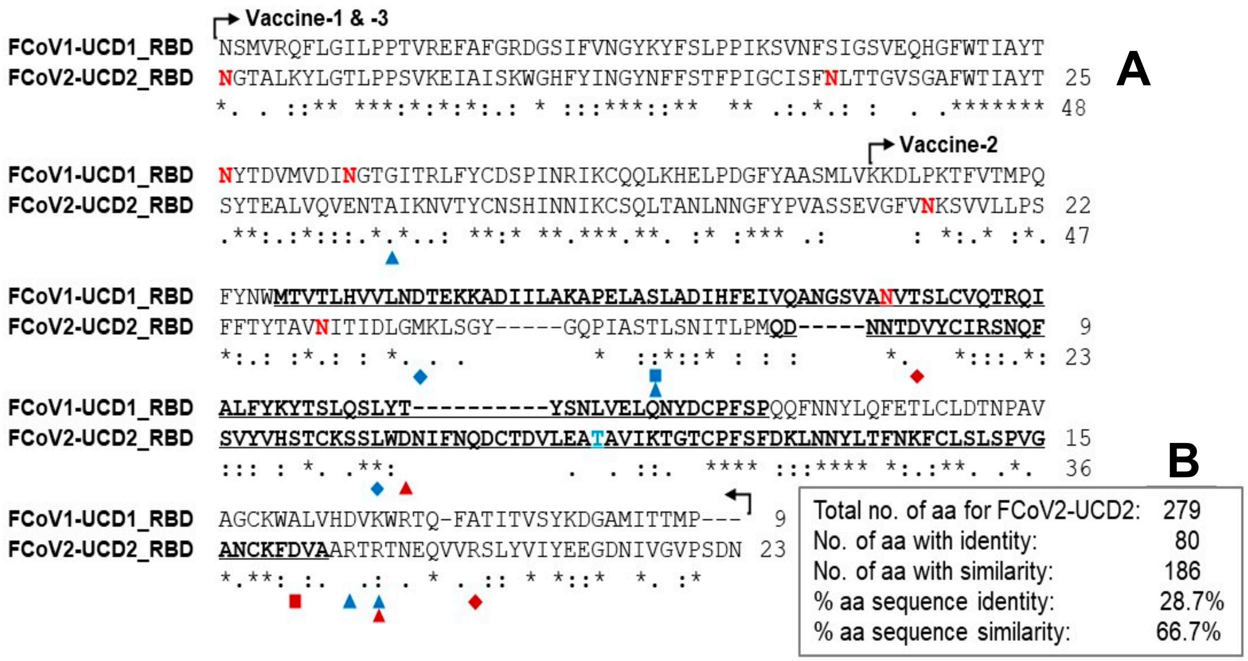

Figure S1. Amino acid (aa) sequence alignment of FCoV1-UCD1 with FCoV2-UCD2. The bolded, underlined aa sequences represent potential receptor binding motifs (RBMs), as previously reported, for FCoV1-UCD1 (Accession #AB088222.1) and FCoV2 79-1146 (Accession #DQ010921.1) [18] (A). Note that FCoV2-UCD2 (Accession #PV941793) has an identical RBD sequence as FCoV2 70-1146. The FCoV1-UCD1 RBD has three N-glycosylation sites with a high prediction, as shown with a bold red N and no O-glycosylation, based on the NetNGlyc 1.0 Server and NetOGlyc 4.1 Server, respectively. FCoV2-UCD2 RBD has four predicted N-glycosylation sites, shown in bold red (**N**), and one O-glycosylation site, in bold bright blue (T). The aa sequence identity and similarity between the two RBD sequences are summarized (B), starting with asparagine (N), with a start arrow and ending with a last aa as proline (P) for FCoV1 UCD1-Ori in pan-CoV vaccine-1 and -3. Another start arrow, closer to the RBM of FCoV1-UCD1, starts with lysine (K) and ends with the last aa as proline (P), is for FCoV1 sRBD in pan-CoV vaccine-2. The six color-coded symbols below the FCoV2-UCD2 sequence represent potential mutation sites affecting the six neutralizing mouse monoclonal antibodies (nMAbs) identified by Kida et al. in blue [35] and Corapi et al. in red [36], and these nMAbs were produced using FCoV2 79-1146. The three nMAbs of Kida et al. do not cross-neutralize the FCoV1 virus [18].

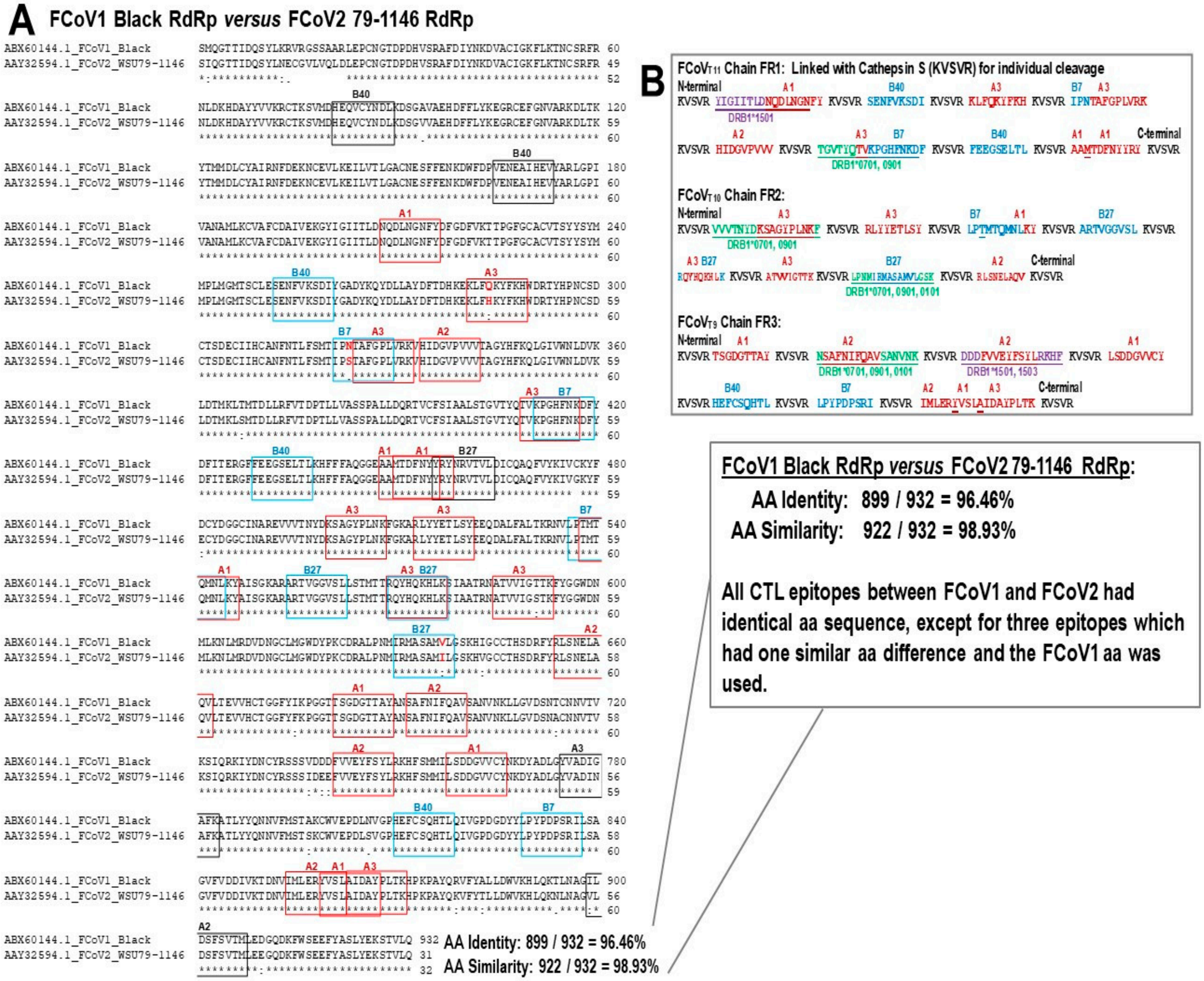

Figure S2. Mapping of 9-mer CTL and 15mer TH epitopes on FCoV1 and FCoV2 RdRp sequences. On the alignment (A), each box represents two 9-mer CTL epitopes with aa residues conserved between FCoV2 WSU79-1146 (Accession #AAY32594.1) and FCoV1 Black (Accession #ABX60144.1). Red and blue boxes are for red-coded HLA-A allotypes and blue-coded HLA-B allotypes, respectively. The CTL epitopes were combined with TH epitopes to develop three FCoV1 CTL/TH chains (B). TH epitopes (green/purple with underlined aa residues with CTL overlap) bind to HLA-DRB1 as described below each lined sequence. The cathepsin S linker (KVSVR) was inserted between the epitopes to serve as a cleavage site for the cellular enzyme, cathepsin S [53].

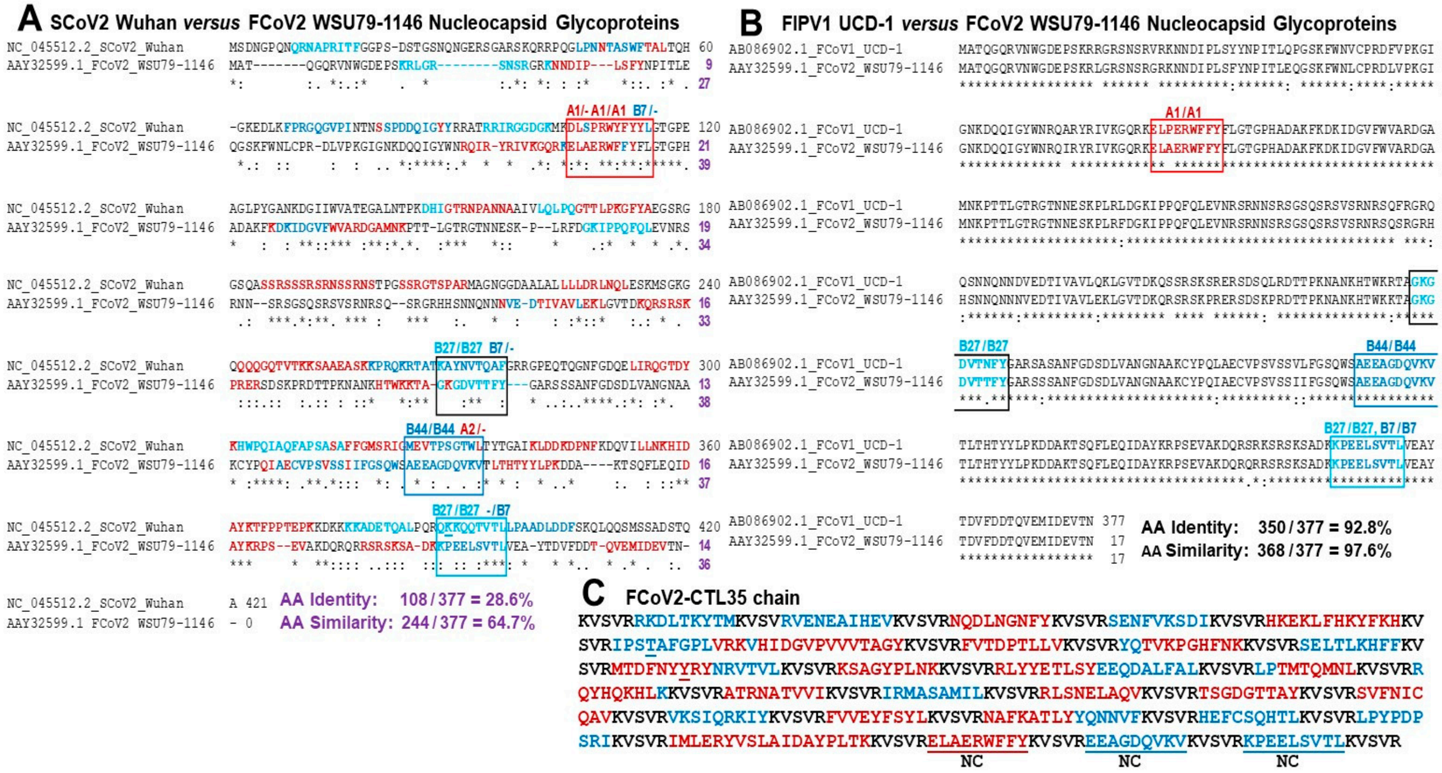

Figure S3. Mapping of 9-mer CTL epitopes on FCoV2 nucleocapsid (NC) for FCoV2-CTL35 chain. A major analysis is performed first with NC sequence alignment with SCoV2-Wuhan (Accession #NC_045512.2) and FCoV2-WSU79-1146 (Accession #AAY32599.1) (A). The CTL epitopes reacting to HLA-A, less common HLA-B*27, and more common HLA-B*7 and HLA-B*44 allotypes are color coded with red, light blue, and blue amino acids (aas), respectively. The CTL epitope matching allotype in both SCoV2 and FCoV2 sequences has a box colored with the same color as the allotype, except for the black box. The black box represents the CTL epitope omitted from the FCoV2-CTLA35 chain. The percent aa sequence identity and similarity between these sequences are shown at the end of the alignment sequences (A). The four CTL epitopes are next transferred to the counterpart CTL epitope on FCoV1-UCD1 (Accession #AB086902.1) and FCoV2-79-1146 (Accession #AAY32599.1) NC sequence alignment (B). This comparison determines the sequence conservation between FCoV1 and FCoV2 CTL epitopes. The percent aa sequence identity and similarity between these sequences are shown at the end of the alignment sequences (B). The three FCoV2/SCoV2-conserved CTL epitopes from NC are included in the C-terminal end of the FCoV2-CTL35 chain (C), as shown with an underlined epitope above NC. The remaining CTL epitopes in this chain are determined by comparing FCoV2 RdRp with FCoV1 RdRp. The FCoV2 aa is used for this chain when an aa difference existed between FCoV2 and FCoV1 CTL epitope sequences. Red and blue aa sequences represent red-coded HLA-A allotypes and blue-coded HLA-B allotypes, respectively.

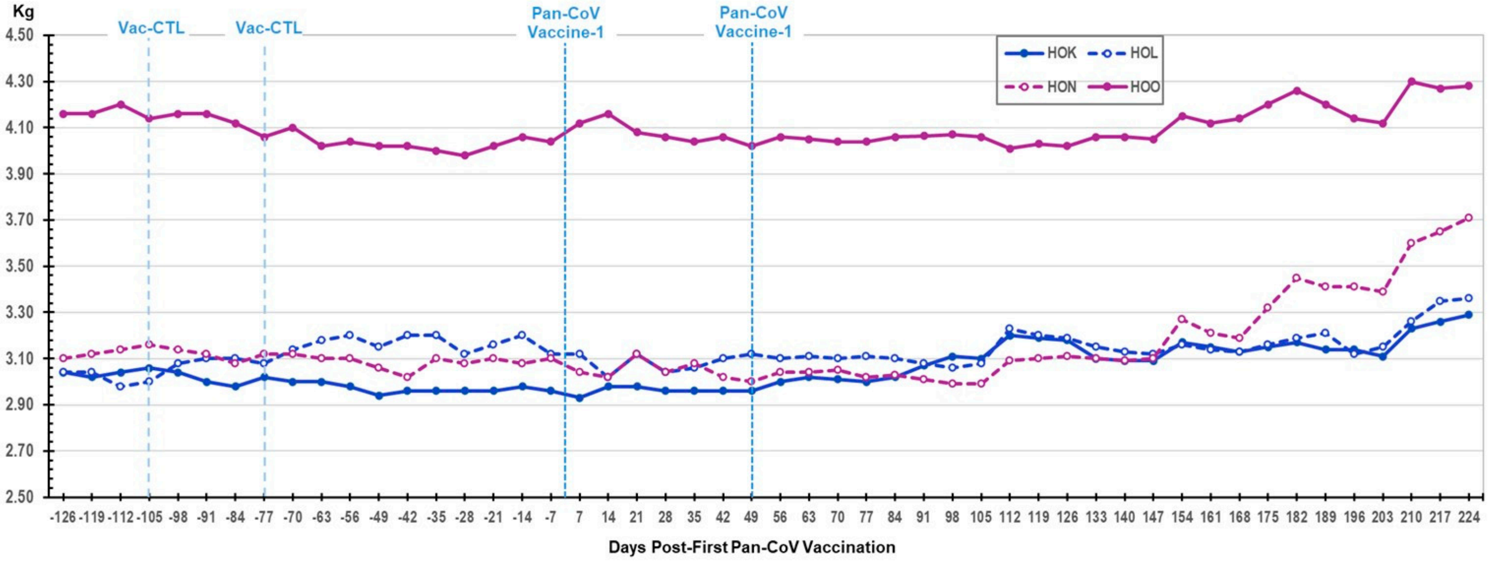

Figure S4. Comparing weight changes between the vaccinated FIV-infected cats and unvaccinated FIV-infected cats in Pilot Study 1. The weights are divided into vaccinated (HOK and HOL, blue lines) and unvaccinated (HON and HOO, maroon lines) FIV-infected cat groups. One cat from each group was also coinfected with FCoV1 (HOL and HON, dotted open circle lines) at the time of vaccinations. Cat HOL received all four vaccinations on -105, -77, 0, and 49 days post-first pan-CoV vaccination (dp1v) on day 0. Cat HOK received three vaccinations on -77, 0, and 49 dp1v. The weight of each cat was taken weekly throughout the study. Additionally, HOL received oral prednisolone to prevent diarrhea throughout the study.

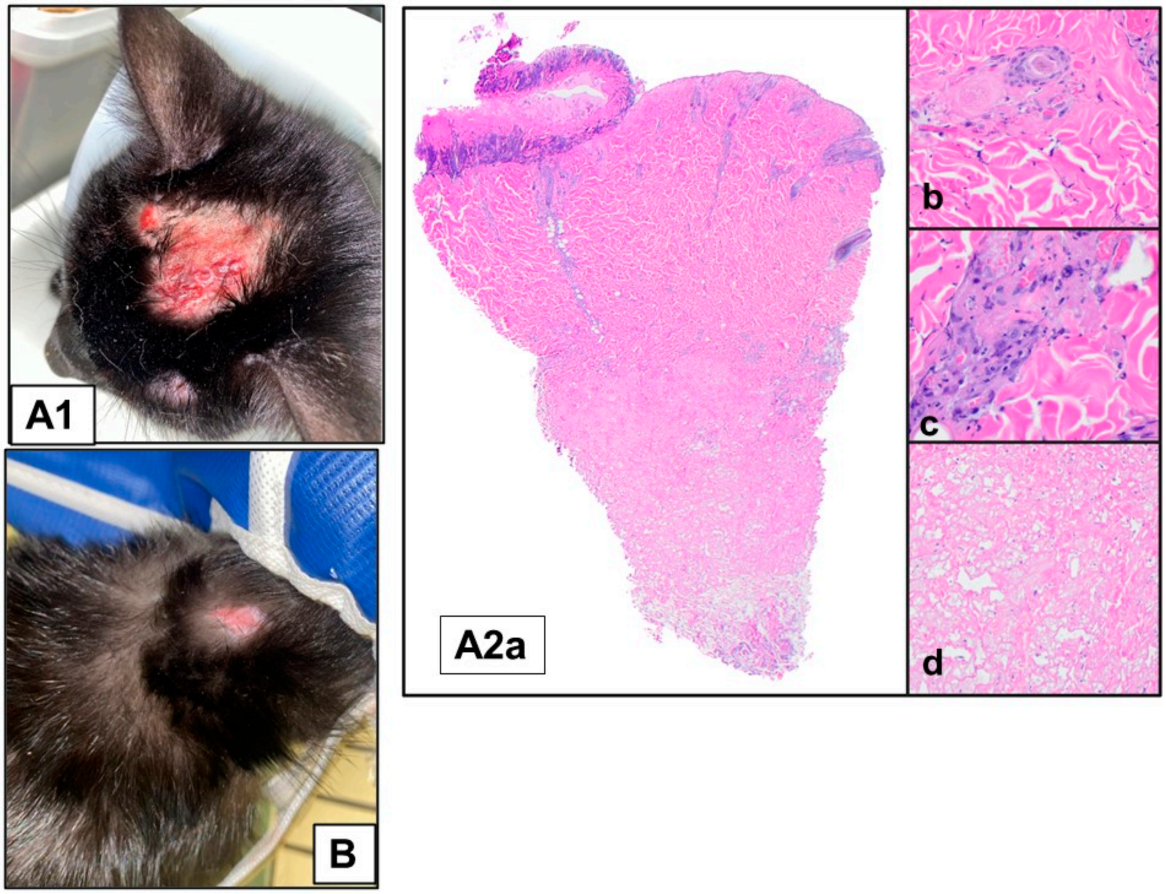

Figure S5. Skin lesions of a FIPV2-challenged/vaccinated cat 2FN. Multifocal areas of erythema, erosion, and ulceration extended from the head (A) to the neck (B) in few days. A photomicrograph of the lesions shows a cutaneous necrotizing vasculopathy (A2a), characterized by fibrinoid necrosis of blood vessels (A2b), leukocytoclasia (A2c), and large lakes of coagulative necrosis (A2d).

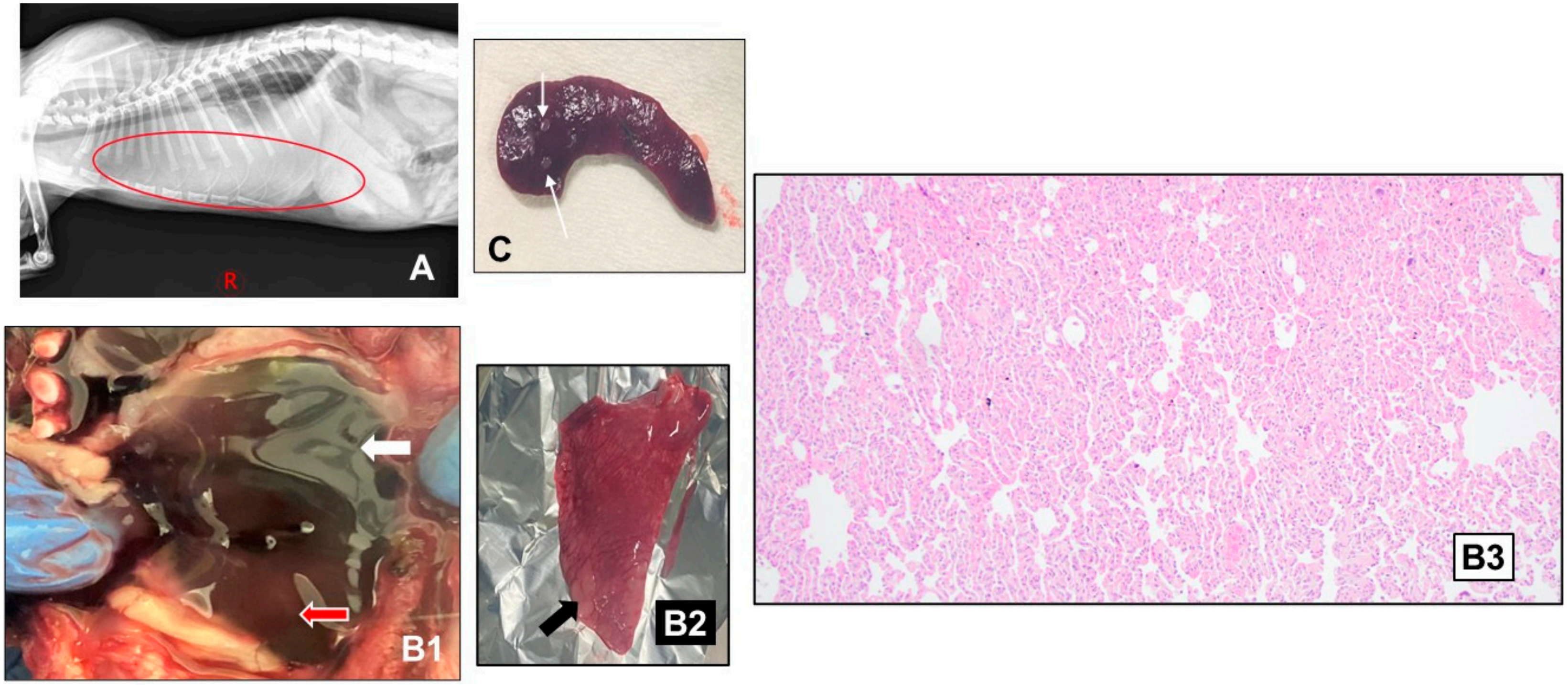

Figure S6. FIP pathology of unimmunized control cat 2FR. A radiographic image shows signs of pleural effusion (highlighted with a red oval) (A), which supports free fluid within the pleural space. The gross examination of the thoracic cavity (B1) reveals effusion into the pleural space (white arrow). The right lung displays a gelatinous material (red arrow). The excised left lung lobe (B2) is diffusely dark red and glistening (congestion), and pleural surface is scattered with fibrinous plaques (black arrow). A photomicrograph of sections of the lung shows evidence of atelectasis (B3). The spleen is rounded and congested, with multifocal, white, loosely adhered, capsular plaques (C).

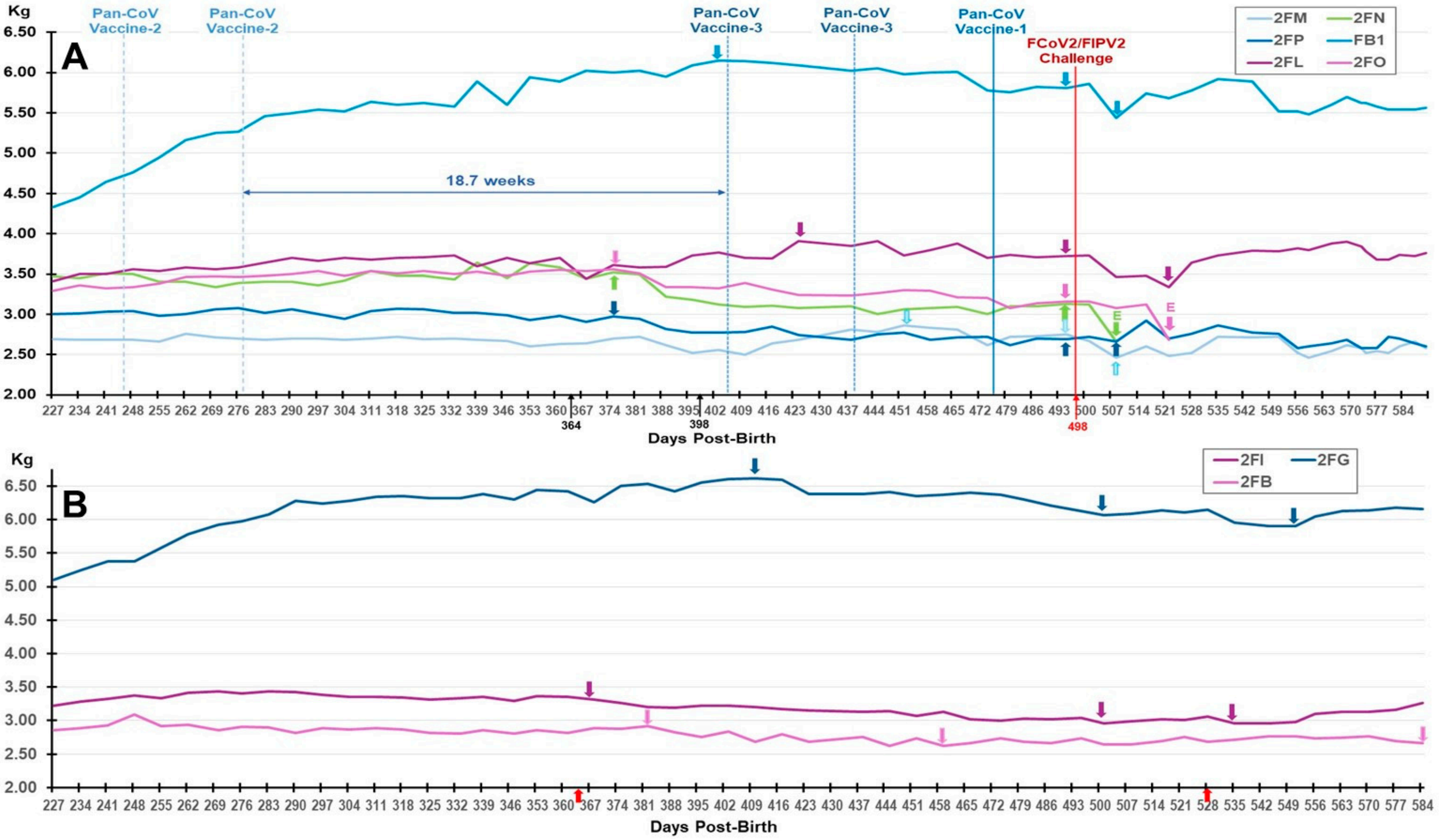

Figure S7. Comparing of weight changes between the cats in Pilot Study 2 and their lineage-related unvaccinated/unchallenged SPF cats. The weights of each cat were taken weekly during vaccination and twice a week after one wpc, to evaluate the safety of multiple vaccinations and the safety of challenge infection (A). The weights of four vaccinated cats and two LNP-control cats are shown from days 227 to days 584 post-birth. The first, second, and third arrows for each cat represent the highest weight at post 1-year-old (black arrow on X-axis at 364 days), three days before challenge at day 495, and the lowest weight after challenge. The lowest weight at post-challenge was on day 508 for vaccinated cats and day 522 for LNP-control cats, respectively. The second black arrow on X-axis at 398 days is the date when FB1 is one year old. The weights of the generation-4 littermates (2FL, 2FM, 2FN, 2FO, 2FP) and related male (FB1) in Pilot Study (A) are compared to the age-matched weights from generation-1 (2FB) and generation-2 (2FG, 2FI) unvaccinated SPF littermates (B). The first, second, and third arrows for unvaccinated SPF cats point to the highest weight at post 1-year-old (red arrow on X-axis at 364 days), the lowest weight between days 364-528 (red arrow on X-axis at 528 days), and the lowest weight between days 529-584.

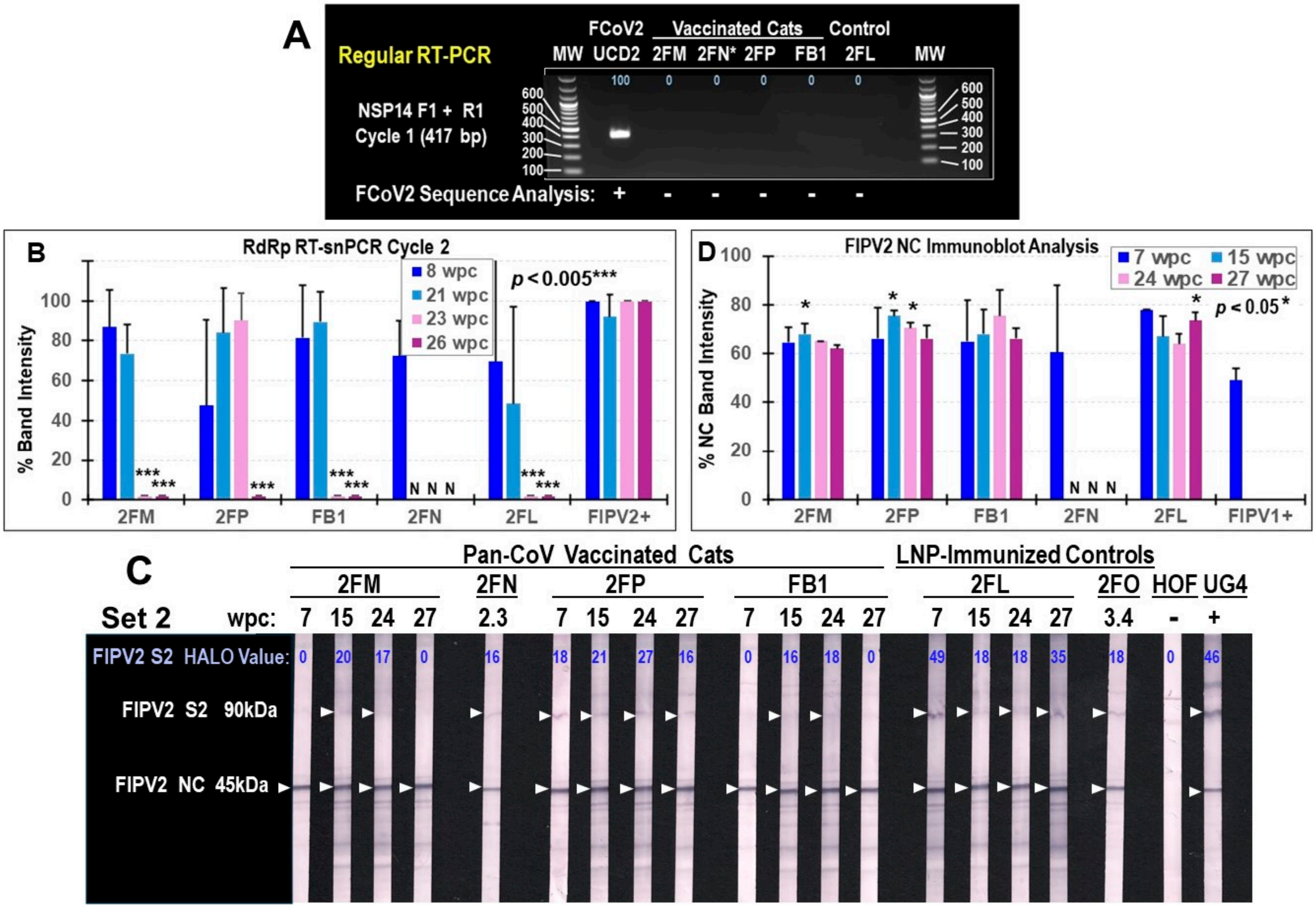

Figure S8. NSP14 RT-PCR, RdRp RT-snPCR, and FIPV2 Immunoblot Analyses. The fecal samples from vaccinated cats (2FM, 2FN*, 2FP, FB1) and LNP-immunized 2FL at 8, 21, 23, and 26 wpc were monitored for fecal FIPV2 shedding first by NSP14 RT-PCR (A). Fecal sample for 2FN (*) was collected at 2.3 wpc during necropsy. Bar graph of RT-snPCR Cycle 2 results had significant differences between those samples collected at 23 and 26 wpc and the fecal positive control (FIPV2+), except for 2FP's fecal sample at 23 wpc (B). The fecal positive control was FB1's cDNA obtained by reverse transcription of RNA from his feces collected at 8 wpc. 2FN's samples were not available (N) for testing on 21, 23, and 26 wpc. The duplicate Set 2 immunoblot reactivity of vaccinated and LNP-control cats, identical to those sera used in Set 1 (Figure 12C), is shown (C). The bar graph of HALO average values, derived from immunoblot Set 1 (Figure 12C) and Set 2 (8C), shows sporadic statistical difference between the average value at each time point and the average value of FIPV1 positive control (D). However, no significant difference was observed between NC band intensities of each vaccinated cat and those of LNP-control 2FL. Thus, endpoint titration of NC bAbs was performed to determine the titer of NC bAbs for each time point as shown in Figure 12C.

Table S1. Laboratory cats used in Pilot Studies 1 and 2

Pilot Study	Cat Code	Age [a]	Sex	Pre-Vaccination Infection Status [a]	Vaccination or Immunization [a]	Post-Vaccination Infection/Challenge [a]	Current Status [a]
**Study 1**	**HOK**	5.3–6.5 year	F	FCoV1-Neg / FIV	Vac-CTL+Vac-1	FCoV1-Neg / FIV	FIV wasting syndrome (Eu)
	**HOL**	5.3–6.5 year	F	FCoV1 / FIV	Vac-CTL+Vac-1	FCoV1 / FIV	FIV wasting syndrome (Eu)
	**HON**	5.3–6.5 year	F	FCoV1 / FIV	None	FCoV1 / FIV	FIV wasting syndrome (Eu)
	**HOO**	5.3–6.5 year	M	FCoV1-Neg / FIV	None	FCoV1-Neg / FIV	FIV wasting syndrome (Eu)
**Study 2**	**2FM**	227-583 days	F	SPF	Vac-2-3-1	FIPV2	Live
	**2FN**	227-583 days	F	SPF	Vac-2-3-1	FIPV2	FIP, Euthanized at 2.3 wpc
	**2FP**	227-583 days	F	SPF	Vac-2-3-1	FIPV2	Live
	**FB1**	193-525 days	M	SPF	Vac-2-3-1	FIPV2	Live
	**2FL**	227-583 days	F	SPF	LNP	FIPV2	Live
	**2FO**	227-583 days	F	SPF	LNP	FIPV2	FIP, Euthanized at 3.4 wpc
	**2FR**	59-163 days	M	SPF	None	FIPV2	FIP, Euthanized at 5.3 wpc
	**2FB**	227-584 days	F	SPF	NA	SPF	Retrospective weight control
	**2FI**	227-584 days	F	SPF	NA	SPF	Retrospective weight control
	**2FG**	227-584 days	M	SPF	NA	SPF	Retrospective weight control

[a] Abbreviations: age in years (year); female (F); male (M); cleared FCoV1 infection by single housing (FCoV1-Neg); specific pathogen free (SPF); CTL vaccine twice (Vac-CTL); pan-CoV vaccine-1 twice (Vac-1) in Study 1; pan-CoV vaccine-2 twice followed by pan-CoV vaccine-3 twice, and pan-CoV vaccine-1 once (Vac-2-3-1); lipid nanoparticle (LNP); not applicable (NA); FIV-infected cat which cleared FCoV1 infection by single housing (FCoV1-Neg / FIV); FCoV1 / FIV-coinfected cat (FCoV1 / FIV); serotype-2 feline infectious peritonitis virus (FIPV2); euthanized (Eu); feline infectious peritonitis (FIP); weeks post-challenge (wpc).

Table S2. Comparison of weight loss and peak XbAbs to S2

Cat ID Code	Start of Weight Loss (Figure 9B) [ab]	Peak XbAbs to S2 (Figures 10A &10B) [ab]	Comments [bc]
**2FM**	Post-4th vaccination	Post-4th vaccination	Live, start to clear FIPV infection at 23 wpc
**2FN**	15.1wk post-2nd vaccination	Post-2nd vaccination	Euthanized at 2.3 wpc (Figure S6)
**2FP**	Post-3rd vaccination	Post-4th vaccination	Live, start to clear FIPV infection at 26 wpc
**FB1**	Post-3rd vaccination	Post-2nd vaccination	Live, start to clear FIPV infection at 23 wpc
**2FL**	Post-4th immunization	NA	Live, start to clear FIPV infection at 23 wpc
**2FO**	15.1wk post-2nd immunization	NA	Euthanized at 3.4 wpc
**2FR** [d]	Acute weight loss at 1.3 wpc	NA	Euthanized at 5.3 wpc (Figure S5)

[a] The start of weight loss for all cats, except 2FR, is shown in Figure 9B. 2RF had acute (14%) weight loss at 1.3 wpc.
The peak XbAbs to S2 for 2FM, 2FN, 2FP, and FB1 are shown in Figures 10A and 10B.

[b] Abbreviations: weeks (wk), weeks post-challenge (wpc), not applicable (NA).

[c] Necropsy/pathology results are shown in Figure S6 for 2FN and Figure S5 for 2FR.

[d] 2FR was 18.1 weeks-old when he was inoculated intranasally with FIPV2, which caused FIP disease, including 14% weight loss.

Table S3. Complete blood count (CBC), blood chemistry, FIPV2 load, and clinical status of live FIPV2-challenged, vaccinated and LNP-immunized cats

Cat ID Code (Status)	Date [c]	BLOOD CHEMISTRY [ab]						COMPLETE BLOOD COUNT (CBC) [ab]							bAbs to	Fecal FIPV2 (cycle 2) [d]	Clinical Symptom [e]	Clinical Comments [f]
		Albumin 2.7 - 4.1 g/dL	Globulin 2.8 - 4.9 g/dL	A/G Ratio 0.6 - 1.2	Total Protein 6.2 - 8.2 g/dL	ALT 26 - 79 UL	ALP 8.5 - 33.5 U/L	RBC 7.7 - 11.6 M/μL	HGB 11.0 - 17.0 g/dL	PCV 33 - 48%	WBC 4.0 - 15.3 K/μL	Lymphocyte 1.1 - 5.9 K/μL	Neutrophil 2.0 - 9.1 K/μL	Eosinophil 0.1 - 1.2 K/μL				
**2FM** (vaccinated)	21 wpc	2.73	6.2	**0.4** L	**8.9** H	27	12	8.53	11.6	36	4.30	1.3	2.9	**0.04** L	S2 +	+	**Wt Loss 13%**	Abnormal A/G ratio and total protein
	23/24 wpc	**2.59** L	**7.6** H	**0.3** L	**10.2** H	44	**8** L	**7.36** L	**9.6** L	**32** L	7.01	**0.98** L	5.5	0.35	S2 ±	-	Wt loss 9%	Delay in abnormal CBC and blood chemistry
**2FP** (vaccinated)	21 wpc	3.10	4.1	0.8	**7.2** H	50	13	**7.22** L	4.3	34	**3.02** L	1.3	**1.5** L	0.12	S2 +	+	Wt Loss 5%	Abnormal Total Protein and RBC count
	23/24 wpc	2.85	**5.7** H	**0.5** L	**8.5** H	44	**8** L	**6.5** L	**9.9** L	**30** L	6.12	1.5	3.7	0.43	S2 +	+	Wt Loss 3%	Delay in abnormal CBC and blood chemistry
**FB1** (vaccinated)	21 wpc	3.64	3.4	1.1	7.0	**102** H	**36** H	10.23	14.3	41	9.53	2.7	5.7	0.57	S2 ±	+	Wt Loss 9%	Abnormal ALT and ALP (liver problem) [g]
	23/24 wpc	3.57	3.9	0.9	7.5	**91** H	**34** H	10.27	14.0	44	8.07	3.51	3.41	0.97	S2 ±	-	**Wt Loss 12%**	Abnormal ALT and ALP (liver problem) [g]
**2FL** (LNP-control)	21 wpc	3.4	3.7	0.9	7.1	62	24	8.18	11.1	33	**3.35** L	1.6	**1.3** L	**0.03** L	S2 +	+	Normal	Abnormal WBC and neutrophil count
	23/24 wpc	3.36	4.7	0.7	8.1	66	30	8.65	11.5	36	**3.68** L	1.7	**1.5** L	0.41	S2 +	-	Normal	Severer abnormal WBC and neutrophil count

[a] UF CVM Hospital Diagnostics; [b] Abbreviations: albumin/globulin (A/G), alanine aminotrasferase (ALT), alkaline phosphatase (ALP), red blood cell (RBC), hemoglobin (HGB), pack cell volume (PCV), white blood cell (WBC). [c] All paremeters performed at 24 wpc, except for fecal FIPV2 load at 23 wpc; [d] All negative (-) in cycle 1 at 23 wpc, except for 2FP in cycle 2, but all negative in both cycles at 27 wpc; [e] Weight loss (WT Loss) compared to weight at three days pre-challenge. Note that Figure S7A only shows weight up to 12 wpc for these cats. [f] Delay in abnormal outcome or severer abnormal outcome when compared to 21 wpc. [g] Most likely liver problem because responsive to treatment with Denamarin, which supports liver function.
